# Quantitative proteomic profiling of plasma alterations associated with thrombotic risk and insulin resistance in type 1 diabetes

**DOI:** 10.1055/a-2873-8027

**Published:** 2026-07-10

**Authors:** Claudia A. A. Harper, Jordan S. Marsh, Marco F. Fernandes, Rebecca C. Sagar, Silvia A. Synowsky, Hasnain Ahmed, Sally L. Shirran, Khalid M. Naseem, Ramzi A. Ajjan, Alan J. Stewart

**Affiliations:** 1 School of Medicine, University of St Andrews, St Andrews, UK; 2 Leeds Institute of Cardiovascular and Metabolic Medicine, University of Leeds, Leeds, UK; 3 Leeds Teaching Hospitals Trust, Leeds, UK; 4 Schools of Chemistry and Biology, Biomedical Sciences Research Complex, University of St Andrews, St Andrews, UK

**Keywords:** Clot structure, Haemostasis, Insulin resistance, Metabolic disorders, Proteomics

## Abstract

Type 1 diabetes is associated with prothrombotic changes that contribute to an elevated risk of cardiovascular disease. We hypothesised that insulin resistance (IR), increasingly prevalent among individuals with type 1 diabetes, exacerbates haemostatic abnormalities through distinct proteomic alterations in plasma. In a cross-sectional observational study, citrated plasma was collected from individuals with type 1 diabetes (with and without IR) and healthy controls. Fibrin clot formation and lysis were assessed using turbidimetric assays. Plasma proteomes were analysed using data-independent acquisition mass spectrometry (DIA-MS), and correlations between protein abundance and clinical parameters evaluated. Type 1 diabetes plasma exhibited faster clot initiation, increased clot density, and impaired fibrinolysis. Effects on clot lysis were most pronounced in individuals with IR. DIA-MS revealed distinct proteomic signatures, with significant changes in proteins involved in regulating clot structure and breakdown. Notably, clot density correlated positively with fibrinogen gamma-chain abundance. Type 1 diabetes is characterised by prothrombotic alterations that are further intensified by IR. Our findings reveal that these changes are driven by proteomic shifts affecting clot initiation, structural integrity, fibrinolysis, and inflammatory pathways, offering mechanistic insights and potential targets for mitigating cardiovascular risk in this population.

## Introduction

Type 1 diabetes (T1D) is characterised by autoimmune-mediated destruction of pancreatic beta cells, leading to absolute insulin deficiency and lifelong dependence on exogenous insulin therapy. Beyond its metabolic consequences, T1D is associated with a markedly increased risk of cardiovascular disease, which remains a leading cause of morbidity and mortality in this population [1–3]. Although traditionally associated with insulin sensitivity, a growing subset of individuals with T1D develop insulin resistance (IR), sometimes termed “double diabetes”, which is linked to obesity, poorer metabolic control, and an increased burden of vascular complications [4–6]. A 2022 study suggests that 16.8% of individuals with T1D exhibit IR [7]. While a more recent meta-analysis demonstrated that lower estimated glucose disposal rate (eGDR), indicating greater IR, predicts higher rates of CVDs and all-cause mortality in T1D, with hazard ratios around 0.83 per single unit increase in eGDR [8]. This confirms IR to be an independent cardiovascular risk factor within the T1D population. Double diabetes is linked to adverse cardiometabolic features including dyslipidaemia, hypertension, central adiposity, and weight gain from intensive insulin therapy, which together contribute to increased long-term CVD burden [9,10]. Overall, the evidence consistently shows that individuals with double diabetes carry substantially greater cardiovascular risk than those with T1D alone. Despite these observations, the mechanisms underpinning the heightened thrombotic risk in T1D, particularly in those with IR, remain incompletely understood.

A prothrombotic state is a major contributor to cardiovascular morbidity in diabetes [11,12]. T1D is often marked by alterations in fibrin clot structure and impaired fibrinolysis [13–16]. Hypofibrinolysis, a reduced ability to break down fibrin clots, is a key abnormality in T1D that significantly contributes to an increased risk of both macrovascular events such as myocardial infarction and stroke [17,18], and microvascular complications including retinopathy, nephropathy and neuropathy [19–21]. However, it is unclear to what extent these haemostatic disturbances differ between T1D patients with and without IR, and how these patterns compare to healthy individuals. Addressing this knowledge gap is critical for understanding the interplay between metabolic and vascular risk factors in T1D.

To investigate the molecular drivers of thrombotic risk in T1D, we adopted an integrative approach combining functional clotting and fibrinolysis assays with plasma proteomics. This analysis enables comprehensive characterisation of haemostatic changes in T1D compared to healthy controls, while also delineating the modifying influence of IR. By stratifying T1D individuals according to eGDR, a reliable marker of IR [22], we explored functional and proteomic differences between insulin-sensitive and insulin-resistant subgroups, providing insight into how IR shapes the prothrombotic phenotype in T1D.

## Methods

### Ethics

Blood was collected from T1D patients following approval by the Health Research Authority (Research Ethics Committee: 19/NE/0349, Integrated Research Application System: 259072). Healthy volunteers not on any treatment were recruited as control participants under ethics from University of Leeds (Medical Research Ethics Committee: 19-006). Written informed consent was obtained from all participants. Associated protocols were performed in accordance with the Declaration of Helsinki.

### Study design and participants

Citrated plasma was collected from 60 participants: 20 healthy controls, 20 individuals with T1D without IR (estimated glucose disposal rate (eGDR) >8), and 20 T1D individuals with IR (eGDR <6). This provides 80% power at α = 0.05 to detect a medium-to-large effect size (Cohen’s *f*=0.411). EDTA whole blood was used by the Blood Sciences Laboratory at Leeds General Infirmary (Leeds, UK) to measure glycated haemoglobin (HbA1c) using the International Federation of Clinical Chemistry and Laboratory Medicine (IFCC) Standard. Results are reported in mmol/mol with HbA1c concentrations of <42 mmol/mol (<6.0%) considered normoglycemic as per World Health Organisation criteria. The eGDR was calculated using the following formulae [23]:

eGDR (mg/kg/min) = 21.158 - (0.09 × waist circumference (cm) - (3.407 × HTN, 1 = Yes, 0 = No) - (0.551 × HbA1c (%)).

The inclusion criteria for the diabetes groups were: confirmed T1D diagnosis (≥3 years, supported by clinical history, autoantibodies and/or low C-peptide), current insulin treatment, age 18–39 years, and BMI ≥18 kg/m^2^. Exclusion criteria included end-stage renal disease, malignancy, pregnancy, and use of anticoagulant or antiplatelet medications. Lipids and creatine were measured by Leeds Pathology, Blood Sciences, Leeds Teaching Hospitals NHS Trust, Leeds, UK. Cholesterol was measured using the Atellica CH Chol-2 assay (Siemens, Erlangen Germany), high-density lipoprotein cholesterol (HDL-C) using the Atellica CH HDL cholesterol assay (Siemens) and triglycerides using Atellica CH Triglycerides_2 assay (Siemens). low-density lipoprotein cholesterol (LDL-C) was calculated using the Friedewald equation. Creatinine was measured using the Atellica CH Enzymatic Creatinine_3 assay (Siemens). C-reactive protein (CRP) was measured using a Human C Reactive Protein ELISA assay kit (ab99995; Abcam, Cambridge, UK). Key demographic data were recorded, with participant characteristics summarised and compared in Table 1.

### Fibrin clotting and clot lysis assays

Fibrin clot formation and lysis were measured using a validated turbidimetric assay [24,25]. Plasma (25 μL) was loaded in duplicate into a 96-well plate and mixed with 75 μL of lysis buffer containing tissue plasminogen activator (tPA). After 3 minutes, 50 μL of activation buffer was added, yielding final concentrations of 0.03 U/mL thrombin, 7.7 mM CaCl_2_, and 0.35 μg/mL tPA, with plasma diluted 1:6. Absorbance at 340 nm was recorded every 12 s using a Clariostar platereader (BMG Labtech, Ortenberg, Germany). Turbidity curves describing fibrin polymerisation and subsequent fibrinolysis were generated, from which several parameters were extracted: lag time, representing the initiation phase before measurable fibrin formation (i.e., the onset of clotting); maximum absorbance, reflecting the size and density of the formed fibrin clot; time to 50% lysis, indicating the rate at which the clot undergoes plasmin-catalysed breakdown; and clot lysis area (area under the curve from peak turbidity back to baseline), which integrates both clot formation and lysis kinetics and serves as a composite measure of overall clot density, stability and resistance to fibrinolysis. Prior to analysis, outliers were identified and removed using the ROUT method (Q = 1%). Clot turbidity assay calculations were then performed on the cleaned data. Group comparisons between control and T1D were assessed using the Mann–Whitney U test, while differences across control, T1D without IR, and T1D with IR groups were analysed using one-way ANOVA for equal variances.

### Sample preparation and mass spectrometry analysis

For proteomic analysis, 50 μg of total protein was denatured in 50 mM ammonium bicarbonate, 5 M urea, and 2% sodium deoxycholate. Proteins were reduced with 5 mM Tris(2-carboxyethyl)phosphine, alkylated with 10 mM iodoacetamide, and quenched with 20 mM dithiothreitol. Samples were diluted to 1.5 M urea and digested overnight with trypsin (1:50 w/w; Promega, Southampton, UK) at 30°C. Peptides were acidified (0.5% trifluoroacetic acid), desalted using C18 SepPak cartridges (Waters, Elstree, UK), and dried using a SpeedVac (ThermoFisher Scientific, Paisley, UK). A portion of each sample was reserved for DIA-MS, with the remainder pooled for spectral library generation. The solvent in each sample was removed using a SpeedVac (ThermoFisher Scientific, Loughborough, UK). Data-independent acquisition mass spectrometry (DIA-MS) was performed on 3 μg of peptides from each plasma or platelet sample accordingly.

### Spectral Library Generation

Peptides used for the library generation were separated using high-pH reversed-phase chromatography. The pooled, dried peptides were then resuspended in 100 μL buffer A, consisting of 10 mM ammonium formate, 2% acetonitrile, pH 10.0. Peptides were then fractionated on a XBridge C18 column (4.6 × 100 mm, 5 μm, Waters) at 1 ml/min with the following gradient: linear gradient of 4–28% buffer B (10 mM ammonium formate, 90% acetonitrile, pH 10.0) for 36 min, then 28–50% B for 8 min, followed by 100% B for a further 5 min to wash the column, before re-equilibration in 100% buffer A for 10 min. Fractions (0.5 mL every 30 s) were combined into 25 pools, dried, and resuspended in 0.1% formic acid. The fractions were analysed individually on a Thermo Fusion Lumos Tribrid system mass spectrometer coupled to an UPLC3000 system in a nanoflow configuration, in data-dependent mode, to achieve in depth identification of proteins. Additionally, as a control, peptides from each individually digested sample were combined and analysed in data dependent mode. Prior to mass spectrometric analysis, reference indexed retention time (iRT) peptides (Biognosys, Schlieren, Switzerland) were added to each sample according to the manufacturer’s specifications to allow correction of retention times. The samples were loaded in loading buffer (100% water, 0.05% TFA) and bound to an Acclaim Pepmap 100 μm × 2 cm trap (ThermoFisher Scientific), and washed for 10 min to waste, after which the trap was turned in-line with the Easy Spray analytical column (Acclaim Pepmap RSLC 75 μm × 50 cm). The analytical solvent system consisted of buffer A (100% water, 0.1% FA in water) and buffer B (20% water, 0.1% FA in acetonitrile) at a flow rate of 300 nl/min, Peptides were eluted from the column using a linear solvent gradient using the following gradient: linear 4–35% of buffer B over 140 min, linear 35–60% of buffer B for 10 min, sharp increase to 95% buffer B within 0.1 min, isocratic 95% of buffer B for 15 min, sharp decrease to 2% buffer B within 0.1 min and isocratic 2% buffer B for 15 min. The field asymmetric ion mobility spectrometry (FAIMS) interface alternated between -45 and -65V The mass spectrometer was operated in data-dependent acquisition (DDA) positive ion mode with a cycle time of 1.5 s. The Orbitrap was selected as the MS1 detector at a resolution of 120,000 with a scan range of from m/z 375 to 1500. Peptides with charge states 2 to 5 were selected for fragmentation in the ion trap using higher-energy collisional dissociation as collision energy.

The raw data files were converted into Mascot Generic Format (MGF) using MSconvert (ProteoWizard). Spectra were searched using Mascot with trypsin specified as the cleavage enzyme, allowing up to one missed cleavage. Carbamidomethylation of cysteine was set as a fixed modification, and oxidation of methionine was included as a variable modification. Searches were performed against the SwissProt database restricted to *Homo sapiens*, with iRT peptides appended. The precursor mass tolerance was set to 20 ppm, and the fragment ion mass tolerance to 0.6 Da.

### DIA-MS and Data Analysis

For DIA-MS data acquisition of plasma and platelet proteins, the same mass spectrometer and LC-MS/MS setup was used as described above, operated in DIA mode. The DIA method alternated between a full MS scan and a targeted MS2 (tMS2) scan comprising 24 scan windows. FAIMS was employed with compensation voltages of -45 V and -65 V. MS1 scans were acquired in the Orbitrap at a resolution of 120,000 over an m/z range of 400–1000. The tMS2 scan uses HCD as activation energy with fragments detected in the Orbitrap at 30,000 resolution. The isolation scheme included 20 windows of 20 m/z from 410–790, followed by sequentially wider windows: 30 m/z (790–820), 40 m/z (820–860), 50 m/z (860–910), and 60 m/z (910–970).

### Proteomic data analysis and representation

The raw spectral data files from both DDA and DIA modes were converted to mzML format using ProteoWizard version 3.0. In each case, a spectral library was generated from pooled DDA data using FragPipe version 21.0. The FASTA file employed contained the complete human reference proteome (UniProt accession: UP000005640), plus Biognosys iRT peptides and sequences appended from a universal contaminant library [26]. Protein identification and quantification were conducted using DIA-NN version 1.8.1 with canonical trypsin/P specificity, allowing for one missed cleavage. Variable modifications were set to N-terminal methionine excision, carbamidomethylation of cysteine residues, and oxidation of methionine. The precursor charge state was restricted to the range of 1–4, with precursor m/z ranging from 300 to 1800 and fragment ions m/z ranging from 200 to 1800. The ‘match-between-runs’ (MBR) option was enabled, and the global false discovery rate (FDR) for precursors was set to less than 1%. The respective previously generated spectral libraries were selected for the analyses.

Protein data were exported from DIA-NN as Protein Groups Matrix file. Entries flagged as potential contaminants were removed, excluding 52 proteins. Proteins were retained if present in at least 50% of samples within each eGDR group. Missing values were imputed by group using missRanger with predictive mean matching (pmm.k = 5). Imputed intensities were log_2_ transformed and quantile normalised. Differential abundance was tested with limma linear models using a no intercept design (~0 + Group) or (~0 + Group + Sex) and contrasts for T1D versus controls and T1D with IR versus without IR. P-values were adjusted using the Benjamini Hochberg method. Pearson correlations were calculated between each protein and haemostatic parameters. Variables were centred and scaled, and correlation p-values were FDR corrected. Leave-one-out sensitivity analysis was performed to assess the robustness of limma-based protein associations by refitting the model while omitting one sample at a time. Using the same design and contrasts, log_2_ fold changes from each iteration were compared to the full model. Robustness was evaluated via correlation, effect size changes, direction shifts, and loss of significance.

## Results

### Comparison of demographic and clinical data between groups

Demographic and clinical parameter data were collected and median values compared between the groups (Table 1). The individuals with T1D were slightly older than the controls (median age of 24.2 vs 21.0 years; p=0.0009) and had higher BMI (26.1 vs 23.3 kg/m^2^; p=0.0016). Glycaemic control differed markedly, with higher HbA1c levels in T1D (62.0 vs 33.5 mmol/mol; p<0.0001) and lower eGDR (waist circumference-based) in T1D (7.3 vs 11.1; p<0.0001), consistent with reduced estimated insulin sensitivity. Inflammatory and renal biomarkers also differed between groups. The CRP level was higher in T1D (1.5 vs 0.2 mg/L; p<0.0001), while creatinine was lower (68.0 vs 92.0 µmol/L; p<0.0001). By contrast, there were no statistically significant differences in sex distribution (75% vs 60% male; p=0.23) or lipid measures (total cholesterol, HDL-C, LDL-C, triglycerides; all p>0.10).

Within the T1D cohort, participants classified as insulin resistant had a higher BMI (27.3 vs 24.2 kg/m^2^; p=0.0051), higher triglycerides (1.3 vs 0.8 mmol/L; p=0.014), and higher HbA1c (71.0 vs 54.5 mmol/mol; p=0.0009) than those without IR. There were a significantly higher proportion of males in the T1D with IR group (90% vs 60%; p=0.0285). As expected, eGDR was substantially lower in the insulin-resistant group (5.4 vs 10.1; p<0.0001). CRP was also higher in the insulin-resistant subgroup (2.5 vs 1.1 mg/L; p=0.030), whereas age, total cholesterol, HDL-C, LDL-C, and creatinine did not differ significantly (all p≥0.35).

### Type 1 diabetes and IR alter clot formation and lysis

**Figure 1:**
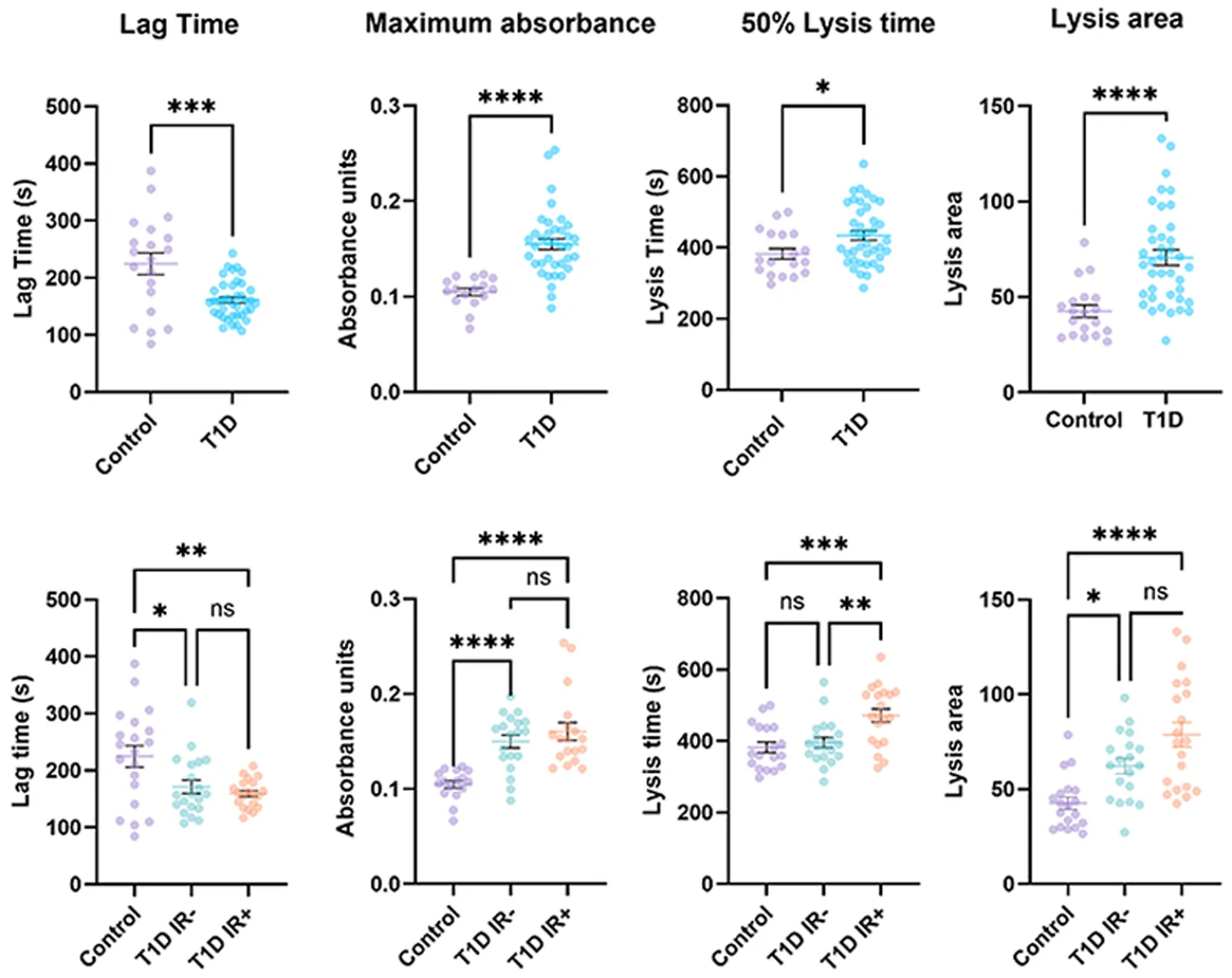
Fibrin clotting and lysis parameters in type 1 diabetes and control participants. Comparisons between clot lag time, maximum absorbance, 50% lysis time and lysis area in (A) type 1 diabetes (T1D) versus healthy controls, in (B) T1D with IR (IR+; eGDR<6) and T1D without IR (IR-; eGDR >8) versus healthy control plasma samples. Assays were performed in triplicate. Statistical comparisons were performed using Student’s T-test. Significance levels are annotated as follows: ^****^ P <0.0001; ^***^ p <0.001; ^**^ p <0.01; ^*^ p <0.05; ns indicates p >0.05.

To better understand how haemostatic changes in T1D and IR affect fibrin clot behaviour, we compared clot formation and lysis parameters across groups. Marked differences in fibrin clot characteristics were observed between individuals with T1D and controls (Figure 1A). T1D samples showed faster initiation of clotting, with a median lag time of 161.3 s compared to 224.6 s in controls. A higher median maximum absorbance (0.153 abs. units compared to 0.108 abs. units in controls) was also observed with the T1D samples. An increased median 50% lysis time in T1D (414.5 s compared to 361.7 s in controls), was consistent with the formation of larger, denser fibrin networks that are more resistant to breakdown. Lysis area was 57.7% greater in T1D samples compared to controls, reflecting both the altered clot structure and breakdown profile.

Subgroup analysis (Figure 1B) revealed that median 50% clot lysis time was slower in T1D individuals with IR (471.4 s) compared to those without IR (396.1 s) or controls (382.4 s). Lysis area was 29.9% greater in T1D individuals with IR compared to those without IR. However, the mean lag time and maximum absorbance did not differ between these subgroups. No difference in lysis time was observed between controls and T1D individuals without IR.

### Plasma protein alterations associated with type 1 diabetes and IR

**Figure 2:**
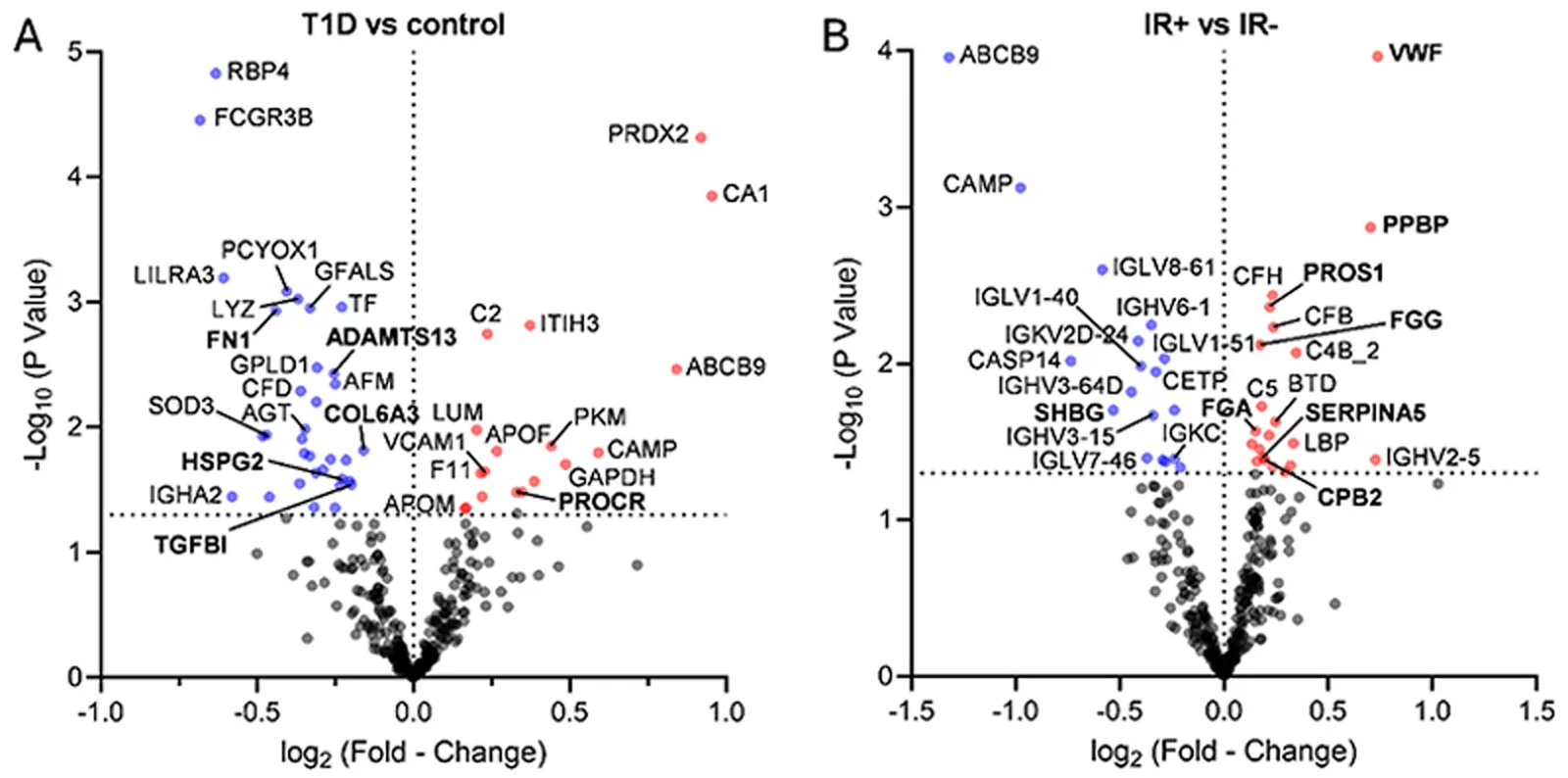
Comparative analysis of plasma protein abundance in type 1 diabetes and control participants and correlation with haemostatic parameters. (a and b) Volcano plots across clinical groups. Each panel shows comparisons between groups: (A) type 1 diabetes (T1D) vs control, (B) IR+ vs control, IR- vs control, and IR+ vs IR-. The x-axis represents the log_2_ fold-change, and the y-axis represents -log_10_ p-value. Points are coloured according to statistical significance: red indicates significantly upregulated proteins, blue indicates significantly downregulated proteins, and grey denotes nonsignificant changes (thresholds: |log_2_ FC| > log_2_(0) and p <0.05). Dashed vertical and horizontal lines mark the null fold-change (0) and the p-value cutoff, respectively. Gene labels are displayed for proteins meeting significance criteria. T1D (eGDR <6 and eGDR >8), IR+ (eGDR <6), and IR-(eGDR >8).

Quantitative DIA-MS was employed to compare plasma proteomes between T1D patients and controls, as well as between T1D subgroups (with and without IR). This approach enabled comprehensive profiling across all samples, identifying 317 unique proteins. Relative abundances were assessed between T1D and control groups, revealing significant differences (p ≤ 0.05) in 51 proteins: 33 were lower and 18 higher in T1D samples compared to controls (Figure 2A). Full results including adjusted p-values are provided in Table S1. Peroxiredoxin 2 (PRDX2) and carbonic anhydrase 1 (CA1) showed the greatest relative enrichment in T1D plasma. Other proteins with higher prior abundance in T1D included factor XI (F11), endothelial protein C receptor (PROCR), and several apolipoproteins (APOA4, APOD, APOF, APOH). Proteins with lower abundance in T1D included retinol-binding protein 4 (RBP4) and Fc gamma receptor IIIb (FCGR3B), as well as von Willebrand factor-cleaving protease (ADAMTS13), extracellular matrix and adhesion-related proteins such as perlecan (HSPG2), collagen VI (COL6A3), transforming growth factor beta-induced (TGFBI), and fibronectin (FN1).

Within the T1D cohort, comparison of protein abundances in individuals with and without IR revealed 39 proteins with differential levels across these groups. Of these, 21 were higher and 18 lower in those with IR compared to those without IR (Figure 2B). Full results including adjusted p-values are provided in Table S2. Von Willebrand factor (VWF) and pro-platelet basic protein (PPBP) showed the greatest relative enrichment in the IR subgroup, along with fibrinogen alpha and gamma chains (FGA and FGG), protein S (PROS1), protein C inhibitor (SERPINA5), and complement factors C5 and CFH. Proteins with lower relative abundance in IR included ATP-binding cassette subfamily B member 9 (ABCB9) and cathelicidin antimicrobial peptide (CAMP), sex hormone binding globulin (SHBG), cholesterol ester transfer protein (CETP), and several immunoglobulin proteins.

There was no significant difference between the proportion of males between control and T1D groups. However, a significant difference was observed between T1D with IR relative and those without IR (90% versus 60%; p=0.0285). Given this difference, additional analyses of proteomic differences adjusted for sex were performed. The corresponding results for T1D individuals versus controls, and for T1D with IR versus without IR are shown in Tables S3 and S4, respectively. In both comparisons, the individual proteins highlighted above remained significantly altered after adjustment for sex, indicating that the observed differences were not attributable to a sex imbalance.

To assess model generalisability, we conducted a leave-one-out sensitivity analysis (Figure S1) using limma-derived protein abundance estimates. Full-model log_2_ fold-change estimates (Figure S1A) were compared with leave-one-out log_2_ fold-change estimates for each protein across the contrasts. Here, high concordance with the diagonals showing that differential abundance estimates were robust to the procedure. Low sample-level influence (Figure S1B) in the leave-one-out sensitivity analysis procedure with only one labelled sample point outside of the interquartile range criterion.

### Associations between haemostatic measures and protein abundance

**Figure 3:**
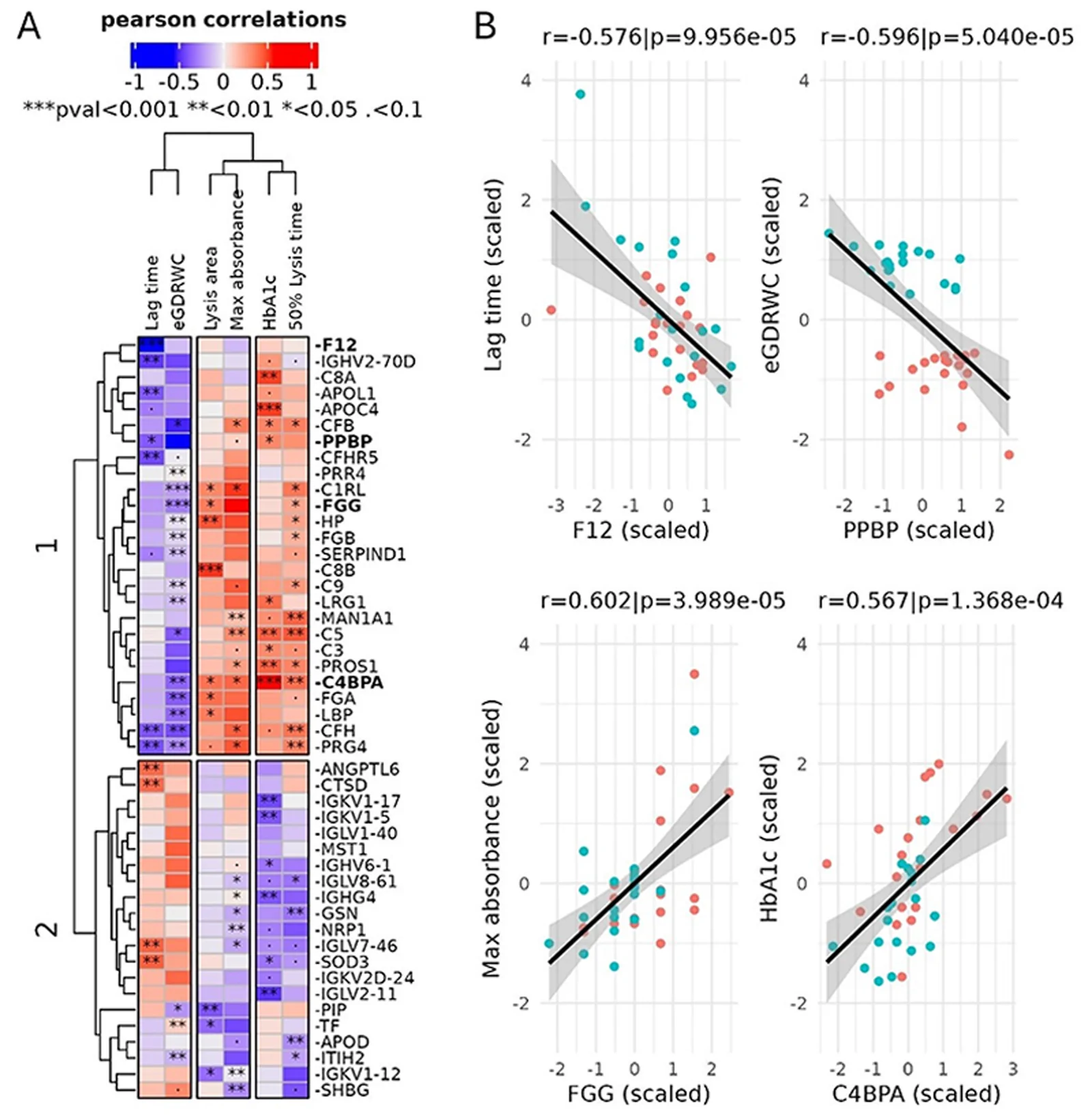
Associations between plasma proteins and haemostatic parameters. (A) Heatmap of Pearson correlation coefficients (ρ) between measured proteins and parameters including fibrin clotting and lysis parameters, eGDRWC (eGDR measured using waist circumference) and BMI. Data were filtered to p-values <0.01 for clarity (full results in Table S5). Significance levels are annotated in each cell (^***^ p <0.001; ^**^ p <0.01; ^*^ p <0.05; · p <0.1, where “·” indicates 0.05 ≤ p <0.1). Protein (rows) in bold indicate q-values <0.1. Colour coding shows correlation strength and direction, with red for strong positive correlations (ρ ≈ +1) and blue for strong negative correlations (ρ ≈ -1). Rows and columns are hierarchically clustered, with dendrograms indicating similarity structure. (B) Scatter plots showing significant correlations for F12, PPBP, FGG, and C4BPA, with both axes scaled to mean 0 and standard deviation 1. Points are coloured by eGDR category ("eGDR <6" = "eGDR_low" is red, "eGDR >8" = "eGDR_high" is green). The black line represents the fitted linear regression model, with the shaded area indicating the 95% confidence interval of the fit. Pearson correlation coefficients and p-values are shown in plot subtitles.

Correlations between haemostatic measures and plasma protein abundances in T1D revealed several notable associations (Figure 3A; Table S5), suggesting links between coagulation dynamics, vascular health, and metabolic status in T1D. Reduced clot lag time, a marker of accelerated clot initiation, correlated negatively with factor XII (F12; Figure 3B). Pro-platelet basic protein (PPBP), a platelet-derived chemokine, showed a negative correlation with estimated glucose disposal rate (eGDR), indicating that higher PPBP levels may be associated with greater IR and platelet activation. Clot maximum absorbance, reflecting fibrin network density, correlated positively with fibrinogen gamma-chain (FGG), reinforcing the structural role of fibrinogen in clot architecture. Finally, C4b-binding protein (C4BPA) levels correlated positively with HbA1c, suggesting a link between chronic glycaemic exposure and complement system activation.

## Discussion

Our findings indicate that IR contributes to hypofibrinolysis in T1D but has limited impact on the accelerated clot initiation or increased clot size and density. Notably, CRP levels were higher in the T1D with IR group, suggesting an inflammatory contribution that may exacerbate the observed impairment in fibrinolysis. Proteomic analysis suggests these characteristics stem from coordinated changes in proteins regulating coagulation, extracellular matrix remodelling, and endothelial function. However, note that differences in age, adiposity, glycaemic control, and inflammatory status between groups may have influenced the proteomic profiles obtained using these samples, and should therefore be considered when interpreting the observed differences.

Matrix proteins including FN1, COL6A3 and HSPG2 were less abundant in T1D. This may reflect a shift from a matrix that is relatively rich in these proteins toward alternative structural components. This may contribute to a more compact and less porous clot and contribute to the enhanced clot density observed in the plasma taken from T1D patients compared to controls. Notably, plasma levels of the VWF-cleaving protease ADAMTS13 were lower in T1D. Lower levels of this enzyme may permit the persistence of ultra-large VWF multimers and thereby enhance clot stability. This effect is likely accentuated in the insulin-resistant subgroup, where VWF concentrations were also elevated. Interestingly, the plasma levels of ADAMTS13 and VWF have been shown to be inversely linked [27]. Plasma levels of PROCR in T1D were also found to be elevated. This receptor normally binds protein C and activated protein C (APC), which in turn inactivates factors Va and VIIIa, to limit thrombin generation and clot formation. However, as the protein detected is derived from plasma, it is likely that this represents the soluble form of the protein, which can act as a decoy receptor to compete with the membrane-bound receptor form for protein C binding to help induce a procoagulant state [28]. Consistent with our data, elevated levels of the soluble form of the receptor have previously been associated with T1D [29].

In individuals with T1D and IR (eGDR <6), proteomics revealed elevated plasma levels of FGA, FGG, PROS1, carboxypeptidase B2 (CPB_2), and SERPINA5 compared to those without IR (eGDR >8). The elevated levels of fibrinogen chains FGA and FGG suggest that the IR sub-group may have a greater tendency for clot formation and the formation of more stable clots. High plasma fibrinogen levels are well known to associate with cardiovascular disease risk [30–32], and more specifically, have been shown to be linked to venous thrombosis in the elderly [33]. Also, fibrinogen is an acute-phase protein [34], so higher levels often reflect systemic inflammation. PROS1 enhances APC-mediated inactivation of factors Va and VIIIa [35]; its increase, as with soluble PROCR, may further compromise APC functioning and promote a procoagulant state. CPB2 regulates fibrinolysis by cleaving lysine residues essential for plasminogen binding [36], and its dysregulation may impair clot breakdown. While SERPINA5 modulates proteases including APC and thrombin [37], meaning that increased levels in IR may further destabilise haemostatic control. Many of the proteins that exhibited reduced expression in those with IR compared to those without were immunoglobulins, which may indicate suppression of adaptive immune response in IR. SHBG levels were also reduced in T1D individuals with IR. This protein has been reported to be an indicator of reduced insulin sensitivity, and both high and low levels of this protein have been associated with increased cardiovascular risk [38]. Collectively, these proteomic alterations highlight a further impaired anticoagulant and fibrinolytic capacity in T1D with IR, potentially contributing to reduced clot lysis and elevated thrombotic risk.

In T1D, clot lag time negatively correlated with plasma levels of F12, a key intrinsic pathway initiator [39]. Although F12 acts upstream of thrombin generation, the clotting assays used were initiated with thrombin, suggesting this association reflects downstream effects on clot structure or fibrinolysis rather than direct modulation of initiation. Nonetheless, reduced F12 may contribute to faster clot formation in vivo under inflammatory or contact-activated conditions. PPBP, a protein released upon platelet activation and known to stimulate plasminogen activator synthesis [40], negatively correlated with eGDR, potentially linking IR to altered fibrinolytic regulation in vivo. Clot maximum absorbance correlated positively with FGG, a key structural component of clots, indicating that the elevated levels contribute to the larger and denser clots formed in the turbidity experiments. The levels of C4BPA, a key regulator of the complement system that aids control of inflammation and immune reposes [41], correlated positively with HbA1c, potentially reflecting increased inflammation associated with poor glycaemic control.

In summary, hypofibrinolysis in T1D is linked to IR, while accelerated clot initiation and increased clot density are primarily driven by coordinated changes in coagulation, extracellular matrix, and endothelial proteins. These findings underscore that thrombotic risk in T1D is driven by distinct molecular mechanisms, amplified by IR. Therapeutic strategies targeting ADAMTS13 activity, fibrinolysis regulators such as CPB2 and SERPINA5, or extracellular matrix composition may help mitigate this risk. Moreover, proteomic markers may be useful for stratifying patients and guiding personalised interventions. The potential shift in proteins with potential to contribute to clot architecture also suggests that future antithrombotic approaches should consider matrix remodelling alongside conventional coagulation pathways.

## Supplementary Material

Table 1

## Figures and Tables

**Table 1:** Demographic information for T1D and control participants in plasma experiments. Comparisons of age, sex, BMI, lipid profiles, HbA1c, eGDR, creatine and CRP between control and T1D groups and between T1D without and with IR were performed for participants in the plasma experiments. Median values are provided with interquartile ranges (IQRs) in parentheses. Sex comparisons were carried out using Chi-square test. Other statistical comparisons were performed using a Mann-Whitney U test.

T1D versus control individuals			
Parameter	Control (median (IQR)); n=20	T1D (median (IQR)); n=40	P value
Age (years)	21.0 (21.0–22.8)	24.2 (22.8–26.9)	0.0009
Sex (n; % male)	12; 60%	30; 75%	0.23
BMI (kg/m^2^)	23.3 (21.6–25.0)	26.1 (23.7–29.0)	0.0016
Total cholesterol (mmol/L)	3.8 (3.6–4.9)	4.4 (3.9–5.1)	0.12
HDL-C (mmol/L)	1.4 (1.1–1.6)	1.4 (1.1–1.7)	0.71
LDL-C (mmol/L)	2.1 (1.9–2.6)	2.4 (1.9–3.0)	0.45
Triglycerides (mmol/L)	1.0 (0.7–1.1)	0.9 (0.7–1.3)	0.77
HbA1c (mmol/mol)	33.5 (32.0–37.0)	62.0 (53.2–74.5)	<0.0001
eGDR (WC)	11.1 (10.5–11.6)	7.3 (5.4–10.1)	<0.0001
Creatinine (μmol/L)	92.0 (81.0–96.5)	68.0 (60.0–74.0)	<0.0001
CRP (mg/L)	0.2 (0.1–1.0)	1.5 (0.7–4.3)	<0.0001
**T1D with IR versus T1D without IR**			
**Parameter**	**T1D without IR (median (IQR)); n=20**	**T1D with IR (median (IQR)); n=20**	**P value**
Age (years)	24.1 (22.8–27.3)	24.4 (22.8–26.9)	0.89
Sex (n; % male)	12; 60%	18; 90%	0.0285
BMI (kg/m2)	24.2 (23.1–27.1)	27.3 (24.9–33.1)	0.0051
Total cholesterol (mmol/L)	4.1 (3.6–5.0)	4.4 (4.0–5.3)	0.41
HDL-C (mmol/L)	1.5 (1.2–1.7)	1.4 (1.0–1.6)	0.47
LDL-C (mmol/L)	2.2 (1.8–2.9)	2.5 (2.0–3.2)	0.35
Triglycerides (mmol/L)	0.8 (0.6–1.0)	1.3 (0.8–1.8)	0.014
HbA1c (mmol/mol)	54.5 (48.0–64.5)	71.0 (58.0–81.0)	0.0009
eGDR (WC)	10.1 (9.2–10.6)	5.4 (4.4–5.7)	<0.0001
Creatinine (μmol/L)	65.0 (60.2–71.8)	71.0 (58.0–78.0)	0.37
CRP (mg/L)	1.1 (0.5–3.7)	2.5 (1.1–7.4)	0.030

## Data Availability

The proteomic data are available and have been deposited to the ProteomeXchange Consortium via the PRIDE Partner Repository with the dataset identifier PXD068128.

## What is already known about the topic

- Type 1 diabetes (T1D) is linked to prothrombotic changes that increase cardiovascular disease risk.
- Insulin resistance (IR) is becoming more common in T1D and is suspected to worsen haemostatic abnormalities.

### What does the paper add

- Demonstrates that T1D plasma shows faster clot initiation, denser fibrin clots, and impaired fibrinolysis than controls, with the fibrinolytic effect being most strongly evident in T1D individuals with IR compared to those without.
- Identifies distinct proteomic signatures associated with T1D and the modifying effect of IR via DIA-MS, highlighting differentially abundant proteins involved in clot structure, breakdown, and inflammation.
- Provides mechanistic insights into changes that occur, suggesting potential therapeutic targets for reducing cardiovascular risk.

## Abbreviations

ADAMTS13: von Willebrand factor-cleaving protease
APC: activated protein C
C4BPA: C4b-binding protein
COL6A3: collagen VI
CPB2: carboxypeptidase B2
CRP: C-reactive protein
DDA-MS: data-dependent acquisition mass spectrometry
DIA-MS: data-independent acquisition mass spectrometry
eGDR: estimated glucose disposal rate
F12: factor XII
FAIMS: field asymmetric ion mobility spectrometry
FCGR3B: Fc gamma receptor IIIb
FDR: false discovery rate
FGG: fibrinogen gamma-chain
FN1: fibronectin
HDL-C: high-density lipoprotein cholesterol
HHSPG2: perlecan
IR: insulin resistance
iRT: indexed retention time
LDL-C: low-density lipoprotein cholesterol
PPBP: pro-platelet basic protein
PROCR: endothelial protein C receptor
PROS1: proteins S
SERPINA5: protein C inhibitor
T1D: type 1 diabetes
TGFBI: transforming growth factor beta-induced
tPA: tissue plasminogen activator
VWF: von Willebrand factor.
