# Supplementary material for: Quantitative proteomic profiling of plasma alterations associated with thrombotic risk and insulin resistance in type 1 diabetes
Source: Thromb Haemost. Author manuscript; Available in PMC 2026 Oct 24. (PMC7619632; doi:10.1055/a-2873-8027)
Supplement: Supplementary file 1 [file EMS217227-supplement-Table_1.pdf]

Supplementary Information

Quantitative proteomic profiling of plasma alterations associated with thrombotic risk and insulin resistance in type 1 diabetes

Claudia Harper, Jordan S. Marsh, Marco F. Fernandes, Rebecca C. Sagar, Silvia A. Synowsky, Hasnain Ahmed, Sally L. Shirran, Khalid M. Naseem, Ramzi A. Ajjan and Alan J. Stewart

Table S1. Comparison of plasma protein abundance between type 1 diabetes (both with and without IR) group versus control group.

Table S2. Comparison of plasma protein abundance between type 1 diabetes with IR group versus type 1 diabetes without IR group.

Table S3. Comparison of plasma protein abundance between the type 1 diabetes group (both with and without IR)) and the control group, adjusted for sex as a covariate.

Table S4. Comparison of plasma protein abundance between type 1 diabetes with IR group versus type 1 diabetes without IR group, adjusted for sex as a covariate.

Fig S1 Leave-one-out sensitivity analysis of limma-derived protein abundance estimates.

Table S5. Plasma protein correlations with haemostatic parameters.

Thromb Haemost | DOI 10.1055/a-2873-8027 | © Thieme. All rights reserved.

Table S1. Comparison of plasma protein abundance between type 1 diabetes (both with and without IR) group versus control group. Differential protein abundance was assessed using linear regression models (protein ~ group), with the pairwise contrast type 1 diabetes vs control. P-values were adjusted for multiple testing using the Benjamini–Hochberg false discovery rate (FDR), with significance defined as p <0.05.

Protein (gene name)	UniProtID	logFC	AveExpr	t	P.Value	adj.P.Val	B
RBP4	P02753	-0.632632038	22.65803251	-4.717064681	1.49E-05	0.001584972	2.927234956
FCGR3B	O75015	-0.683347916	17.35149789	-4.474975442	3.50E-05	0.002799137	2.125505163
PRDX2	P32119	0.919259347	17.34949781	4.381674753	4.84E-05	0.003099491	1.821848686
CA1	P00915	0.954056522	18.30045027	4.066358612	0.000142027	0.007574751	0.819963161
LILRA3	Q8N6C8	-0.608348005	16.18974434	-3.604050995	0.000640328	0.029272143	-0.571706931
FN1	P02751	-0.441021214	23.31313353	-3.409207617	0.001173693	0.031298467	-1.126886642
IGFALS	P35858	-0.331122978	21.2678023	-3.427485668	0.001109704	0.031298467	-1.07566081
LYZ	P61626	-0.370018273	20.01423702	-3.480472841	0.000942376	0.031298467	-0.926144434
PCYOX1	Q9UHG3	-0.406031752	19.12315255	-3.525718552	0.000818738	0.031298467	-0.797294031
TF	P02787	-0.229595715	27.75701368	-3.432283978	0.001093462	0.031298467	-1.062183189
ITIH3	Q06033	0.37239651	21.19682569	3.321224511	0.001533694	0.037752474	-1.370906148
C2	P06681	0.236616424	20.96550568	3.269381147	0.001792261	0.040965972	-1.512670652
ABCB9	Q9NP78	0.841658786	19.75621556	3.048784687	0.003423284	0.068465688	-2.09843113
GPLD1	P80108	-0.308750181	20.97915714	-3.057092982	0.00334245	0.068465688	-2.076896854
ADAMTS13	Q76LX8	-0.254318516	16.33400311	-3.02041221	0.003713288	0.069897181	-2.171650465
AFM	P43652	-0.250391092	22.53667808	-2.951100864	0.004520751	0.080368912	-2.348418372
CFD	P00746	-0.360949971	19.0087585	-2.906748345	0.00512003	0.086232088	-2.459946111
IGFBP3	P17936	-0.31038507	20.07126641	-2.834589014	0.006254304	0.100068868	-2.638700746
AGT	P01019	-0.34685811	23.1739123	-2.649657719	0.010299486	0.145709083	-3.081121763
LUM	P51884	0.20243806	22.90920827	2.643334621	0.01047284	0.145709083	-3.095839651
IGHV3OR15-7	A0A075B7D8	-0.483743466	15.74683607	-2.599024881	0.011764216	0.150581963	-3.198201125
SOD3	P08294	-0.470487995	17.69364548	-2.607295746	0.011512689	0.150581963	-3.179197748
LTF	P02788	-0.356732379	17.56903675	-2.579084362	0.012391302	0.152508332	-3.243820707
APOF	Q13790	0.266091877	19.91760593	2.493360481	0.015447161	0.167034987	-3.436745368

© Thieme. All rights reserved.

CAMP	P49913	0.590682868	18.62764853	2.481920064	0.015902772	0.167034987	-3.462096604
COL6A3	P12111	-0.159578131	17.19796398	-2.500245133	0.015178692	0.167034987	-3.421444188
FETUB	Q9UGM5	-0.350019458	20.10634803	-2.475065684	0.016181514	0.167034987	-3.477240423
PKM	P14618	0.440659378	16.39161078	2.528213607	0.014130843	0.167034987	-3.358936004
CNDP1	Q96KN2	-0.331769692	19.30167685	-2.456190768	0.016971979	0.169719791	-3.518766959
APOC1	P02654	-0.265438544	22.25240657	-2.433308754	0.017976738	0.172008963	-3.56876372
HABP2	Q14520	-0.2157805	18.8936584	-2.426716594	0.018275952	0.172008963	-3.58309687
GAPDH	P04406	0.486759704	17.56418693	2.395147161	0.019771718	0.180769996	-3.651297476
F11	P03951	0.214737309	20.09897892	2.329309184	0.023248414	0.190756214	-3.791167843
IGHV1-2	P23083	-0.28941788	18.375055	-2.354916515	0.021836191	0.190756214	-3.737147623
LGALS3BP	Q08380	-0.312687745	19.3737818	-2.331602891	0.023118681	0.190756214	-3.786349038
VCAM1	P19320	0.226564883	18.72768101	2.343525233	0.022454682	0.190756214	-3.76123846
APMAP	Q9HDC9	0.386206833	18.80508546	2.267795153	0.026978031	0.205546899	-3.918929995
HSPG2	P98160	-0.228925433	16.28334575	-2.284362106	0.025924718	0.205546899	-3.884801301
TTR	P02766	-0.205331689	25.60641844	-2.270130738	0.026827278	0.205546899	-3.914131147
PIP	P12273	-0.364268428	16.9164681	-2.251329546	0.028062253	0.206996429	-3.952644004
SAA4	P35542	-0.23408151	22.9353759	-2.235959882	0.029108873	0.206996429	-3.983928095
TGFBI	Q15582	-0.199951813	18.80732595	-2.242645895	0.028649418	0.206996429	-3.970341192
LDHA	P00338	0.346893441	16.90622507	2.185251075	0.032810164	0.225422468	-4.085861738
PROCR	Q9UNN8	0.329138545	18.12813794	2.181380948	0.033108925	0.225422468	-4.093560179
APOA4	P06727	0.219515534	24.74134814	2.147226326	0.035850679	0.231016858	-4.160997839
IGHA2	P01877	-0.581079185	22.14534218	-2.148308132	0.035760882	0.231016858	-4.158875717
IGKV1D-16	P01601	-0.460737176	17.94824627	-2.144278332	0.036096384	0.231016858	-4.166776143
APOD	P05090	0.169442932	23.06995805	2.057504745	0.044017871	0.264822565	-4.333810135
APOM	O95445	0.164134074	22.71488279	2.050795066	0.044688808	0.264822565	-4.346478503
B2M	P61769	-0.251391455	18.58492562	-2.058443456	0.043924703	0.264822565	-4.332034931
IGHV4-28	A0A0C4DH34	-0.31804414	20.25526142	-2.064083656	0.043368493	0.264822565	-4.321353994
HSPA6	P17066;P48741	0.331297196	16.77292382	2.011768757	0.048769119	0.283747604	-4.419452529
IGLV1-36	A0A0B4J1U3	-0.407060659	19.04655929	-1.970828565	0.053390209	0.30508691	-4.494694222
GC	P02774	-0.126374613	24.02044143	-1.921826666	0.059409537	0.323241145	-4.582971593
IGHV3-35	A0A0C4DH35	-0.234579433	18.99068409	-1.920365238	0.059597586	0.323241145	-4.585574393
SERPINA1	P01009	0.166576174	29.60815106	1.924167765	0.059109347	0.323241145	-4.57879847

ORM2	P19652	-0.181048632	23.34836155	-1.908343937	0.061163724	0.326206529	-4.606918159
IGKV1-13	A0A0B4J2D9;P0DP09	0.555279617	19.13984707	1.899548453	0.062331621	0.326985552	-4.622459664
A2M	P01023	0.194422174	27.25884855	1.853017231	0.068829372	0.354644421	-4.703622896
PPBP	P02775	0.33447304	19.34521146	1.846248021	0.06982062	0.354644421	-4.715281552
FBLN1	P23142	0.178744696	20.21697188	1.828763837	0.072436636	0.358336656	-4.745219122
MASP2	O00187	0.229088586	20.5572083	1.811967466	0.075026737	0.358336656	-4.773739978
SERPINA4	P29622	-0.134430115	21.61614336	-1.818195129	0.074057488	0.358336656	-4.763192544
VTN	P04004	-0.106408719	24.27884024	-1.813628306	0.074767218	0.358336656	-4.770930265
ALDOA	P04075	0.396259897	17.50727763	1.777035242	0.080662257	0.371556849	-4.832302913
ANTXR1	Q9H6X2	0.190319612	16.13033512	1.763295517	0.082973419	0.371556849	-4.855057298
C1R	P00736	0.113128983	21.39380921	1.753354278	0.084679722	0.371556849	-4.871422119
CRTAC1	Q9NQ79	-0.135839468	25.50560307	-1.766340345	0.082456557	0.371556849	-4.850028418
IGLV7-43	P04211	-0.258425438	18.68207859	-1.752882555	0.084761406	0.371556849	-4.872196583
PGLYRP2	Q96PD5	0.134492334	21.98841902	1.753425625	0.084667373	0.371556849	-4.871304968
CETP	P11597	0.188252613	19.64946054	1.74194025	0.086674598	0.374809074	-4.890108782
CD44	P16070	0.138433934	19.53366049	1.673146154	0.099536895	0.424690754	-5.000401127
IGHV1-45	A0A0A0MS14	-0.500790632	18.14166577	-1.66252216	0.101655955	0.428025075	-5.017074819
F13A1	P00488	-0.116686859	20.45630539	-1.644239638	0.105388699	0.432363894	-5.045541991
ITIH1	P19827	-0.112813698	23.75539376	-1.646796377	0.104860077	0.432363894	-5.041578195
APOL1	O14791	-0.168915726	22.23303228	-1.604882411	0.113802849	0.440731604	-5.105849677
AZGP1	P25311	-0.19398149	20.97586889	-1.605109496	0.113752788	0.440731604	-5.10550554
CDH5	P33151	0.127521657	19.45344698	1.586151547	0.117993649	0.440731604	-5.134081994
CLU	P10909	-0.105725545	24.52346611	-1.603733591	0.114056377	0.440731604	-5.107589981
F13B	P05160	-0.127282807	20.09022815	-1.599090801	0.115085623	0.440731604	-5.114611552
HGFAC	Q04756	0.183719938	22.02980832	1.580554147	0.119269814	0.440731604	-5.142459804
IGKV1-37	A0A075B6S9;P0DSN7	-0.335452088	17.84196911	-1.585222872	0.118204616	0.440731604	-5.13547385
IGLV5-45	A0A087WSX0	-0.341492661	17.60193279	-1.58648655	0.11791762	0.440731604	-5.133579723
IGLV7-46	A0A075B6I9	0.240663091	23.43084229	1.578138822	0.119823905	0.440731604	-5.14606651
B4GALT1	P15291	-0.238264835	15.01282912	-1.559214081	0.124237217	0.444482824	-5.17415075
CST3	P01034	-0.15762848	20.15856768	-1.54492496	0.127654884	0.444482824	-5.195149346
F5	P12259	0.128985026	19.73479524	1.52021397	0.133741769	0.444482824	-5.231042986
IGHV2-5	P01817	0.464147112	17.18391003	1.537350892	0.12949662	0.444482824	-5.206207652

IGHV2-70D	A0A0C4DH43	0.715734154	20.76314767	1.551435668	0.126088467	0.444482824	-5.185603583
IGKV3D-15	A0A087WSY6	-0.194959929	23.221914	-1.526952688	0.132059469	0.444482824	-5.221307669
PIGR	P01833	0.203794494	17.18357035	1.553539733	0.125585542	0.444482824	-5.182510783
PROS1	P07225	-0.09656486	22.03519437	-1.505352826	0.137511874	0.444482824	-5.25237208
PVR	P15151	-0.19816458	17.76197872	-1.512513816	0.13568485	0.444482824	-5.242118608
RARRES2	Q99969	-0.23109035	17.17479541	-1.529365834	0.131461138	0.444482824	-5.217811777
THBS4	P35443	-0.218816899	16.30121268	-1.523836321	0.132835359	0.444482824	-5.225814774
VASN	Q6EMK4	0.103796149	18.98211768	1.507871089	0.13686717	0.444482824	-5.248771429
F2	P00734	-0.101099743	22.60895955	-1.484205497	0.143020968	0.457667096	-5.282389081
IGHV3-43	A0A0B4J1X8	0.154155655	20.38559401	1.471483881	0.146417829	0.461032511	-5.30025687
IGHV6-1	A0A0B4J1U7	0.154350007	23.19895361	1.469496673	0.146954113	0.461032511	-5.303035064
IGHD	P01880	-0.385252411	20.37351674	-1.452724851	0.151541835	0.468590773	-5.326343829
IGLV5-37	A0A075B6J1	0.399957307	17.55780818	1.450020276	0.152292001	0.468590773	-5.330079254
CASP14	P31944	0.340880057	16.4559287	1.430891995	0.157680591	0.471852194	-5.356313364
IGHV3OR16-9	A0A0B4J2B5	0.174296904	25.37700451	1.433024266	0.157072675	0.471852194	-5.353405053
MBL2	P11226	0.316151185	19.19842089	1.430559406	0.157775577	0.471852194	-5.356766635
C3	P01024	-0.085076581	27.42067747	-1.419450132	0.160973959	0.472584099	-5.371850554
CD14	P08571	0.149829196	17.49825708	1.423394058	0.159832792	0.472584099	-5.36650813
IGKV1-12	A0A0C4DH73;P01611	-0.28470728	18.83870655	-1.376407581	0.173841714	0.50572135	-5.429255261
IGKV2D-24	A0A075B6R9	0.174326089	22.35820084	1.359001076	0.179263857	0.516796705	-5.451999898
IGKV2D-30	A0A075B6S6	0.13047525	23.81203912	1.351453281	0.181654697	0.518634348	-5.461778015
IGLV3-10	A0A075B6K4	-0.32512183	20.43184816	-1.342452032	0.184537557	0.518634348	-5.473372218
LRP1	Q07954	0.182325295	15.60416187	1.341751129	0.184763486	0.518634348	-5.474271973
ECM1	Q16610	-0.114189303	20.98970774	-1.315320182	0.193437189	0.529058979	-5.507878979
IGHG4	P01861	-0.11265185	29.94185739	-1.323673973	0.190663231	0.529058979	-5.497325113
IGKV6-21	A0A0C4DH24	0.201502229	20.41546094	1.31831932	0.192437819	0.529058979	-5.504097219
F12	P00748	-0.17979134	23.16574553	-1.293486132	0.200830773	0.537730931	-5.535165862
GSN	P06396	-0.097442775	22.72015392	-1.291106762	0.201649099	0.537730931	-5.538113419
PRG4	Q92954	0.164814148	19.99433815	1.292625956	0.201126322	0.537730931	-5.536232036
CTSD	P07339	0.279241195	16.71558484	1.277313418	0.206442149	0.540436084	-5.555099594
IGKV2-24	A0A0C4DH68	0.227044543	18.75845087	1.273647658	0.20773012	0.540436084	-5.559584892
RNASE4	P34096	-0.167574834	16.01393125	-1.279141328	0.205802136	0.540436084	-5.552858474

IGLC3	P0DOY3	-0.123846268	26.89424213	-1.262736703	0.211599051	0.54169357	-5.572863015
IGLV1-51	P01701	-0.116150537	21.48558024	-1.263476253	0.211335137	0.54169357	-5.571966432
CPQ	Q9Y646	0.172078828	15.41285937	1.249466682	0.216376142	0.54952671	-5.588866268
CLEC3B	P05452	-0.128798696	19.6079503	-1.244190109	0.218297621	0.55004125	-5.595185153
A0A0J9YY99	A0A0J9YY99	0.160583867	22.0311856	1.231955197	0.222801309	0.552685418	-5.609739337
PTPRJ	Q12913	-0.140751635	17.27642982	-1.235057944	0.221652781	0.552685418	-5.606061339
C1QC	P02747	0.078662052	24.01422472	1.220057623	0.227245855	0.559374411	-5.623761328
SERPIND1	P05546	-0.113021257	23.243182	-1.19775829	0.235750047	0.571515265	-5.649694039
TFRC	P02786	-0.109608998	18.88003064	-1.199827521	0.234951325	0.571515265	-5.647306803
COMP	P49747	0.121041543	19.06980122	1.188576328	0.239318038	0.571948332	-5.660239789
ORM1	P02763	-0.119573029	25.21210232	-1.18810212	0.239503364	0.571948332	-5.660782332
MAN1A1	P33908	0.082058963	18.17572925	1.176125742	0.244218314	0.578887856	-5.674416162
HRG	P04196	0.095658518	24.02525026	1.14565134	0.256516049	0.603567174	-5.708513638
ICOSLG	O75144	0.122003933	17.06558517	1.14046635	0.25865157	0.604149653	-5.714229954
APOE	P02649	0.101890021	23.94597314	1.119331629	0.267486823	0.61228929	-5.737273794
IGHV3-64	A0A075B6Q5	0.231161671	18.36884995	1.118410766	0.267876564	0.61228929	-5.738268458
IGLV3-19	P01714	-0.245242228	17.43613847	-1.12143075	0.266599896	0.61228929	-5.73500351
IGKV3D-7	A0A0C4DH55	0.302456819	17.74123439	1.110946573	0.27105044	0.615149934	-5.746301926
IGHG3	P01860	-0.109426691	28.86657914	-1.099631884	0.275911813	0.621773101	-5.758381239
C6	P13671	-0.080978419	20.81458037	-1.068224149	0.289724489	0.639190032	-5.791289372
C9	P02748	0.101881865	23.35420404	1.032477416	0.306017554	0.639190032	-5.827627629
F9	P00740	-0.081906415	19.67378242	-1.030510997	0.306931567	0.639190032	-5.82959202
IGF2R	P11717	0.10731931	16.23083578	1.057367804	0.294608172	0.639190032	-5.802451198
IGHV3-38	A0A0C4DH36	0.15789887	18.46590523	1.043951561	0.300721166	0.639190032	-5.816093371
IGHV3-38-3	P0DTE1	0.16355781	18.49955147	1.045451695	0.300033371	0.639190032	-5.814576303
IGLV2-14	P01704	-0.197117835	18.36748517	-1.029053541	0.307610203	0.639190032	-5.831045644
MCAM	P43121	0.093999912	16.95370476	1.039440387	0.302795977	0.639190032	-5.820642829
MMP2	P08253	0.132226464	15.80052955	1.05030833	0.297814037	0.639190032	-5.809650457
NID1	P14543	-0.10872142	16.29431654	-1.058075751	0.294287991	0.639190032	-5.801726673
PLG	P00747	-0.072035632	23.50145023	-1.057429074	0.294580453	0.639190032	-5.802388512
PRSS3	P35030	-0.190441646	18.52058261	-1.060744053	0.293083355	0.639190032	-5.798991695
MASP1	P48740	0.101370691	17.60357112	1.024267677	0.309845805	0.639681661	-5.835804971

IGKC	P01834	-0.100039248	24.59609776	-1.019494624	0.312086356	0.64017714	-5.840530248
ATRN	O75882	0.073292865	19.32218151	1.007795693	0.317624229	0.647386963	-5.852021996
IGHV4-34	P06331	-0.093710552	23.17318374	-0.972279985	0.334838484	0.678153892	-5.886123486
APCS	P02743	-0.158145531	17.29304555	-0.947333265	0.347291966	0.689825453	-5.909368767
APOC2	P02655	0.102794018	22.68758033	0.942673407	0.349651265	0.689825453	-5.913645904
FGA	P02671	0.053898041	26.93221419	0.935919375	0.353089319	0.689825453	-5.919808907
FGB	P02675	0.072809139	25.93340165	0.94346285	0.349250836	0.689825453	-5.912922738
IGKJ1	A0A0A0MT89	0.160535491	18.3672963	0.935762025	0.353169677	0.689825453	-5.919951976
SERPING1	P05155	0.071004047	24.40760348	0.935045903	0.353535545	0.689825453	-5.920602804
CFB	P00751	0.065967018	24.44247713	0.917058587	0.362805798	0.699384671	-5.936791456
HP	P00738	0.125357191	27.90144854	0.917635648	0.362505991	0.699384671	-5.93627684
ANGPTL6	Q8NI99	0.132292463	15.76291665	0.896630416	0.373521467	0.705729683	-5.954806339
HSP90B1	P14625	0.120762205	16.54770633	0.89593737	0.373888496	0.705729683	-5.955410592
IGHV5-51	A0A0C4DH38	-0.144524909	20.48546988	-0.889845913	0.377124299	0.705729683	-5.960702054
IL1RAP	Q9NPH3	0.137785704	18.0207882	0.890403398	0.376827427	0.705729683	-5.960219244
ITIH2	P19823	-0.06001589	24.11205682	-0.903492596	0.369899684	0.705729683	-5.948798824
C1QB	P02746	0.079553362	22.22239445	0.87746857	0.383753523	0.709833106	-5.971345648
IGLV3-27	P01718	-0.128086525	20.30658366	-0.878574442	0.383158266	0.709833106	-5.970400585
Biognosys	Biognosys	0.095441112	25.62414704	0.846713995	0.400539597	0.712070394	-5.997163345
DBH	P09172	-0.17022439	18.18887917	-0.869720318	0.387940433	0.712070394	-5.977934682
FCGBP	Q9Y6R7	0.104384182	18.88481904	0.846867261	0.400454836	0.712070394	-5.997036908
FCN2	Q15485	-0.146022575	17.98903156	-0.84809679	0.399775274	0.712070394	-5.996021804
HPR	P00739	-0.108780949	21.92943112	-0.862652841	0.391784262	0.712070394	-5.983895139
HPX	P02790	-0.065584791	24.47062662	-0.851853392	0.397703402	0.712070394	-5.992911439
SERPINC1	P01008	-0.042041153	24.69917531	-0.863967543	0.391067439	0.712070394	-5.982789955
CP	P00450	0.106986223	24.83966761	0.833386755	0.407952128	0.713440324	-6.008072211
IGKV1-33	P01593;P01594	-0.134766369	20.72783198	-0.833303518	0.407998685	0.713440324	-6.008139812
PLXDC2	Q6UX71	0.091235031	16.9524985	0.83400955	0.407603877	0.713440324	-6.007566191
ALCAM	Q13740	0.11558978	15.11098929	0.802017825	0.425727058	0.740394884	-6.033081553
C4BPA	P04003	0.050825529	22.57264048	0.780207385	0.438354592	0.754158438	-6.049917152
IGLV2-11	P01706	-0.087529522	22.46425431	-0.781945145	0.437340459	0.754158438	-6.048592423
HSPA5	P11021	0.076940798	17.49816079	0.765716788	0.446864919	0.756596689	-6.060851217

© Thieme. All rights reserved.

IGHV1-46	P01743	-0.107252917	19.41932765	-0.766966073	0.446127438	0.756596689	-6.059916463
IGKV1-8	A0A0C4DH67;A0A0C4DH69	-0.134490223	20.51793685	-0.765775817	0.446830057	0.756596689	-6.060807083
CD5L	O43866	-0.184874678	22.14725388	-0.757402179	0.451791303	0.760831533	-6.067034413
CFH	P08603	-0.050241575	23.56773017	-0.753487968	0.454121321	0.760831533	-6.069922322
IGKV1-17	P01599	0.084710066	21.68553866	0.748001962	0.457398647	0.762331079	-6.073945209
IGHV3-72	A0A0B4J1Y9	0.109130921	16.81740952	0.705864348	0.483020891	0.794708014	-6.103882453
LRG1	P02750	0.089874167	22.76076104	0.710743898	0.480013472	0.794708014	-6.10050298
SAA2	P0DJI9	-0.339706318	20.14948108	-0.703834238	0.484275196	0.794708014	-6.105281724
PRR4	Q16378	0.140918561	16.25526049	0.697462983	0.488223412	0.797099448	-6.109647434
IGHV3OR16-12	A0A075B7B8	0.104190849	22.98324907	0.687942848	0.494155987	0.802689928	-6.116098065
CFI	P05156	-0.049281668	22.28700521	-0.667137938	0.507257276	0.818100509	-6.12989133
SERPINF1	P36955	-0.051339498	21.21337548	-0.664776193	0.508756254	0.818100509	-6.131430767
IGLV2-8	P01709	0.133337273	19.80012275	0.651500101	0.517226579	0.827562527	-6.139984326
IGHV2-26	A0A0B4J1V2	-0.125711143	20.85205353	-0.6438034	0.52217125	0.831317414	-6.144865316
C1S	P09871	0.032932269	23.67880495	0.621770134	0.53646285	0.848819695	-6.158521804
PTGDS	P41222	-0.080684009	18.18118388	-0.61870025	0.538469994	0.848819695	-6.160387321
AHSG	P02765	-0.045077666	25.32166584	-0.59023401	0.557263804	0.849703273	-6.177251536
AMBP	P02760	0.039053685	23.06370361	0.594977078	0.554109749	0.849703273	-6.174496066
APOC4	P55056	0.069890631	19.06183342	0.599402083	0.551175299	0.849703273	-6.17190573
CFHR5	Q9BXR6	0.058850818	19.14777894	0.602293057	0.549262386	0.849703273	-6.170203157
F10	P00742	-0.038919545	20.68242872	-0.589702551	0.557617773	0.849703273	-6.177558928
IGHG1	P01857	-0.050852541	28.7073876	-0.594454677	0.554456697	0.849703273	-6.174800621
IGKV3-20	P01619	0.096709041	24.68348456	0.608037124	0.545471613	0.849703273	-6.166796316
C8A	P07357	0.04356329	21.57901375	0.577958539	0.565468161	0.857582045	-6.184281737
IGLV1-47	P01700	-0.064274779	23.39176021	-0.562660555	0.575775201	0.869094642	-6.192838482
IGHV1-69	P01742	-0.126579497	17.12236975	-0.548652504	0.585292228	0.875203332	-6.200474572
KNG1	P01042	-0.035178965	25.13774969	-0.552366928	0.582761369	0.875203332	-6.198468317
C8B	P07358	0.041498516	20.04951715	0.488179503	0.627212895	0.882371453	-6.231251057
CDH13	P55290	0.047855733	17.38007911	0.508544797	0.612947546	0.882371453	-6.221283858
CPN1	P15169	-0.043712269	20.45655022	-0.470492715	0.639719303	0.882371453	-6.239579537
EFEMP1	Q12805	-0.039590827	18.45932662	-0.509730462	0.612121561	0.882371453	-6.220691131
FCGR3A	P08637	-0.058863797	18.02051196	-0.487330674	0.627810642	0.882371453	-6.231657724

FCN3	O75636	0.061666403	20.95291333	0.484244435	0.629986087	0.882371453	-6.233130398
IGHV1-3	A0A0C4DH29	-0.092392409	18.62718428	-0.471421111	0.639060171	0.882371453	-6.23914995
IGHV3-49	A0A0A0MS15	-0.051799255	24.21106798	-0.533027906	0.59599511	0.882371453	-6.20876708
IGHV3-7	P01780	0.047941231	23.95792073	0.536826794	0.593384459	0.882371453	-6.206772703
IGKV1-27	A0A075B6S5	-0.073184837	20.53661428	-0.491736547	0.624710749	0.882371453	-6.229539276
IGKV3-7	A0A075B6H7	-0.088797802	25.22219247	-0.492485175	0.624184701	0.882371453	-6.229177439
IGKV4-1	P06312	-0.093656044	22.65869624	-0.518347659	0.60613368	0.882371453	-6.216342209
IGLL1	P15814	-0.111979924	17.40136498	-0.527341954	0.599912578	0.882371453	-6.211725939
JCHAIN	P01591	0.076583892	24.15589702	0.519457427	0.605364485	0.882371453	-6.215776881
LYVE1	Q9Y5Y7	0.063025649	19.10432257	0.475051603	0.636485444	0.882371453	-6.237461982
SELENOP	P49908	-0.048010688	20.09414858	-0.510264617	0.61174961	0.882371453	-6.220423655
SERPINA3	P01011	-0.031275551	24.93513849	-0.471473733	0.63902282	0.882371453	-6.239125576
SERPINA5	P05154	-0.037975823	20.68735436	-0.490891275	0.625304942	0.882371453	-6.229947169
PON3	Q15166	0.039696437	18.90494071	0.464879922	0.64371041	0.8840658	-6.242158786
APOA2	P02652	0.043505011	26.60555076	0.448173099	0.655652317	0.896618554	-6.249654208
APOB	P04114	0.040385743	24.44720652	0.41852234	0.677068677	0.901961195	-6.262285903
CFHR1	Q03591	0.068385697	19.01839477	0.396427412	0.693204714	0.901961195	-6.271140038
FBLN5	Q9UBX5	-0.046554531	16.03194504	-0.397790972	0.692204663	0.901961195	-6.27060744
IGHV1-18	A0A0C4DH31	-0.056230257	19.70910175	-0.41649825	0.678540713	0.901961195	-6.263116879
IGHV3-13	P01766	-0.046824706	19.6806276	-0.399881851	0.690672255	0.901961195	-6.269787221
IGHV3-64D	A0A0J9YX35	0.065806729	21.88556493	0.427842713	0.670306738	0.901961195	-6.258407822
IGHV3-9	P01782	-0.059852643	18.37710943	-0.398500373	0.691684597	0.901961195	-6.270329632
LCAT	P04180	-0.028190262	20.12150395	-0.428505688	0.669826782	0.901961195	-6.258128733
MST1	P26927	-0.051911795	19.8041412	-0.405488709	0.68656941	0.901961195	-6.267566634
NRP1	O14786	-0.040773169	16.92599587	-0.423979953	0.673105891	0.901961195	-6.260025364
PROZ	P22891	-0.062838941	19.99263516	-0.39618485	0.693382669	0.901961195	-6.271234591
SERPINA6	P08185	-0.037932743	22.11679874	-0.433918475	0.665913423	0.901961195	-6.255834083
C4A	P0C0L4	-0.065869568	22.76171753	-0.38738979	0.699846802	0.906684116	-6.274624098
IGLC7	A0M8Q6	-0.046205744	23.59452166	-0.373441429	0.710144183	0.912635095	-6.279844384
IGLL5	B9A064	-0.059443879	25.33166588	-0.37474109	0.709182377	0.912635095	-6.279366022
SPP2	Q13103	0.056116357	18.46062205	0.366578455	0.715230908	0.915495562	-6.282342987
IGKV1-5	P01602	0.056365712	23.99568337	0.355050233	0.723804589	0.919116938	-6.286436232

© Thieme. All rights reserved.

SERPINA10	Q9UK55	-0.029486257	19.12794806	-0.356677227	0.722592388	0.919116938	-6.285866438
A1BG	P04217	-0.018125583	24.71636092	-0.295379475	0.768729996	0.924787965	-6.305540483
COL6A1	P12109	0.044801401	16.90469092	0.326839147	0.744933948	0.924787965	-6.295903584
GP1BA	P07359	-0.034431887	18.81485698	-0.309268333	0.758195389	0.924787965	-6.301405717
IGHV3-15	A0A0B4J1V0	-0.037278628	23.19524319	-0.297085723	0.767433412	0.924787965	-6.305042729
IGHV3-74	A0A0B4J1X5	0.042405163	21.74129324	0.340074033	0.734995706	0.924787965	-6.29155925
IGKV1-6	A0A0C4DH72	0.046818541	20.82163504	0.304547033	0.761771415	0.924787965	-6.302832509
IGKV3-11	A0A0A0MRZ8;P04433	-0.033730835	23.62078067	-0.336526826	0.73765497	0.924787965	-6.292740464
IGKV3D-20	A0A0C4DH25	0.040174465	22.29827948	0.307452705	0.759569967	0.924787965	-6.301956994
IGLV5-39	A0A0G2JS06	-0.055429365	19.82390901	-0.301676128	0.763948466	0.924787965	-6.303689409
LBP	P18428	0.042578466	19.6026945	0.324597734	0.746621426	0.924787965	-6.296622316
PI16	Q6UXB8	-0.040838009	18.44261066	-0.343318622	0.732566156	0.924787965	-6.290468004
PON1	P27169	-0.032557762	21.86858696	-0.320621932	0.749617725	0.924787965	-6.297885072
QSOX1	O00391	0.025291558	18.03837942	0.313481925	0.755008385	0.924787965	-6.300113882
SERPINF2	P08697	0.017885358	21.74564923	0.297534588	0.767092428	0.924787965	-6.30491131
BCHE	P06276	-0.023496747	19.57918821	-0.269241969	0.788672847	0.934723374	-6.312808069
C5	P01031	0.017599233	22.35746398	0.27100548	0.787322649	0.934723374	-6.312338833
SELL	P14151	-0.028246276	19.11407196	-0.28057734	0.780005673	0.934723374	-6.309738649
TNXB	P22105	0.031044195	18.04185593	0.271938201	0.786608795	0.934723374	-6.312089419
ITIH4	Q14624	0.014963304	25.40824871	0.265353662	0.791652148	0.934792204	-6.313831875
IGKV2-40	A0A087WW87;P01614	-0.04531879	17.80724058	-0.239721273	0.811368387	0.954551044	-6.320209118
F7	P08709	-0.026219604	18.14254465	-0.227147331	0.821086068	0.959085296	-6.323101293
SHBG	P04278	0.043568043	19.13419676	0.226978447	0.821216784	0.959085296	-6.323139081
APOA1	P02647	0.016666761	28.17937677	0.199721892	0.842377553	0.959291163	-6.328869925
C4B_2	P0C0L5	0.022227263	25.14358861	0.202242901	0.840415194	0.959291163	-6.32837055
C7	P10643	-0.015770118	20.98655774	-0.20644425	0.83714712	0.959291163	-6.327524426
GPX3	P22352	-0.020563598	20.17516473	-0.216606943	0.829253949	0.959291163	-6.325405902
IGHA1	P01876	0.036585881	28.32240787	0.213118796	0.831961179	0.959291163	-6.326144499
IGHG2	P01859	0.027136623	28.28187458	0.222750896	0.824490559	0.959291163	-6.32407584
IGLV2-18	A0A075B6J9	-0.042360423	18.18152469	-0.20604344	0.837458771	0.959291163	-6.327605896
DSG1	Q02413	-0.044215664	17.42526692	-0.19222873	0.848216126	0.961891227	-6.330317283
IGHV1OR15-1	A0A075B7D0	-0.053565027	22.64387828	-0.184534115	0.854220565	0.961891227	-6.331746032

IGLV3-21	P80748	-0.036577829	19.63271675	-0.187290848	0.852068353	0.961891227	-6.331240858
VWF	P04275	0.027937164	20.03765811	0.18138001	0.856684374	0.961891227	-6.332314848
COLEC11	Q9BWP8	-0.020469362	16.55916356	-0.168299693	0.866917003	0.969855747	-6.334569225
IGKV6D-21	A0A0A0MT36	-0.046133519	19.45924563	-0.164569508	0.869839373	0.969855747	-6.335181242
A0A0G2JRQ6	A0A0G2JRQ6	-0.016860784	22.48027177	-0.084535934	0.932913485	0.970919159	-6.345009905
APOC3	P02656	-0.011162244	24.93394732	-0.095862911	0.923950863	0.970919159	-6.344002385
APOH	P02749	0.016964526	23.13528578	0.143953005	0.88602293	0.970919159	-6.338316621
ART3	Q13508	-0.011919947	15.57394202	-0.092281484	0.926783701	0.970919159	-6.344334622
CFHR4	Q92496	-0.016980344	19.36190214	-0.096588915	0.923376728	0.970919159	-6.343933494
CHL1	O00533	-0.00909878	18.65286105	-0.094851218	0.924750994	0.970919159	-6.344097518
CPN2	P22792	0.009173021	21.64547424	0.104628357	0.917021814	0.970919159	-6.343135899
DCD	P81605	0.022694746	17.94749331	0.104427265	0.917180707	0.970919159	-6.343156626
FGG	P02679	-0.006620449	27.31923226	-0.121397189	0.903784567	0.970919159	-6.341267234
GLIPR2	Q9H4G4	0.01020722	17.27340872	0.121539092	0.903672662	0.970919159	-6.341250239
IGHM	P01871	-0.014815093	25.77090078	-0.085104138	0.932463668	0.970919159	-6.344962378
IGHV1-24	A0A0C4DH33	-0.034183784	18.62555183	-0.144410096	0.885663567	0.970919159	-6.338251644
IGHV4-4	A0A075B6R2	-0.015665505	17.95232739	-0.115636082	0.908329464	0.970919159	-6.341940486
IGKV1-16	P04430	-0.020993883	19.2186933	-0.140328953	0.888872988	0.970919159	-6.338824499
IGKV1-39	P01597;P04432	0.017217313	21.20601136	0.101486795	0.919504492	0.970919159	-6.343455158
IGKV2-30	P06310	0.019720466	18.14253546	0.114076786	0.909560118	0.970919159	-6.342117082
IGLV9-49	A0A0B4J1Y8	0.021187843	19.53944873	0.094958713	0.924665975	0.970919159	-6.344087457
KLKB1	P03952	-0.007327701	22.83034345	-0.102458829	0.918736237	0.970919159	-6.343357415
LCP1	P13796	-0.015277512	17.5959815	-0.119127475	0.905574752	0.970919159	-6.341536381
PLTP	P55058	-0.007140722	19.29016912	-0.082519844	0.934509691	0.970919159	-6.345175973
PROC	P04070	-0.011677265	19.91220999	-0.112143895	0.911085935	0.970919159	-6.342332664
C8G	P07360	-0.005524818	20.92334343	-0.059999288	0.952356816	0.986259486	-6.346758608
BTD	P43251	-0.00413907	17.97287886	-0.045047521	0.964219935	0.994581367	-6.347533108
C4BPB	P20851	-0.003428229	20.55501721	-0.041925148	0.966698422	0.994581367	-6.347667021
CPB2	Q96IY4	-0.002691026	20.191204	-0.032983838	0.973797624	0.994581367	-6.347997308
CRISP3	P54108	0.004277249	15.97531924	0.030294972	0.975932966	0.994581367	-6.348081213
IGLV8-61	A0A075B6I0	0.006047901	22.39312086	0.037729332	0.970029506	0.994581367	-6.34783183
IGLV3-9	A0A075B6K5	-0.004105076	20.25204862	-0.026127032	0.979243248	0.994786792	-6.348197179

C1RL	Q9NZP8	-0.001226475	18.68933412	-0.014661873	0.988350879	0.995870061	-6.348427793
LPA	P08519	-0.005037311	21.69724285	-0.013031861	0.989645873	0.995870061	-6.348450054
SERPINA7	P05543	-0.001312852	20.80047764	-0.016912298	0.986563037	0.995870061	-6.348392753
IGLV1-40	P01703	0.000518633	20.29165144	0.003970639	0.996845149	0.999970056	-6.348526028
ALB	P02768	0	32.0336323	0	1	1	-6.348533803

© Thieme. All rights reserved.

Table S2. Comparison of plasma protein abundance between type 1 diabetes with IR group versus type 1 diabetes without IR group. Differential protein abundance was assessed using linear regression models (protein ~ group), with the pairwise contrast IR+ vs IR−. P-values were adjusted for multiple testing using the Benjamini–Hochberg false discovery rate (FDR), with significance defined as p <0.05.

Protein (gene name)	UniProtID	logFC	AveExpr	t	P.Value	adj.P.Val	B
ABCB9	Q9NP78	-1.32030746	19.7562156	-4.14186942	0.00011013	0.01762157	1.14151937
VWF	P04275	0.73736175	20.0376581	4.14589549	0.00010864	0.01762157	1.15382475
CAMP	P49913	-0.97632124	18.6276485	-3.55268568	0.00075256	0.08027264	-0.58555189
PPBP	P02775	0.70367316	19.3452115	3.36380293	0.0013481	0.10784833	-1.10467523
IGLV8-61	A0A075B6I0	-0.58415686	22.3931209	-3.15598246	0.00250775	0.16049585	-1.65391167
CFH	P08603	0.23289197	23.5677302	3.02481081	0.00366688	0.19556675	-1.98800036
PROS1	P07225	0.21963193	22.0351944	2.96514067	0.00434506	0.19863147	-2.13661236
CFB	P00751	0.23737751	24.4424771	2.85785661	0.00586542	0.2085484	-2.39834689
IGHV6-1	A0A0B4J1U7	-0.34820743	23.1989536	-2.870983	0.00565602	0.2085484	-2.36670618
C4B_2	P0C0L5	0.3452718	25.1435886	2.7206899	0.00852428	0.22011032	-2.72243613
CASP14	P31944	-0.73585666	16.4559287	-2.67503285	0.00962983	0.22011032	-2.82760993
FGG	P02679	0.17405468	27.3192323	2.76399676	0.00758437	0.22011032	-2.6214177
IGKV2D-24	A0A075B6R9	-0.41245841	22.3582008	-2.78463454	0.00717084	0.22011032	-2.57285111
IGLV1-51	P01701	-0.2853532	21.4855802	-2.68818555	0.00929861	0.22011032	-2.79745253
IGLV1-40	P01703	-0.39901301	20.2916514	-2.6455641	0.01041142	0.2221102	-2.8947617
CETP	P11597	-0.32630906	19.6494605	-2.61488142	0.01128631	0.22572624	-2.96406406
IGHV3-64D	A0A0J9YX35	-0.44413416	21.8855649	-2.50068302	0.01516176	0.28539783	-3.21639338
C5	P01031	0.18115228	22.357464	2.41578775	0.01878186	0.31798417	-3.3981274
PLTP	P55058	-0.23911565	19.2901691	-2.39306723	0.01987401	0.31798417	-3.44590245
SHBG	P04278	-0.53075451	19.1341968	-2.39464364	0.01979644	0.31798417	-3.44259956
IGHV3-15	A0A0B4J1V0	-0.34221642	23.1952432	-2.36185558	0.02146693	0.32711505	-3.51093129
BTD	P43251	0.24674827	17.9728789	2.32569584	0.0234541	0.34115053	-3.58539344
FGA	P02671	0.15077085	26.9322142	2.26732339	0.02700857	0.37577143	-3.70359258
C1RL	Q9NZP8	0.21649336	18.6893341	2.24133086	0.02873928	0.38319036	-3.75542081
C1S	P09871	0.13373038	23.678805	2.18659781	0.03270676	0.38691895	-3.86291797
CPB2	Q96IY4	0.19662133	20.191204	2.08710609	0.04116095	0.38691895	-4.0525521

GC	P02774	0.15765846	24.0204414	2.07635823	0.04217908	0.38691895	-4.07258672
IGHV1-2	P23083	-0.2956686	18.375055	-2.08346399	0.04150355	0.38691895	-4.0593511
IGHV2-5	P01817	0.72772503	17.18391	2.08744622	0.04112908	0.38691895	-4.05191663
IGHV3-13	P01766	-0.28054924	19.6806276	-2.07489613	0.04231926	0.38691895	-4.0753053
IGKC	P01834	-0.23712918	24.5960978	-2.09281168	0.04062919	0.38691895	-4.04188057
IGLV7-46	A0A075B6I9	-0.36920531	23.4308423	-2.09669029	0.04027112	0.38691895	-4.03461193
LBP	P18428	0.33182112	19.6026945	2.19073641	0.03239077	0.38691895	-3.85486778
SERPINA5	P05154	0.18797254	20.6873544	2.10427826	0.03957854	0.38691895	-4.02035857
VASN	Q6EMK4	0.17118819	18.9821177	2.15371089	0.03531535	0.38691895	-3.92643061
CNDP1	Q96KN2	0.31964777	19.3016768	2.04940457	0.04482895	0.38954543	-4.12243901
IGHV3-7	P01780	-0.20989436	23.9579207	-2.03543115	0.04625852	0.38954543	-4.14806286
MASP1	P48740	0.2331382	17.6035711	2.0400703	0.0457796	0.38954543	-4.13957254
MASP2	O00187	0.29301999	20.5572083	2.0071269	0.04927515	0.40430896	-4.19950171
C4BPA	P04003	0.14983722	22.5726405	1.99195025	0.05096129	0.4076903	-4.226826
BCHE	P06276	0.18980287	19.5791882	1.88351214	0.06450968	0.43248295	-4.41678291
C3	P01024	0.12893464	27.4206775	1.86298926	0.06739073	0.43248295	-4.45168183
IGHV2-70D	A0A0C4DH43	1.02663758	20.7631477	1.92721346	0.05872074	0.43248295	-4.34134875
IGHV3-9	P01782	-0.33105473	18.3771094	-1.9088677	0.06109477	0.43248295	-4.37320108
IGHV4-28	A0A0C4DH34	-0.34007196	20.2552614	-1.911355	0.0607682	0.43248295	-4.36889824
IGKV4-1	P06312	-0.3955632	22.6586962	-1.89597168	0.06281194	0.43248295	-4.39543121
IGLC3	P0DOY3	-0.21699783	26.8942421	-1.91609002	0.06015061	0.43248295	-4.36069344
KLKB1	P03952	0.15374363	22.8303435	1.86169882	0.06757546	0.43248295	-4.45386492
MST1	P26927	-0.27616938	19.8041412	-1.86818064	0.06665188	0.43248295	-4.44288585
NID1	P14543	0.22345395	16.2943165	1.88330345	0.06453844	0.43248295	-4.41713948
C4A	P0C0L4	0.36017064	22.7617175	1.83443485	0.07157926	0.44067911	-4.49967533
IGKV3D-15	A0A087WSY6	-0.27039264	23.221914	-1.83402699	0.07164063	0.44067911	-4.50035609
PRG4	Q92954	0.26871354	19.9943382	1.82515004	0.07298748	0.44067911	-4.51513932
IGHV3-38	A0A0C4DH36	-0.31402091	18.4659052	-1.79800345	0.07723869	0.45423957	-4.55995194
IGLV7-43	P04211	-0.30520394	18.6820786	-1.79282674	0.07807243	0.45423957	-4.56842955
ALDOA	P04075	-0.44618605	17.5072776	-1.73285626	0.08828991	0.46127145	-4.66504439
B4GALT1	P15291	0.29568661	15.0128291	1.67574561	0.09902395	0.46127145	-4.75430104
C4BPB	P20851	0.16626359	20.5550172	1.76089149	0.0833834	0.46127145	-4.62024494

© Thieme. All rights reserved.

C6	P13671	0.14476644	20.8145804	1.65383293	0.10341636	0.46127145	-4.78782938
CFI	P05156	0.14725108	22.2870052	1.72631233	0.0894689	0.46127145	-4.67540841
F10	P00742	0.1290602	20.6824287	1.69351174	0.09557582	0.46127145	-4.72682414
F13B	P05160	0.15382323	20.0902281	1.67361645	0.09944393	0.46127145	-4.75757638
IGHV3-38-3	P0DTE1	-0.29843714	18.4995515	-1.65202338	0.10378608	0.46127145	-4.79058026
IGHV3OR16-9	A0A0B4J2B5	-0.23950735	25.3770045	-1.70534933	0.09333351	0.46127145	-4.708371
IGLV5-39	A0A0G2JS06	-0.35332749	19.823909	-1.66536375	0.1010856	0.46127145	-4.77023606
MAN1A1	P33908	0.13940565	18.1757292	1.73036899	0.08873651	0.46127145	-4.66898779
MCAM	P43121	-0.17430203	16.9537048	-1.66918782	0.10032216	0.46127145	-4.76437697
PIP	P12273	0.32352876	16.9164681	1.73165377	0.08850559	0.46127145	-4.6669515
SERPINA1	P01009	0.17311864	29.6081511	1.73182704	0.08847448	0.46127145	-4.66667677
SERPINA10	Q9UK55	0.15973504	19.1279481	1.67334916	0.09949676	0.46127145	-4.75798729
SERPINF1	P36955	0.14894746	21.2133755	1.67027345	0.10010629	0.46127145	-4.76271138
SERPING1	P05155	0.14499763	24.4076035	1.65364148	0.10345542	0.46127145	-4.78812055
IGKV1-27	A0A075B6S5	-0.2821364	20.5366143	-1.6417277	0.10591017	0.46426375	-4.80617971
AFM	P43652	0.15899812	22.5366781	1.62288522	0.10988901	0.46913072	-4.83449907
CPN2	P22792	0.16426342	21.6454742	1.62258901	0.10995251	0.46913072	-4.83494189
PRDX2	P32119	0.39092519	17.3494978	1.61371327	0.11186916	0.47102806	-4.84817635
F9	P00740	0.14294923	19.6737824	1.55756851	0.12462704	0.51129041	-4.93035473
IGHV3-35	A0A0C4DH35	-0.22010557	18.9906841	-1.5604705	0.12394024	0.51129041	-4.92617244
AMBP	P02760	0.11718887	23.0637036	1.54616271	0.1273559	0.51587201	-4.94672325
CFHR4	Q92496	0.30714035	19.3619021	1.51303269	0.13555322	0.52261481	-4.99364059
HPR	P00739	0.22100539	21.9294311	1.51780755	0.13434663	0.52261481	-4.98693637
IGFALS	P35858	0.16885009	21.2678023	1.5136249	0.1354031	0.52261481	-4.99281015
IGKJ1	A0A0A0MT89	-0.30061864	18.3672963	-1.51754259	0.13441336	0.52261481	-4.98730889
FN1	P02751	0.22307889	23.3131335	1.49342385	0.1405985	0.52931201	-5.02096831
PI16	Q6UXB8	-0.20564916	18.4426107	-1.49723635	0.13960613	0.52931201	-5.01568083
IGHV5-51	A0A0C4DH38	-0.27663948	20.4854699	-1.47508447	0.14545006	0.54120952	-5.04622864
APOC4	P55056	0.19543302	19.0618334	1.4515362	0.15187117	0.55858442	-5.07823955
TTR	P02766	0.15094884	25.6064184	1.44529101	0.15361071	0.55858442	-5.08664891
A2M	P01023	-0.1743182	27.2588485	-1.43882184	0.15542898	0.55871149	-5.09532436
APOF	Q13790	-0.17495575	19.9176059	-1.41975142	0.16088656	0.55871149	-5.12068802

AZGP1	P25311	0.1978945	20.9758689	1.41810608	0.1613643	0.55871149	-5.12286156
CRISP3	P54108	0.22965537	15.9753192	1.40868313	0.1641215	0.55871149	-5.13526432
IGKV2-40	A0A087WW87;P01614	0.3123816	17.8072406	1.4310155	0.15764533	0.55871149	-5.10574483
IGKV3-7	A0A075B6H7	-0.29356226	25.2221925	-1.4100087	0.16373145	0.55871149	-5.13352422
F11	P03951	0.14513487	20.0989789	1.36339605	0.1778828	0.56140725	-5.19379496
IGKV1-12	A0A0C4DH73;P01611	-0.33312925	18.8387065	-1.39473558	0.16826924	0.56140725	-5.15348095
IGKV1-13	A0A0B4J2D9;P0DP09	-0.46109675	19.1398471	-1.36603346	0.17705794	0.56140725	-5.19043532
IGKV1-8	A0A0C4DH67;A0A0C4DH69	-0.27536527	20.5179369	-1.35784666	0.17962797	0.56140725	-5.20084414
IGLV2-14	P01704	-0.29958489	18.3674852	-1.35444877	0.18070296	0.56140725	-5.20514706
IGLV5-37	A0A075B6J1	-0.4381086	17.5578082	-1.37553881	0.17410933	0.56140725	-5.17827662
LRP1	Q07954	0.21857791	15.6041619	1.3930347	0.16878052	0.56140725	-5.15569086
QSOX1	O00391	0.12849313	18.0383794	1.37926417	0.17296401	0.56140725	-5.17348984
IGKV1-37	A0A075B6S9;P0DSN7	-0.3285815	17.8419691	-1.34472528	0.18380624	0.56419212	-5.21740454
PLXDC2	Q6UX71	0.16934356	16.9524985	1.34062929	0.18512554	0.56419212	-5.22254318
C8A	P07357	0.1124266	21.5790137	1.2917418	0.20143045	0.60809192	-5.28273706
C1R	P00736	0.09229646	21.3938092	1.23882936	0.2202626	0.62990747	-5.3455087
FGB	P02675	0.11027862	25.9334017	1.23754466	0.22073543	0.62990747	-5.3470019
HABP2	Q14520	0.12734581	18.8936584	1.24028687	0.21972707	0.62990747	-5.34381287
IGF2R	P11717	-0.14816349	16.2308358	-1.26421238	0.21107269	0.62990747	-5.31570562
IGHV3OR16-12	A0A075B7B8	-0.21950183	22.9832491	-1.25513827	0.21432477	0.62990747	-5.32642551
IGLC7	A0M8Q6	-0.17895356	23.5945217	-1.25255702	0.2152566	0.62990747	-5.32946159
IGLV2-11	P01706	-0.15936384	22.4642543	-1.2329406	0.22243607	0.62990747	-5.3523411
APOC2	P02655	0.15040199	22.6875803	1.19447655	0.23702083	0.63088689	-5.39620879
APOL1	O14791	0.14461233	22.2330323	1.18989625	0.23880275	0.63088689	-5.40134455
B2M	P61769	0.16576832	18.5849256	1.1754945	0.24446867	0.63088689	-5.41737044
C2	P06681	0.10006803	20.9655057	1.19742033	0.23588069	0.63088689	-5.39289812
HGFAC	Q04756	-0.15906531	22.0298083	-1.18511142	0.24067455	0.63088689	-5.40668957
IGHG2	P01859	-0.16580169	28.2818746	-1.17864566	0.24322074	0.63088689	-5.41387978
IGHG4	P01861	-0.11861456	29.9418574	-1.20701132	0.23219364	0.63088689	-5.38205806
IGKV1D-16	P01601	-0.30012038	17.9482463	-1.20963411	0.2311927	0.63088689	-5.3790794
LYZ	P61626	0.14790386	20.014237	1.20482844	0.23302909	0.63088689	-5.38453245
ORM2	P19652	0.12916396	23.3483615	1.17905313	0.24305971	0.63088689	-5.41342776

SERPINA6	P08185	0.11965033	22.1167987	1.18532771	0.24058972	0.63088689	-5.40644841
DBH	P09172	0.26126869	18.1888792	1.1560479	0.25227195	0.63671872	-5.43871507
IGHV3-43	A0A0B4J1X8	-0.13980544	20.385594	-1.155715	0.25240707	0.63671872	-5.43907751
IGKV1-17	P01599	-0.15100442	21.6855387	-1.15475012	0.25279897	0.63671872	-5.44012743
SERPINA7	P05543	0.10309179	20.8004776	1.15011551	0.25468749	0.63671872	-5.44515888
LRG1	P02750	0.16668024	22.760761	1.14154493	0.2582063	0.63899259	-5.45441244
ORM1	P02763	0.13227134	25.2121023	1.1381957	0.25959074	0.63899259	-5.4580106
IGKV6-21	A0A0C4DH24	-0.19950981	20.4154609	-1.13040914	0.26282974	0.64202685	-5.46633685
ANTXR1	Q9H6X2	0.13633476	16.1303351	1.09390292	0.27839639	0.64817891	-5.50464393
APOC1	P02654	-0.13694662	22.2524066	-1.08721437	0.28131683	0.64817891	-5.51153184
CLU	P10909	0.08200004	24.5234661	1.07720165	0.28572843	0.64817891	-5.52176724
FCN3	O75636	0.163425	20.9529133	1.11138657	0.27086262	0.64817891	-5.48644799
IGHD	P01880	-0.33003691	20.3735167	-1.07778253	0.28547119	0.64817891	-5.52117593
IGKV1-6	A0A0C4DH72	-0.19045847	20.821635	-1.07292022	0.28762939	0.64817891	-5.52611615
LGALS3BP	Q08380	0.16762955	19.3737818	1.08249257	0.28339132	0.64817891	-5.51636997
SELENOP	P49908	0.12021266	20.0941486	1.10646672	0.27296797	0.64817891	-5.49159622
SERPIND1	P05546	0.12105861	23.243182	1.11105448	0.27100437	0.64817891	-5.48679619
TGFBI	Q15582	0.11092005	18.807326	1.07739775	0.28564157	0.64817891	-5.52156765
VTN	P04004	0.07336053	24.2788402	1.08283963	0.28323848	0.64817891	-5.51601505
VCAM1	P19320	0.11805183	18.727681	1.05750007	0.29454833	0.65912914	-5.54164148
IGHV3-72	A0A0B4J1Y9	-0.18767426	16.8174095	-1.05125671	0.29738197	0.66084883	-5.54786599
APOA2	P02652	0.11711219	26.6055508	1.04481457	0.30032535	0.66278699	-5.55425151
IGLV9-49	A0A0B4J1Y8	0.26614764	19.5394487	1.03300238	0.30577386	0.67018927	-5.56586175
LTF	P02788	-0.16406944	17.5690368	-1.02726228	0.30844566	0.67144634	-5.57145782
F13A1	P00488	0.08213808	20.4563054	1.00234754	0.3202256	0.6773938	-5.59539894
IGKV2-24	A0A0C4DH68	-0.20566437	18.7584509	-0.99914363	0.32176206	0.6773938	-5.5984365
IGLL1	P15814	0.24697946	17.401365	1.00726474	0.31787712	0.6773938	-5.5907188
MBL2	P11226	0.25873825	19.1984209	1.01391689	0.31471848	0.6773938	-5.58435219
SERPINF2	P08697	0.06999717	21.7456492	1.00844205	0.31731655	0.6773938	-5.58959497
C9	P02748	0.11260339	23.354204	0.98824761	0.32702416	0.68285801	-5.60869649
ECM1	Q16610	0.09873696	20.9897077	0.98495517	0.32862542	0.68285801	-5.61177537
TFRC	P02786	0.1018015	18.8800306	0.96506691	0.33840859	0.69864999	-5.6301623

SAA2	P0DJI9	0.53277391	20.1494811	0.95596137	0.34295102	0.70348927	-5.63845937
CDH13	P55290	-0.10124657	17.3800791	-0.93176454	0.35521514	0.72400538	-5.66013747
GP1BA	P07359	0.11903202	18.814857	0.92591064	0.35822431	0.72551759	-5.66530107
CD5L	O43866	-0.25707945	22.1472539	-0.9121098	0.36538342	0.73091978	-5.67734949
HSPG2	P98160	-0.10502475	16.2833457	-0.90759727	0.36774401	0.73091978	-5.68125091
LUM	P51884	0.08052622	22.9092083	0.91060059	0.36617184	0.73091978	-5.6786564
SERPINA4	P29622	0.07701762	21.6161434	0.90212066	0.37062198	0.7320928	-5.6859606
IGHV1-46	P01743	-0.1428752	19.4193277	-0.88481915	0.37980782	0.73659699	-5.70065724
IGHV3-64	A0A075B6Q5	0.21194532	18.3688499	0.88805523	0.37807887	0.73659699	-5.69792941
LILRA3	Q8N6C8	0.17315692	16.1897443	0.8884016	0.37789411	0.73659699	-5.69763687
CA1	P00915	0.23553952	18.3004503	0.86941272	0.38810723	0.74751846	-5.71351106
FETUB	Q9UGM5	0.14136898	20.106348	0.8657237	0.39011119	0.74751846	-5.71655625
CD44	P16070	0.08041699	19.5336605	0.84172408	0.40330519	0.74870203	-5.73605922
IGHV4-34	P06331	-0.09106649	23.1731837	-0.81826147	0.41646551	0.74870203	-5.75460873
IGKV1-33	P01593;P01594	-0.15497612	20.727832	-0.82988345	0.40991447	0.74870203	-5.74548437
IGKV1-39	P01597;P04432	-0.16106104	21.2060114	-0.82217694	0.41425141	0.74870203	-5.75154875
IGKV6D-21	A0A0A0MT36	0.27087458	19.4592456	0.83681926	0.40603502	0.74870203	-5.73997927
LDHA	P00338	0.152326	16.9062251	0.83101705	0.40927887	0.74870203	-5.74458766
NRP1	O14786	-0.09549675	16.9259959	-0.85998339	0.39324225	0.74870203	-5.72126964
PCYOX1	Q9UHG3	-0.10928139	19.1231526	-0.82179688	0.414466	0.74870203	-5.75184639
RNASE4	P34096	-0.12591824	16.0139313	-0.83239414	0.40850755	0.74870203	-5.74349674
SOD3	P08294	-0.17412766	17.6936455	-0.83568025	0.40667056	0.74870203	-5.74088639
FBLN5	Q9UBX5	0.10920217	16.031945	0.80808097	0.42225573	0.75028574	-5.76249811
IGHV3-49	A0A0A0MS15	-0.09108449	24.211068	-0.81171115	0.42018552	0.75028574	-5.75969597
SERPINA3	P01011	0.06161278	24.9351385	0.80436647	0.42438037	0.75028574	-5.76535263
APOA1	P02647	-0.07598235	28.1793768	-0.78852928	0.43351063	0.75393153	-5.77737891
IGHV2-26	A0A0B4J1V2	0.17779214	20.8520535	0.78853811	0.43350551	0.75393153	-5.77737227
LPA	P08519	0.35329583	21.6972428	0.79154722	0.43176183	0.75393153	-5.77510522
APCS	P02743	0.14770902	17.2930456	0.76627301	0.44653648	0.76742006	-5.79388409
PROC	P04070	-0.09229007	19.91221	-0.76757373	0.44576898	0.76742006	-5.79293221
SAA4	P35542	0.09223732	22.9353759	0.76301699	0.4484611	0.76742006	-5.79625995
APOE	P02649	0.07939058	23.9459731	0.75531265	0.45303428	0.77112217	-5.80184218

IGHM	P01871	-0.14981332	25.7709008	-0.74529356	0.45902165	0.77481816	-5.80901851
IGKV1-5	P01602	-0.13567841	23.9956834	-0.74014394	0.46211664	0.77481816	-5.81267044
PROZ	P22891	-0.13544799	19.9926352	-0.73955796	0.46246959	0.77481816	-5.81308443
IGHV1-45	A0A0A0MS14	-0.25149515	18.1416658	-0.72305526	0.47247232	0.78745387	-5.82461114
IGHV3-74	A0A0B4J1X5	-0.10313376	21.7412932	-0.71628558	0.47661069	0.79023535	-5.82926573
CLEC3B	P05452	0.08468294	19.6079503	0.70843796	0.48143339	0.79411693	-5.83460766
APMAP	Q9HDC9	-0.13583152	18.8050855	-0.6907407	0.49240838	0.79985118	-5.84644202
IGHV1OR15-1	A0A075B7D0	-0.23227336	22.6438783	-0.69298744	0.49100748	0.79985118	-5.84495591
TF	P02787	-0.0533834	27.7570137	-0.69112457	0.49216887	0.79985118	-5.84618844
IGKV2D-30	A0A075B6S6	-0.07320157	23.8120391	-0.65663485	0.51394168	0.82643889	-5.86841819
ITIH2	P19823	0.05050391	24.1120568	0.65843669	0.51279162	0.82643889	-5.86728457
IGLV3-21	P80748	-0.14682924	19.6327168	-0.65109119	0.51748866	0.82798185	-5.87188676
CFHR5	Q9BXR6	0.07274633	19.1477789	0.64475853	0.52155629	0.83033837	-5.87581354
C8B	P07358	0.06152883	20.0495172	0.62683932	0.533157	0.83224507	-5.88671996
DSG1	Q02413	-0.16883261	17.4252669	-0.63566618	0.5274259	0.83224507	-5.88138541
IGHG3	P01860	0.07242021	28.8665791	0.63025236	0.53093716	0.83224507	-5.88466599
PTGDS	P41222	-0.09462297	18.1811839	-0.62837663	0.53215653	0.83224507	-5.88579616
A0A0J9YY99	A0A0J9YY99	-0.09036525	22.0311856	-0.60037842	0.5505289	0.8424998	-5.90227066
APOC3	P02656	-0.08024342	24.9339473	-0.59681422	0.55289049	0.8424998	-5.9043147
COL6A1	P12109	0.09507307	16.9046909	0.60066265	0.55034079	0.8424998	-5.90210714
FCGBP	Q9Y6R7	0.08625484	18.884819	0.60603091	0.54679409	0.8424998	-5.89900436
IGLL5	B9A064	-0.11021668	25.3316659	-0.60173064	0.54963427	0.8424998	-5.90149203
HSPA5	P11021	0.06767251	17.4981608	0.58324953	0.56192462	0.844206	-5.91198401
IGLV1-36	A0A0B4J1U3	-0.13944715	19.0465593	-0.58469582	0.56095791	0.844206	-5.91117459
IGLV1-47	P01700	-0.07783425	23.3917602	-0.59007508	0.55736964	0.844206	-5.90814669
IGHG1	P01857	-0.05700596	28.7073876	-0.57710787	0.56603891	0.84641332	-5.91539914
APOD	P05090	-0.05352848	23.0699581	-0.56290217	0.57561171	0.84975613	-5.92316141
FCN2	Q15485	-0.11172724	17.9890316	-0.56197255	0.57624088	0.84975613	-5.92366271
IGHA2	P01877	0.17667265	22.1453422	0.56566748	0.57374211	0.84975613	-5.92166538
IGKV3D-7	A0A0C4DH55	0.17527817	17.7412344	0.5575557	0.57923474	0.85025283	-5.92603328
ANGPTL6	Q8NI99	-0.09197512	15.7629167	-0.53985776	0.59130539	0.85343781	-5.93534634
ART3	Q13508	-0.07930138	15.573942	-0.53168156	0.59692162	0.85343781	-5.93954844

© Thieme. All rights reserved.

C1QC	P02747	0.04008588	24.0142247	0.53843977	0.59227763	0.85343781	-5.93607966
GAPDH	P04406	0.12502404	17.5641869	0.53277242	0.59617088	0.85343781	-5.93899147
IGLV3-10	A0A075B6K4	-0.14848848	20.4318482	-0.53097741	0.59740647	0.85343781	-5.93990736
PIGR	P01833	0.0823265	17.1835703	0.54350081	0.58881103	0.85343781	-5.93345358
COL6A3	P12111	0.03849292	17.197964	0.52230083	0.60339574	0.85816282	-5.94429138
APOB	P04114	0.05695469	24.4472065	0.51115269	0.61113144	0.86531885	-5.94981919
AHSG	P02765	-0.04350082	25.3216658	-0.49327705	0.62362848	0.87144591	-5.95843612
KNG1	P01042	0.03653043	25.1377497	0.49674101	0.62119794	0.87144591	-5.95679008
PTPRJ	Q12913	-0.06502566	17.2764298	-0.49413921	0.62302314	0.87144591	-5.9580275
IGHV1-69	P01742	-0.12954887	17.1223698	-0.48629327	0.62854153	0.87449256	-5.96172005
A0A0G2JRQ6	A0A0G2JRQ6	0.108092	22.4802718	0.46934023	0.64053794	0.87594932	-5.9694985
ALCAM	Q13740	0.07970886	15.1109893	0.47896284	0.63371664	0.87594932	-5.96511702
APOA4	P06727	0.05563435	24.7413481	0.47128785	0.63915477	0.87594932	-5.96861881
ICOSLG	O75144	-0.0581522	17.0655852	-0.47076634	0.63952501	0.87594932	-5.96885472
F5	P12259	0.04276039	19.7347952	0.4364532	0.66408405	0.89665357	-5.98380666
IGHV3OR15-7	A0A075B7D8	0.09429994	15.7468361	0.43877044	0.66241344	0.89665357	-5.98283228
C1QB	P02746	0.0440324	22.2223944	0.4206065	0.67555428	0.90092418	-5.99033263
COMP	P49747	0.04919857	19.0698012	0.41838468	0.67716875	0.90092418	-5.99122845
CP	P00450	-0.06082614	24.8396676	-0.41033598	0.68303001	0.90092418	-5.99443414
CPQ	Q9Y646	-0.0631771	15.4128594	-0.39727167	0.69258546	0.90092418	-5.99950568
GLIPR2	Q9H4G4	-0.04106115	17.2734087	-0.42341897	0.6735128	0.90092418	-5.9891919
HSP90B1	P14625	0.06232953	16.5477063	0.40047102	0.69024069	0.90092418	-5.99827878
HSPA6	P17066;P48741	0.0767608	16.7729238	0.40367367	0.68789654	0.90092418	-5.99704081
IGHV1-24	A0A0C4DH33	-0.1106122	18.6255518	-0.40467939	0.68716105	0.90092418	-5.99665004
SELL	P14151	0.04628823	19.114072	0.39819203	0.69191062	0.90092418	-5.99915373
PROCR	Q9UNN8	-0.06713873	18.1281379	-0.38535105	0.70134843	0.90862955	-6.00399086
IGKV3-11	A0A0A0MRZ8;P04433	-0.04407053	23.6207807	-0.38077768	0.70472128	0.90931779	-6.00567553
IGHA1	P01876	-0.07241592	28.3224079	-0.36531958	0.71616539	0.91303954	-6.01122162
IGHV1-18	A0A0C4DH31	-0.05716063	19.7091017	-0.36666608	0.71516588	0.91303954	-6.01074761
PRSS3	P35030	-0.07604514	18.5205826	-0.36681812	0.71505305	0.91303954	-6.01069398
HPX	P02790	-0.03209757	24.4706266	-0.36104759	0.71933977	0.91344732	-6.01271399
ADAMTS13	Q76LX8	0.03383185	16.3340031	0.34797223	0.72908633	0.91676334	-6.01717313

© Thieme. All rights reserved.

COLEC11	Q9BWP8	0.04859494	16.5591636	0.34601956	0.73054578	0.91676334	-6.017825
GPLD1	P80108	0.04054111	20.9791571	0.34763839	0.72933577	0.91676334	-6.01728483
CST3	P01034	0.03515922	20.1585677	0.2984301	0.76641229	0.91854656	-6.03258211
F2	P00734	0.02463209	22.6089595	0.31316691	0.7552465	0.91854656	-6.02824447
FCGR3A	P08637	0.04197634	18.020512	0.30096134	0.7644908	0.91854656	-6.03185188
HP	P00738	0.05078046	27.9014485	0.32192027	0.74863883	0.91854656	-6.02556939
IGHV1-3	A0A0C4DH29	-0.07208673	18.6271843	-0.31853611	0.75119122	0.91854656	-6.02661232
IGKV3D-20	A0A0C4DH25	-0.04754688	22.2982795	-0.31512358	0.75376785	0.91854656	-6.02765288
ITIH4	Q14624	0.02172663	25.4082487	0.3336726	0.73979706	0.91854656	-6.02186227
MMP2	P08253	0.04641532	15.8005296	0.31929373	0.75061957	0.91854656	-6.02637979
PON3	Q15166	-0.02946871	18.9049407	-0.29886913	0.76607891	0.91854656	-6.03245589
RARRES2	Q99969	-0.0533724	17.1747954	-0.30589823	0.76074745	0.91854656	-6.03040997
SERPINC1	P01008	0.01708899	24.6991753	0.30413736	0.76208196	0.91854656	-6.03092695
SPP2	Q13103	0.05446871	18.4606221	0.30814506	0.75904571	0.91854656	-6.02974601
IGFBP3	P17936	0.03641405	20.0712664	0.28799763	0.77434709	0.92117258	-6.03552682
LCAT	P04180	-0.0218763	20.121504	-0.28797977	0.7743607	0.92117258	-6.03553178
IGHV4-4	A0A075B6R2	-0.04422264	17.9523274	-0.28269898	0.77838652	0.92253217	-6.03698244
APOH	P02749	0.03614036	23.1352858	0.26558409	0.7914755	0.92336429	-6.0415
C7	P10643	-0.02331308	20.9865577	-0.26430059	0.79245957	0.92336429	-6.04182745
FCGR3B	O75015	0.04818248	17.3514979	0.2732553	0.78560107	0.92336429	-6.03950993
IGKV2-30	P06310	0.05548064	18.1425355	0.27794072	0.78201921	0.92336429	-6.03826664
PGLYRP2	Q96PD5	0.02328671	21.988419	0.26292297	0.79351618	0.92336429	-6.04217714
TNXB	P22105	-0.03386562	18.0418559	-0.25690908	0.79813326	0.9253719	-6.04368237
IGLV2-8	P01709	-0.05979641	19.8001228	-0.25302806	0.80111672	0.9254778	-6.04463531
Biognosys	Biognosys	-0.03213462	25.624147	-0.24689092	0.80584059	0.92758629	-6.04611267
FBLN1	P23142	0.02705525	20.2169719	0.23972129	0.81136837	0.93060172	-6.04779274
AGT	P01019	0.03490003	23.1739123	0.2308843	0.81819496	0.93447338	-6.04979554
C8G	P07360	0.02321528	20.9233434	0.21833964	0.82790993	0.93447338	-6.0525097
CPN1	P15169	0.0233764	20.4565502	0.21790026	0.8282507	0.93447338	-6.05260203
IL1RAP	Q9NPH3	-0.03803296	18.0207882	-0.21284991	0.83216996	0.93447338	-6.05364986
ITIH1	P19827	-0.01750706	23.7553938	-0.2213206	0.82559888	0.93447338	-6.05187845
PKM	P14618	-0.04281356	16.3916108	-0.21272704	0.83226536	0.93447338	-6.05367505

© Thieme. All rights reserved.

CHL1	O00533	0.02293775	18.652861	0.20708142	0.83665174	0.93502396	-6.05481668
F7	P08709	-0.02676689	18.1425447	-0.20082141	0.84152156	0.93502396	-6.0560467
RBP4	P02753	-0.03137738	22.6580325	-0.20261328	0.84012698	0.93502396	-6.05569847
IGKV3-20	P01619	-0.03557793	24.6834846	-0.19371995	0.84705349	0.9379139	-6.05739642
LCP1	P13796	0.0261307	17.5959815	0.17645786	0.86053213	0.94378204	-6.06047493
PON1	P27169	-0.02059024	21.868587	-0.17560254	0.86120111	0.94378204	-6.06062001
PVR	P15151	0.0273238	17.7619787	0.18061139	0.857285	0.94378204	-6.05976039
CTSD	P07339	-0.03982986	16.7155848	-0.15778197	0.87516164	0.94938765	-6.06348257
GSN	P06396	0.01374424	22.7201539	0.15771174	0.87521674	0.94938765	-6.06349325
IGLV3-19	P01714	-0.04086126	17.4361385	-0.16181531	0.87199829	0.94938765	-6.06286145
F12	P00748	0.02310046	23.1657455	0.14392768	0.88604284	0.95788415	-6.06549675
A1BG	P04217	0.00803455	24.7163609	0.11339154	0.91010101	0.95800106	-6.06928343
APOM	O95445	0.01150805	22.7148828	0.12452484	0.90131853	0.95800106	-6.06800687
ATRN	O75882	0.01074284	19.3221815	0.12792653	0.89863753	0.95800106	-6.06759302
CDH5	P33151	-0.01291793	19.453447	-0.13915039	0.88980016	0.95800106	-6.06614843
GPX3	P22352	-0.01377571	20.1751647	-0.12566603	0.90041899	0.95800106	-6.06786928
HRG	P04196	0.0114067	24.0252503	0.11830946	0.90622006	0.95800106	-6.06873426
ITIH3	Q06033	0.01746545	21.1968257	0.13489725	0.89314738	0.95800106	-6.06671012
PLG	P00747	-0.00905628	23.5014502	-0.11512889	0.90872974	0.95800106	-6.06909209
CFD	P00746	0.01407929	19.0087585	0.09819099	0.92210992	0.95863629	-6.07083351
CRTAC1	Q9NQ79	-0.00841038	25.5056031	-0.09470973	0.9248629	0.95863629	-6.07115718
EFEMP1	Q12805	0.00819994	18.4593266	0.09142972	0.92745757	0.95863629	-6.07145145
IGLV2-18	A0A075B6J9	-0.02437196	18.1815247	-0.10266431	0.91857384	0.95863629	-6.07040046
IGLV5-45	A0A087WSX0	0.0235593	17.6019328	0.09478685	0.9248019	0.95863629	-6.07115013
JCHAIN	P01591	-0.01530205	24.155897	-0.08988613	0.92867891	0.95863629	-6.07158634
IGKV1-16	P04430	-0.01351302	19.2186933	-0.07822355	0.93791211	0.96505426	-6.07253135
CD14	P08571	0.00873422	17.4982571	0.0718594	0.94295425	0.96713257	-6.07299174
LYVE1	Q9Y5Y7	0.00954025	19.1043226	0.06227503	0.95055206	0.97181042	-6.07361142
PRR4	Q16378	0.01049224	16.2552605	0.04497298	0.9642791	0.98270482	-6.07450589
CFHR1	Q03591	-0.0068799	19.0183948	-0.0345391	0.97256261	0.9849056	-6.07490581
DCD	P81605	-0.00689005	17.9474933	-0.02745629	0.97818748	0.9849056	-6.07511748
IGLV3-27	P01718	0.00489971	20.3065837	0.02910555	0.97687759	0.9849056	-6.07507251

THBS4	P35443	0.00443512	16.3012127	0.02674812	0.97874994	0.9849056	-6.07513598
ALB	P02768	0	32.0336323	0	1	1	-6.07548087
IGLV3-9	A0A075B6K5	1.8791E-05	20.2520486	0.00010357	0.99991771	1	-6.07548087

© Thieme. All rights reserved.

Table S3. Comparison of plasma protein abundance between type 1 diabetes (both with and without IR) group versus control group, adjusted for sex as a covariate. Differential protein abundance was assessed using linear regression models (protein ~ group + sex), with the pairwise contrast type 1 diabetes vs control. P-values were adjusted for multiple testing using the Benjamini–Hochberg false discovery rate (FDR), with significance defined as p <0.05.

Protein (gene name)	UniProtID	logFC	AveExpr	t	P.Value	adj.P.Val	B
RBP4	P02753	-0.677	22.7	-5.13	3.44e-06	5.50e-04	4.30
FCGR3B	O75015	-0.698	17.4	-4.48	3.46e-05	2.77e-03	2.14
PRDX2	P32119	0.844	17.3	4.11	1.25e-04	7.98e-03	0.939
CA1	P00915	0.921	18.3	3.87	2.78e-04	1.27e-02	0.195
ITIH3	Q06033	0.420	21.2	3.89	2.60e-04	1.27e-02	0.258
FN1	P02751	-0.456	23.3	-3.47	9.85e-04	3.58e-02	-0.970
GPLD1	P80108	-0.343	21.0	-3.46	1.01e-03	3.58e-02	-0.989
C2	P06681	0.250	21.0	3.41	1.17e-03	3.75e-02	-1.13
LILRA3	Q8N6C8	-0.534	16.2	-3.31	1.61e-03	4.44e-02	-1.42
LYZ	P61626	-0.347	20.0	-3.24	1.94e-03	4.44e-02	-1.59
PCYOX1	Q9UHG3	-0.381	19.1	-3.29	1.70e-03	4.44e-02	-1.47
TF	P02787	-0.221	27.8	-3.26	1.88e-03	4.44e-02	-1.56
IGFALS	P35858	-0.305	21.3	-3.17	2.44e-03	5.21e-02	-1.80
ADAMTS13	Q76LX8	-0.264	16.3	-3.08	3.15e-03	6.30e-02	-2.03
CFD	P00746	-0.383	19.0	-3.05	3.42e-03	6.43e-02	-2.10
TTR	P02766	-0.248	25.6	-2.91	5.13e-03	9.12e-02	-2.47
ABCB9	Q9NP78	0.811	19.8	2.89	5.42e-03	9.13e-02	-2.52
AFM	P43652	-0.235	22.5	-2.74	8.17e-03	1.31e-01	-2.88
IGFBP3	P17936	-0.298	20.1	-2.68	9.63e-03	1.47e-01	-3.03
IGHV3OR15-	A0A075B7D8	-0.502	15.7	-2.65	1.04e-02	1.52e-01	-3.10

7							
CNDP1	Q96KN2	-0.345	19.3	-2.51	1.50e-02	2.00e-01	-3.42
SOD3	P08294	-0.462	17.7	-2.51	1.49e-02	2.00e-01	-3.41
AGT	P01019	-0.311	23.2	-2.38	2.03e-02	2.15e-01	-3.68
APOF	Q13790	0.260	19.9	2.38	2.04e-02	2.15e-01	-3.68
CAMP	P49913	0.577	18.6	2.38	2.08e-02	2.15e-01	-3.70
HSPG2	P98160	-0.247	16.3	-2.44	1.76e-02	2.15e-01	-3.56
LUM	P51884	0.184	22.9	2.40	1.98e-02	2.15e-01	-3.66
SERPINA1	P01009	0.202	29.6	2.43	1.84e-02	2.15e-01	-3.59
APOC1	P02654	-0.262	22.3	-2.35	2.19e-02	2.15e-01	-3.75
F11	P03951	0.217	20.1	2.31	2.46e-02	2.15e-01	-3.85
IGHV1-2	P23083	-0.293	18.4	-2.33	2.31e-02	2.15e-01	-3.79
LTF	P02788	-0.323	17.6	-2.33	2.32e-02	2.15e-01	-3.80
PKM	P14618	0.404	16.4	2.30	2.49e-02	2.15e-01	-3.86
TGFBI	Q15582	-0.209	18.8	-2.31	2.47e-02	2.15e-01	-3.85
COL6A3	P12111	-0.145	17.2	-2.26	2.76e-02	2.20e-01	-3.94
FETUB	Q9UGM5	-0.325	20.1	-2.27	2.69e-02	2.20e-01	-3.92
HABP2	Q14520	-0.205	18.9	-2.26	2.72e-02	2.20e-01	-3.93
APMAP	Q9HDC9	0.390	18.8	2.24	2.90e-02	2.21e-01	-3.99
VCAM1	P19320	0.222	18.7	2.25	2.83e-02	2.21e-01	-3.97
IGHA2	P01877	-0.612	22.1	-2.22	3.01e-02	2.24e-01	-4.02
LGALS3BP	Q08380	-0.303	19.4	-2.21	3.07e-02	2.24e-01	-4.04
GAPDH	P04406	0.452	17.6	2.20	3.19e-02	2.27e-01	-4.07
IGHV3-35	A0A0C4DH35	-0.264	19.0	-2.16	3.50e-02	2.38e-01	-4.15
SAA4	P35542	-0.231	22.9	-2.16	3.48e-02	2.38e-01	-4.14
IGHV4-28	A0A0C4DH34	-0.335	20.3	-2.13	3.70e-02	2.47e-01	-4.19

PIP	P12273	-0.347	16.9	-2.11	3.92e-02	2.56e-01	-4.24
B2M	P61769	-0.257	18.6	-2.06	4.39e-02	2.75e-01	-4.34
FBLN1	P23142	0.201	20.2	2.05	4.47e-02	2.75e-01	-4.35
IGKV1-13	A0A0B4J2D9;P0DP09	0.608	19.1	2.06	4.42e-02	2.75e-01	-4.34
HSPA6	P17066;P48741	0.336	16.8	2.00	5.03e-02	3.01e-01	-4.45
LDHA	P00338	0.317	16.9	1.98	5.27e-02	3.01e-01	-4.49
PPBP	P02775	0.363	19.3	1.98	5.27e-02	3.01e-01	-4.49
SERPINA4	P29622	-0.148	21.6	-1.99	5.17e-02	3.01e-01	-4.47
A2M	P01023	0.208	27.3	1.95	5.56e-02	3.07e-01	-4.53
PROCR	Q9UNN8	0.297	18.1	1.95	5.55e-02	3.07e-01	-4.53
IGLV1-36	A0A0B4J1U3	-0.410	19.0	-1.94	5.66e-02	3.07e-01	-4.55
IGLV7-46	A0A075B6I9	0.288	23.4	1.91	6.10e-02	3.25e-01	-4.61
APOM	O95445	0.153	22.7	1.88	6.52e-02	3.38e-01	-4.67
IGKV1D-16	P01601	-0.402	17.9	-1.88	6.56e-02	3.38e-01	-4.67
IGHG4	P01861	-0.150	29.9	-1.85	6.99e-02	3.55e-01	-4.72
APOA4	P06727	0.181	24.7	1.81	7.47e-02	3.68e-01	-4.78
GC	P02774	-0.122	24.0	-1.82	7.41e-02	3.68e-01	-4.77
CST3	P01034	-0.184	20.2	-1.80	7.67e-02	3.72e-01	-4.80
C1R	P00736	0.118	21.4	1.79	7.92e-02	3.73e-01	-4.82
IGHV2-5	P01817	0.540	17.2	1.79	7.93e-02	3.73e-01	-4.83
APOD	P05090	0.144	23.1	1.77	8.20e-02	3.75e-01	-4.85
IGHV2-70D	A0A0C4DH43	0.820	20.8	1.77	8.20e-02	3.75e-01	-4.85
CETP	P11597	0.193	19.6	1.75	8.49e-02	3.83e-01	-4.88
CLU	P10909	-0.115	24.5	-1.71	9.20e-02	3.96e-01	-4.95
CRTAC1	Q9NQ79	-0.135	25.5	-1.71	9.17e-02	3.96e-01	-4.94

IGKV2D-30	A0A075B6S6	0.163	23.8	1.72	9.04e-02	3.96e-01	-4.93
IGLC3	P0DOY3	-0.162	26.9	-1.71	9.29e-02	3.96e-01	-4.95
PIGR	P01833	0.226	17.2	1.70	9.46e-02	3.98e-01	-4.97
ORM2	P19652	-0.161	23.3	-1.69	9.70e-02	4.03e-01	-4.99
ECM1	Q16610	-0.143	21.0	-1.67	9.95e-02	4.04e-01	-5.01
GSN	P06396	-0.124	22.7	-1.67	9.97e-02	4.04e-01	-5.01
ALDOA	P04075	0.357	17.5	1.58	1.19e-01	4.33e-01	-5.15
ANTXR1	Q9H6X2	0.177	16.1	1.61	1.12e-01	4.33e-01	-5.10
AZGP1	P25311	-0.196	21.0	-1.59	1.18e-01	4.33e-01	-5.14
CD14	P08571	0.169	17.5	1.59	1.16e-01	4.33e-01	-5.13
F13B	P05160	-0.131	20.1	-1.61	1.12e-01	4.33e-01	-5.10
IGHV1-45	A0A0A0MS14	-0.487	18.1	-1.58	1.19e-01	4.33e-01	-5.15
IGHV3OR16-9	A0A0B4J2B5	0.196	25.4	1.60	1.16e-01	4.33e-01	-5.13
ITIH1	P19827	-0.112	23.8	-1.61	1.13e-01	4.33e-01	-5.11
THBS4	P35443	-0.232	16.3	-1.58	1.19e-01	4.33e-01	-5.15
VTN	P04004	-0.0921	24.3	-1.57	1.22e-01	4.40e-01	-5.17
B4GALT1	P15291	-0.243	15.0	-1.56	1.25e-01	4.42e-01	-5.19
PVR	P15151	-0.208	17.8	-1.55	1.26e-01	4.42e-01	-5.19
IGKV6-21	A0A0C4DH24	0.237	20.4	1.54	1.29e-01	4.47e-01	-5.21
IGLV1-51	P01701	-0.140	21.5	-1.52	1.33e-01	4.54e-01	-5.23
PROS1	P07225	-0.0997	22.0	-1.52	1.33e-01	4.54e-01	-5.24
IGKV2D-24	A0A075B6R9	0.195	22.4	1.50	1.39e-01	4.57e-01	-5.27
IGLV7-43	P04211	-0.223	18.7	-1.51	1.37e-01	4.57e-01	-5.26
MAN1A1	P33908	0.104	18.2	1.51	1.37e-01	4.57e-01	-5.26
MASP2	O00187	0.186	20.6	1.50	1.40e-01	4.57e-01	-5.27

CASP14	P31944	0.358	16.5	1.47	1.46e-01	4.58e-01	-5.31
F13A1	P00488	-0.107	20.5	-1.48	1.43e-01	4.58e-01	-5.29
HGFAC	Q04756	0.175	22.0	1.48	1.45e-01	4.58e-01	-5.30
IGHV3-72	A0A0B4J1Y9	0.203	16.8	1.47	1.48e-01	4.58e-01	-5.31
IGKV1-12	A0A0C4DH73;P01611	-0.306	18.8	-1.45	1.52e-01	4.58e-01	-5.33
IGKV1-37	A0A075B6S9;P0DSN7	-0.315	17.8	-1.46	1.49e-01	4.58e-01	-5.32
IGKV3D-15	A0A087WSY6	-0.190	23.2	-1.46	1.50e-01	4.58e-01	-5.33
MBL2	P11226	0.331	19.2	1.47	1.47e-01	4.58e-01	-5.31
PGLYRP2	Q96PD5	0.109	22.0	1.45	1.54e-01	4.59e-01	-5.34
APOL1	O14791	-0.154	22.2	-1.44	1.55e-01	4.60e-01	-5.35
F5	P12259	0.123	19.7	1.42	1.60e-01	4.70e-01	-5.38
C3	P01024	-0.0860	27.4	-1.40	1.65e-01	4.73e-01	-5.40
IGLV5-45	A0A087WSX0	-0.306	17.6	-1.40	1.66e-01	4.73e-01	-5.40
LRP1	Q07954	0.194	15.6	1.40	1.66e-01	4.73e-01	-5.40
IGKV2-24	A0A0C4DH68	0.252	18.8	1.39	1.69e-01	4.79e-01	-5.42
C9	P02748	0.134	23.4	1.38	1.72e-01	4.80e-01	-5.43
IGHV6-1	A0A0B4J1U7	0.148	23.2	1.38	1.71e-01	4.80e-01	-5.43
A0A0J9YY99	A0A0J9YY99	0.179	22.0	1.36	1.80e-01	4.82e-01	-5.46
C1QC	P02747	0.0887	24.0	1.36	1.80e-01	4.82e-01	-5.46
CD44	P16070	0.109	19.5	1.35	1.83e-01	4.82e-01	-5.48
CDH5	P33151	0.110	19.5	1.36	1.79e-01	4.82e-01	-5.46
IGHV3-43	A0A0B4J1X8	0.145	20.4	1.36	1.79e-01	4.82e-01	-5.46
IGLV3-10	A0A075B6K4	-0.333	20.4	-1.34	1.84e-01	4.82e-01	-5.48
IGLV5-37	A0A075B6J1	0.380	17.6	1.35	1.82e-01	4.82e-01	-5.47

F2	P00734	-0.0926	22.6	-1.34	1.86e-01	4.84e-01	-5.49
RARRES2	Q99969	-0.203	17.2	-1.33	1.89e-01	4.87e-01	-5.50
CPQ	Q9Y646	0.185	15.4	1.32	1.93e-01	4.95e-01	-5.51
F12	P00748	-0.181	23.2	-1.28	2.06e-01	5.20e-01	-5.56
PRG4	Q92954	0.166	20.0	1.28	2.06e-01	5.20e-01	-5.56
IGHD	P01880	-0.341	20.4	-1.27	2.10e-01	5.20e-01	-5.57
LRG1	P02750	0.151	22.8	1.27	2.09e-01	5.20e-01	-5.57
CLEC3B	P05452	-0.132	19.6	-1.24	2.18e-01	5.37e-01	-5.60
FGA	P02671	0.0700	26.9	1.22	2.27e-01	5.55e-01	-5.63
RNASE4	P34096	-0.160	16.0	-1.20	2.36e-01	5.68e-01	-5.66
VASN	Q6EMK4	0.0813	19.0	1.20	2.36e-01	5.68e-01	-5.66
IGHV2-26	A0A0B4J1V2	-0.216	20.9	-1.17	2.48e-01	5.93e-01	-5.69
CTSD	P07339	0.253	16.7	1.14	2.60e-01	5.98e-01	-5.73
IGHV5-51	A0A0C4DH38	-0.185	20.5	-1.14	2.59e-01	5.98e-01	-5.72
IGKV3D-7	A0A0C4DH55	0.319	17.7	1.15	2.55e-01	5.98e-01	-5.71
IGLV2-11	P01706	-0.127	22.5	-1.15	2.54e-01	5.98e-01	-5.71
ORM1	P02763	-0.117	25.2	-1.14	2.58e-01	5.98e-01	-5.72
IGKC	P01834	-0.112	24.6	-1.13	2.64e-01	6.00e-01	-5.74
PRSS3	P35030	-0.206	18.5	-1.13	2.64e-01	6.00e-01	-5.74
CP	P00450	0.139	24.8	1.08	2.85e-01	6.31e-01	-5.79
FCGBP	Q9Y6R7	0.133	18.9	1.08	2.86e-01	6.31e-01	-5.79
IGHV3-38	A0A0C4DH36	0.167	18.5	1.08	2.83e-01	6.31e-01	-5.78
IGHV3-64	A0A075B6Q5	0.229	18.4	1.09	2.82e-01	6.31e-01	-5.78
F9	P00740	-0.0848	19.7	-1.05	3.00e-01	6.36e-01	-5.82
IGLV2-14	P01704	-0.205	18.4	-1.05	2.99e-01	6.36e-01	-5.82
MCAM	P43121	0.0972	17.0	1.05	2.97e-01	6.36e-01	-5.82

© Thieme. All rights reserved.

PTPRJ	Q12913	-0.123	17.3	-1.06	2.93e-01	6.36e-01	-5.81
SERPINC1	P01008	-0.0514	24.7	-1.05	2.99e-01	6.36e-01	-5.82
TFRC	P02786	-0.0975	18.9	-1.05	2.98e-01	6.36e-01	-5.82
C1QB	P02746	0.0952	22.2	1.04	3.03e-01	6.39e-01	-5.83
APOE	P02649	0.0947	23.9	1.02	3.12e-01	6.52e-01	-5.85
APOC2	P02655	0.106	22.7	0.950	3.46e-01	6.54e-01	-5.92
C6	P13671	-0.0768	20.8	-0.992	3.25e-01	6.54e-01	-5.88
FCN2	Q15485	-0.176	18.0	-1.01	3.15e-01	6.54e-01	-5.85
FGB	P02675	0.0745	25.9	0.946	3.48e-01	6.54e-01	-5.92
HP	P00738	0.135	27.9	0.967	3.37e-01	6.54e-01	-5.90
IGF2R	P11717	0.103	16.2	0.997	3.23e-01	6.54e-01	-5.87
IGHG3	P01860	-0.0962	28.9	-0.952	3.45e-01	6.54e-01	-5.91
IGHV1-46	P01743	-0.135	19.4	-0.960	3.41e-01	6.54e-01	-5.91
IGKJ1	A0A0A0MT89	0.165	18.4	0.941	3.51e-01	6.54e-01	-5.92
IGLV3-19	P01714	-0.220	17.4	-0.991	3.26e-01	6.54e-01	-5.88
IL1RAP	Q9NPH3	0.158	18.0	1.01	3.19e-01	6.54e-01	-5.86
ITIH2	P19823	-0.0671	24.1	-0.993	3.25e-01	6.54e-01	-5.87
MMP2	P08253	0.120	15.8	0.939	3.51e-01	6.54e-01	-5.93
NID1	P14543	-0.101	16.3	-0.963	3.39e-01	6.54e-01	-5.90
PLG	P00747	-0.0668	23.5	-0.962	3.40e-01	6.54e-01	-5.90
PLXDC2	Q6UX71	0.106	17.0	0.952	3.45e-01	6.54e-01	-5.91
PTGDS	P41222	-0.126	18.2	-0.986	3.28e-01	6.54e-01	-5.88
SERPIND1	P05546	-0.0900	23.2	-0.952	3.45e-01	6.54e-01	-5.91
SERPINF1	P36955	-0.0741	21.2	-0.967	3.37e-01	6.54e-01	-5.90
HPR	P00739	-0.120	21.9	-0.935	3.54e-01	6.54e-01	-5.93
IGHV3OR16-	A0A075B7B8	0.140	23.0	0.924	3.59e-01	6.60e-01	-5.94

© Thieme. All rights reserved.

12							
COMP	P49747	0.0935	19.1	0.920	3.61e-01	6.61e-01	-5.94
ATRN	O75882	0.0672	19.3	0.907	3.68e-01	6.70e-01	-5.95
ALCAM	Q13740	0.125	15.1	0.850	3.99e-01	7.09e-01	-6.00
HPX	P02790	-0.0669	24.5	-0.851	3.98e-01	7.09e-01	-6.00
ICOSLG	O75144	0.0909	17.1	0.855	3.96e-01	7.09e-01	-6.00
MASP1	P48740	0.0860	17.6	0.858	3.95e-01	7.09e-01	-6.00
IGKV1-33	P01593;P01594	-0.139	20.7	-0.843	4.03e-01	7.12e-01	-6.01
APOC4	P55056	0.0956	19.1	0.816	4.18e-01	7.18e-01	-6.03
HRG	P04196	0.0670	24.0	0.817	4.17e-01	7.18e-01	-6.03
HSPA5	P11021	0.0849	17.5	0.829	4.11e-01	7.18e-01	-6.02
IGHV4-34	P06331	-0.0809	23.2	-0.826	4.12e-01	7.18e-01	-6.02
IGKV1-8	A0A0C4DH67;A0A0C4DH69	-0.145	20.5	-0.811	4.21e-01	7.18e-01	-6.03
PRR4	Q16378	0.169	16.3	0.824	4.13e-01	7.18e-01	-6.02
SERPING1	P05155	0.0625	24.4	0.809	4.22e-01	7.18e-01	-6.04
Biognosys	Biognosys	0.0917	25.6	0.797	4.29e-01	7.26e-01	-6.05
C4BPA	P04003	0.0518	22.6	0.779	4.39e-01	7.34e-01	-6.06
CFB	P00751	0.0574	24.4	0.784	4.36e-01	7.34e-01	-6.06
SELENOP	P49908	-0.0730	20.1	-0.777	4.40e-01	7.34e-01	-6.06
IGHV3-7	P01780	0.0686	24.0	0.765	4.47e-01	7.41e-01	-6.07
ANGPTL6	Q8NI99	0.111	15.8	0.739	4.63e-01	7.45e-01	-6.09
APCS	P02743	-0.124	17.3	-0.735	4.65e-01	7.45e-01	-6.09
C8B	P07358	0.0633	20.0	0.745	4.59e-01	7.45e-01	-6.08
HSP90B1	P14625	0.104	16.5	0.757	4.52e-01	7.45e-01	-6.08
IGKV4-1	P06312	-0.136	22.7	-0.750	4.56e-01	7.45e-01	-6.08

IGLV3-27	P01718	-0.109	20.3	-0.737	4.64e-01	7.45e-01	-6.09
JCHAIN	P01591	0.110	24.2	0.743	4.60e-01	7.45e-01	-6.09
IGLL1	P15814	-0.156	17.4	-0.730	4.68e-01	7.45e-01	-6.10
IGLV1-47	P01700	-0.0832	23.4	-0.720	4.75e-01	7.52e-01	-6.10
DBH	P09172	-0.140	18.2	-0.703	4.85e-01	7.64e-01	-6.11
C8A	P07357	0.0519	21.6	0.677	5.01e-01	7.72e-01	-6.13
IGHV3-38-3	P0DTE1	0.104	18.5	0.685	4.96e-01	7.72e-01	-6.13
IGKV1-17	P01599	0.0782	21.7	0.676	5.01e-01	7.72e-01	-6.13
IGLV2-8	P01709	0.143	19.8	0.684	4.96e-01	7.72e-01	-6.13
KNG1	P01042	-0.0437	25.1	-0.676	5.02e-01	7.72e-01	-6.13
IGHG1	P01857	-0.0568	28.7	-0.651	5.18e-01	7.92e-01	-6.15
C4A	P0C0L4	-0.108	22.8	-0.634	5.29e-01	7.93e-01	-6.16
CFI	P05156	-0.0462	22.3	-0.613	5.42e-01	7.93e-01	-6.17
GPX3	P22352	-0.0584	20.2	-0.635	5.28e-01	7.93e-01	-6.16
IGHV1-18	A0A0C4DH31	-0.0834	19.7	-0.613	5.43e-01	7.93e-01	-6.17
IGHV1-3	A0A0C4DH29	-0.123	18.6	-0.620	5.38e-01	7.93e-01	-6.17
IGHV3-49	A0A0A0MS15	-0.0612	24.2	-0.618	5.39e-01	7.93e-01	-6.17
IGKV1-27	A0A075B6S5	-0.0954	20.5	-0.632	5.30e-01	7.93e-01	-6.16
IGKV3-20	P01619	0.102	24.7	0.627	5.33e-01	7.93e-01	-6.16
NRP1	O14786	-0.0620	16.9	-0.641	5.24e-01	7.93e-01	-6.15
SHBG	P04278	0.118	19.1	0.631	5.30e-01	7.93e-01	-6.16
CFH	P08603	-0.0406	23.6	-0.600	5.51e-01	7.97e-01	-6.18
IGLC7	A0M8Q6	-0.0747	23.6	-0.601	5.50e-01	7.97e-01	-6.18
FCGR3A	P08637	-0.0721	18.0	-0.586	5.60e-01	8.07e-01	-6.19
SERPINA5	P05154	-0.0457	20.7	-0.581	5.64e-01	8.09e-01	-6.19
ITIH4	Q14624	0.0319	25.4	0.571	5.70e-01	8.14e-01	-6.20

© Thieme. All rights reserved.

LBP	P18428	0.0744	19.6	0.566	5.74e-01	8.16e-01	-6.20
AHSG	P02765	-0.0431	25.3	-0.553	5.82e-01	8.24e-01	-6.21
C1S	P09871	0.0287	23.7	0.531	5.97e-01	8.42e-01	-6.22
CD5L	O43866	-0.126	22.1	-0.513	6.10e-01	8.54e-01	-6.23
IGKV3-7	A0A075B6H7	-0.0941	25.2	-0.511	6.11e-01	8.54e-01	-6.23
BCHE	P06276	-0.0419	19.6	-0.476	6.36e-01	8.76e-01	-6.25
EFEMP1	Q12805	-0.0376	18.5	-0.474	6.38e-01	8.76e-01	-6.25
F10	P00742	-0.0323	20.7	-0.480	6.33e-01	8.76e-01	-6.24
IGHV3-74	A0A0B4J1X5	0.0606	21.7	0.479	6.34e-01	8.76e-01	-6.24
IGKV3-11	A0A0A0MRZ8;P04433	-0.0478	23.6	-0.470	6.40e-01	8.76e-01	-6.25
IGHV1-69	P01742	-0.108	17.1	-0.457	6.49e-01	8.83e-01	-6.25
LYVE1	Q9Y5Y7	0.0616	19.1	0.455	6.51e-01	8.83e-01	-6.26
CDH13	P55290	0.0409	17.4	0.426	6.71e-01	8.91e-01	-6.27
COLEC11	Q9BWP8	-0.0510	16.6	-0.419	6.77e-01	8.91e-01	-6.27
GP1BA	P07359	-0.0479	18.8	-0.423	6.74e-01	8.91e-01	-6.27
IGHM	P01871	0.0710	25.8	0.435	6.65e-01	8.91e-01	-6.26
IGHV1-24	A0A0C4DH33	-0.102	18.6	-0.434	6.66e-01	8.91e-01	-6.26
IGHV3-9	P01782	-0.0647	18.4	-0.422	6.75e-01	8.91e-01	-6.27
SPP2	Q13103	0.0671	18.5	0.430	6.69e-01	8.91e-01	-6.27
ART3	Q13508	-0.0517	15.6	-0.404	6.88e-01	9.00e-01	-6.28
IGHV3-64D	A0A0J9YX35	0.0632	21.9	0.402	6.89e-01	9.00e-01	-6.28
AMBP	P02760	0.0254	23.1	0.385	7.02e-01	9.11e-01	-6.28
CFHR1	Q03591	0.0675	19.0	0.383	7.03e-01	9.11e-01	-6.28
CFHR5	Q9BXR6	0.0365	19.1	0.372	7.12e-01	9.12e-01	-6.29
FBLN5	Q9UBX5	-0.0443	16.0	-0.370	7.13e-01	9.12e-01	-6.29

 © Thieme. All rights reserved.

SERPINF2	P08697	0.0228	21.7	0.373	7.11e-01	9.12e-01	-6.29
APOA1	P02647	0.0294	28.2	0.348	7.29e-01	9.13e-01	-6.30
APOB	P04114	0.0339	24.4	0.345	7.32e-01	9.13e-01	-6.30
IGHV3-13	P01766	-0.0404	19.7	-0.338	7.37e-01	9.13e-01	-6.30
IGKV1-5	P01602	0.0545	24.0	0.336	7.38e-01	9.13e-01	-6.30
IGLL5	B9A064	-0.0537	25.3	-0.331	7.42e-01	9.13e-01	-6.30
LCAT	P04180	-0.0225	20.1	-0.336	7.38e-01	9.13e-01	-6.30
PI16	Q6UXB8	-0.0430	18.4	-0.354	7.25e-01	9.13e-01	-6.30
PROZ	P22891	-0.0555	20.0	-0.343	7.33e-01	9.13e-01	-6.30
SERPINA3	P01011	-0.0227	24.9	-0.337	7.37e-01	9.13e-01	-6.30
SERPINA7	P05543	0.0255	20.8	0.334	7.39e-01	9.13e-01	-6.30
IGKV1-6	A0A0C4DH72	0.0498	20.8	0.317	7.52e-01	9.19e-01	-6.31
SAA2	P0DJI9	-0.148	20.1	-0.317	7.52e-01	9.19e-01	-6.31
PON3	Q15166	0.0262	18.9	0.303	7.63e-01	9.25e-01	-6.31
PROC	P04070	-0.0321	19.9	-0.306	7.61e-01	9.25e-01	-6.31
IGHV3-15	A0A0B4J1V0	-0.0378	23.2	-0.295	7.69e-01	9.29e-01	-6.31
A1BG	P04217	-0.0150	24.7	-0.240	8.11e-01	9.31e-01	-6.33
C1RL	Q9NZP8	0.0193	18.7	0.231	8.18e-01	9.31e-01	-6.33
CHL1	O00533	-0.0224	18.7	-0.230	8.19e-01	9.31e-01	-6.33
DCD	P81605	0.0574	17.9	0.260	7.95e-01	9.31e-01	-6.32
F7	P08709	-0.0310	18.1	-0.263	7.93e-01	9.31e-01	-6.32
IGHG2	P01859	0.0298	28.3	0.240	8.11e-01	9.31e-01	-6.33
IGHV1OR15-1	A0A075B7D0	-0.0764	22.6	-0.258	7.97e-01	9.31e-01	-6.32
IGKV1-39	P01597;P04432	0.0429	21.2	0.249	8.04e-01	9.31e-01	-6.33
IGKV6D-21	A0A0A0MT36	-0.0662	19.5	-0.232	8.18e-01	9.31e-01	-6.33

IGLV3-21	P80748	-0.0505	19.6	-0.254	8.01e-01	9.31e-01	-6.33
IGLV3-9	A0A075B6K5	-0.0410	20.3	-0.260	7.96e-01	9.31e-01	-6.32
IGLV5-39	A0A0G2JS06	-0.0481	19.8	-0.256	7.99e-01	9.31e-01	-6.32
LCP1	P13796	-0.0324	17.6	-0.249	8.05e-01	9.31e-01	-6.33
MST1	P26927	-0.0369	19.8	-0.283	7.78e-01	9.31e-01	-6.32
PON1	P27169	-0.0274	21.9	-0.264	7.93e-01	9.31e-01	-6.32
QSOX1	O00391	0.0199	18.0	0.241	8.10e-01	9.31e-01	-6.33
SERPINA6	P08185	-0.0201	22.1	-0.228	8.20e-01	9.31e-01	-6.33
SELL	P14151	-0.0227	19.1	-0.220	8.26e-01	9.34e-01	-6.33
A0A0G2JRQ6	A0A0G2JRQ6	-0.0422	22.5	-0.208	8.36e-01	9.42e-01	-6.34
CPN1	P15169	-0.0182	20.5	-0.197	8.45e-01	9.48e-01	-6.34
CPN2	P22792	0.0169	21.6	0.189	8.51e-01	9.49e-01	-6.34
IGKV3D-20	A0A0C4DH25	0.0240	22.3	0.181	8.57e-01	9.49e-01	-6.34
IGLV1-40	P01703	0.0243	20.3	0.184	8.55e-01	9.49e-01	-6.34
LPA	P08519	0.0718	21.7	0.184	8.55e-01	9.49e-01	-6.34
C4B_2	P0C0L5	-0.0186	25.1	-0.173	8.63e-01	9.52e-01	-6.34
COL6A1	P12109	-0.0207	16.9	-0.160	8.74e-01	9.60e-01	-6.34
VWF	P04275	0.0247	20.0	0.157	8.76e-01	9.60e-01	-6.35
C5	P01031	0.00934	22.4	0.142	8.88e-01	9.70e-01	-6.35
APOA2	P02652	0.0128	26.6	0.133	8.94e-01	9.73e-01	-6.35
BTD	P43251	0.0104	18.0	0.112	9.11e-01	9.81e-01	-6.35
DSG1	Q02413	-0.0253	17.4	-0.108	9.15e-01	9.81e-01	-6.35
IGKV2-30	P06310	0.0210	18.1	0.119	9.06e-01	9.81e-01	-6.35
IGLV8-61	A0A075B6I0	0.0183	22.4	0.112	9.11e-01	9.81e-01	-6.35
SERPINA10	Q9UK55	-0.00869	19.1	-0.105	9.17e-01	9.81e-01	-6.35
CRISP3	P54108	0.0145	16.0	0.101	9.20e-01	9.81e-01	-6.35

APOH	P02749	-0.00812	23.1	-0.0684	9.46e-01	9.82e-01	-6.35
C4BPB	P20851	0.00614	20.6	0.0738	9.41e-01	9.82e-01	-6.35
CPB2	Q96IY4	0.00500	20.2	0.0602	9.52e-01	9.82e-01	-6.36
FCN3	O75636	0.00701	21.0	0.0574	9.54e-01	9.82e-01	-6.36
FGG	P02679	-0.00520	27.3	-0.0934	9.26e-01	9.82e-01	-6.35
IGHA1	P01876	-0.0124	28.3	-0.0727	9.42e-01	9.82e-01	-6.35
IGHV4-4	A0A075B6R2	-0.0106	18.0	-0.0765	9.39e-01	9.82e-01	-6.35
IGKV1-16	P04430	-0.0138	19.2	-0.0903	9.28e-01	9.82e-01	-6.35
IGKV2-40	A0A087WW87;P01614	-0.0113	17.8	-0.0589	9.53e-01	9.82e-01	-6.36
PLTP	P55058	-0.00539	19.3	-0.0610	9.52e-01	9.82e-01	-6.36
TNXB	P22105	-0.00955	18.0	-0.0854	9.32e-01	9.82e-01	-6.35
C7	P10643	-0.00371	21.0	-0.0479	9.62e-01	9.83e-01	-6.36
GLIPR2	Q9H4G4	-0.00416	17.3	-0.0490	9.61e-01	9.83e-01	-6.36
KLKB1	P03952	0.00289	22.8	0.0398	9.68e-01	9.87e-01	-6.36
APOC3	P02656	-0.00408	24.9	-0.0343	9.73e-01	9.88e-01	-6.36
C8G	P07360	0.00265	20.9	0.0282	9.78e-01	9.88e-01	-6.36
CFHR4	Q92496	0.00477	19.4	0.0267	9.79e-01	9.88e-01	-6.36
IGLV2-18	A0A075B6J9	-0.00124	18.2	-0.00597	9.95e-01	9.98e-01	-6.36
IGLV9-49	A0A0B4J1Y8	0.00223	19.5	0.00979	9.92e-01	9.98e-01	-6.36
ALB	P02768	0	32.0	0	1.00e+00	1.00e+00	-6.36

Table S4. Comparison of plasma protein abundance between type 1 diabetes with IR group versus type 1 diabetes without IR group, adjusted for sex as a covariate. Differential protein abundance was assessed using linear regression models (protein ~ group + sex), with the pairwise contrast IR+ vs IR−. P-values were adjusted for multiple testing using the Benjamini–Hochberg false discovery rate (FDR), with significance defined as $p < 0.05$.

Protein (gene name)	UniProtID	logFC	AveExpr	t	P.Value	adj.P.Val	B
ABCB9	Q9NP78	-1.38	19.8	4.15	1.09e-04	3.47e-02	1.16
VWF	P04275	0.73	20.0	-3.92	2.32e-04	3.71e-02	0.482
CAMP	P49913	-1	18.6	3.49	9.25e-04	7.40e-02	-0.747
PPBP	P02775	0.76	19.3	-3.50	8.96e-04	7.40e-02	-0.719
CFH	P08603	0.25	23.6	-3.15	2.58e-03	1.65e-01	-1.65
IGLV1-51	P01701	-0.33	21.5	3.06	3.32e-03	1.77e-01	-1.87
IGLV8-61	A0A075B6I0	-0.56	22.4	2.89	5.37e-03	2.45e-01	-2.29
IGHV6-1	A0A0B4J1U7	-0.36	23.2	2.84	6.23e-03	2.49e-01	-2.41
FGA	P02671	0.18	26.9	-2.69	9.17e-03	2.75e-01	-2.75
FGG	P02679	0.18	27.3	-2.68	9.45e-03	2.75e-01	-2.77
PROS1	P07225	0.21	22.0	-2.75	7.83e-03	2.75e-01	-2.61
BTD	P43251	0.28	18.0	-2.50	1.51e-02	2.77e-01	-3.17
C1RL	Q9NZP8	0.26	18.7	-2.60	1.19e-02	2.77e-01	-2.97
CASP14	P31944	-0.7	16.5	2.44	1.77e-02	2.77e-01	-3.30
CETP	P11597	-0.32	19.6	2.42	1.86e-02	2.77e-01	-3.35
CFB	P00751	0.22	24.4	-2.54	1.37e-02	2.77e-01	-3.09
IGHV2-5	P01817	0.88	17.2	-2.46	1.70e-02	2.77e-01	-3.27
IGHV3-64D	A0A0J9YX35	-0.45	21.9	2.42	1.88e-02	2.77e-01	-3.36
IGKV2D-24	A0A075B6R9	-0.37	22.4	2.41	1.91e-02	2.77e-01	-3.37
IGLC3	P0DOY3	-0.29	26.9	2.61	1.14e-02	2.77e-01	-2.93

LBP	P18428	0.4	19.6	-2.54	1.37e-02	2.77e-01	-3.09
SERPINA1	P01009	0.25	29.6	-2.48	1.61e-02	2.77e-01	-3.22
IGHV2-70D	A0A0C4DH43	1.24	20.8	-2.25	2.81e-02	3.21e-01	-3.69
IGHV3-15	A0A0B4J1V0	-0.34	23.2	2.26	2.74e-02	3.21e-01	-3.67
IGHV3-38-3	P0DTE1	-0.42	18.5	2.31	2.44e-02	3.21e-01	-3.57
IGKV4-1	P06312	-0.48	22.7	2.24	2.91e-02	3.21e-01	-3.72
IGLV1-40	P01703	-0.35	20.3	2.25	2.83e-02	3.21e-01	-3.70
MAN1A1	P33908	0.18	18.2	-2.24	2.88e-02	3.21e-01	-3.71
PLTP	P55058	-0.24	19.3	2.25	2.81e-02	3.21e-01	-3.69
IGKC	P01834	-0.26	24.6	2.22	3.05e-02	3.25e-01	-3.76
CPB2	Q96IY4	0.21	20.2	-2.15	3.53e-02	3.64e-01	-3.88
C5	P01031	0.17	22.4	-2.11	3.94e-02	3.90e-01	-3.97
GC	P02774	0.17	24.0	-2.10	4.02e-02	3.90e-01	-3.99
C4B_2	P0C0L5	0.26	25.1	-2.08	4.22e-02	3.92e-01	-4.02
IGHG4	P01861	-0.19	29.9	2.01	4.90e-02	3.92e-01	-4.14
IGHV1-2	P23083	-0.3	18.4	2.04	4.63e-02	3.92e-01	-4.10
IGHV4-28	A0A0C4DH34	-0.37	20.3	2.01	4.89e-02	3.92e-01	-4.14
KLKB1	P03952	0.17	22.8	-2.03	4.72e-02	3.92e-01	-4.11
LRG1	P02750	0.29	22.8	-2.05	4.47e-02	3.92e-01	-4.07
SERPINA10	Q9UK55	0.2	19.1	-2.06	4.43e-02	3.92e-01	-4.06
ALDOA	P04075	-0.53	17.5	1.97	5.39e-02	4.09e-01	-4.22
C1S	P09871	0.13	23.7	-1.96	5.49e-02	4.09e-01	-4.24
C4BPA	P04003	0.15	22.6	-1.93	5.87e-02	4.09e-01	-4.29
IGFALS	P35858	0.22	21.3	-1.93	5.87e-02	4.09e-01	-4.29
IGHV3-35	A0A0C4DH35	-0.28	19.0	1.93	5.88e-02	4.09e-01	-4.29
NID1	P14543	0.24	16.3	-1.93	5.88e-02	4.09e-01	-4.29

AFM	P43652	0.19	22.5	-1.88	6.56e-02	4.20e-01	-4.38
C4BPB	P20851	0.19	20.6	-1.88	6.46e-02	4.20e-01	-4.37
IGHV3-13	P01766	-0.27	19.7	1.89	6.35e-02	4.20e-01	-4.35
IGHV3-9	P01782	-0.34	18.4	1.88	6.56e-02	4.20e-01	-4.38
IGHV5-51	A0A0C4DH38	-0.36	20.5	1.86	6.80e-02	4.25e-01	-4.41
IGKV1-27	A0A075B6S5	-0.33	20.5	1.83	7.27e-02	4.25e-01	-4.46
IGLV2-11	P01706	-0.24	22.5	1.83	7.30e-02	4.25e-01	-4.46
PIP	P12273	0.36	16.9	-1.83	7.22e-02	4.25e-01	-4.45
SERPINA5	P05154	0.17	20.7	-1.85	6.94e-02	4.25e-01	-4.42
CNDP1	Q96KN2	0.29	19.3	-1.80	7.64e-02	4.37e-01	-4.50
F10	P00742	0.14	20.7	-1.79	7.86e-02	4.41e-01	-4.52
APOC4	P55056	0.25	19.1	-1.78	8.05e-02	4.44e-01	-4.54
C3	P01024	0.13	27.4	-1.76	8.44e-02	4.50e-01	-4.58
PRG4	Q92954	0.27	20.0	-1.76	8.30e-02	4.50e-01	-4.56
SERPINA7	P05543	0.16	20.8	-1.74	8.78e-02	4.57e-01	-4.61
SHBG	P04278	-0.38	19.1	1.73	8.86e-02	4.57e-01	-4.61
CFI	P05156	0.15	22.3	-1.72	9.12e-02	4.62e-01	-4.64
CPN2	P22792	0.18	21.6	-1.70	9.47e-02	4.62e-01	-4.67
IGKV2-40	A0A087WW87;P01614	0.38	17.8	-1.68	9.80e-02	4.62e-01	-4.69
IGKV3D-15	A0A087WSY6	-0.26	23.2	1.69	9.68e-02	4.62e-01	-4.68
LILRA3	Q8N6C8	0.32	16.2	-1.68	9.81e-02	4.62e-01	-4.69
MASP1	P48740	0.2	17.6	-1.70	9.35e-02	4.62e-01	-4.66
C6	P13671	0.15	20.8	-1.67	9.97e-02	4.62e-01	-4.71
CFHR4	Q92496	0.35	19.4	-1.66	1.03e-01	4.67e-01	-4.73
SAA2	P0DJI9	0.92	20.1	-1.65	1.04e-01	4.67e-01	-4.74

IGHV3-38	A0A0C4DH36	-0.3	18.5	1.62	1.11e-01	4.94e-01	-4.79
IGLC7	A0M8Q6	-0.24	23.6	1.60	1.14e-01	5.00e-01	-4.81
MST1	P26927	-0.25	19.8	1.60	1.16e-01	5.01e-01	-4.82
IGHV3-7	P01780	-0.17	24.0	1.59	1.18e-01	5.02e-01	-4.83
APOF	Q13790	-0.19	19.9	1.46	1.50e-01	5.07e-01	-5.01
B4GALT1	P15291	0.29	15.0	-1.55	1.26e-01	5.07e-01	-4.89
BCHE	P06276	0.15	19.6	-1.47	1.47e-01	5.07e-01	-5.00
C2	P06681	0.13	21.0	-1.46	1.51e-01	5.07e-01	-5.02
C9	P02748	0.18	23.4	-1.54	1.28e-01	5.07e-01	-4.90
CRISP3	P54108	0.25	16.0	-1.47	1.48e-01	5.07e-01	-5.00
F13B	P05160	0.15	20.1	-1.52	1.34e-01	5.07e-01	-4.93
IGKV1-12	A0A0C4DH73; P01611	-0.38	18.8	1.51	1.37e-01	5.07e-01	-4.95
IGLV5-39	A0A0G2JS06	-0.34	19.8	1.52	1.33e-01	5.07e-01	-4.92
IGLV7-46	A0A075B6I9	-0.27	23.4	1.53	1.30e-01	5.07e-01	-4.91
LRP1	Q07954	0.24	15.6	-1.48	1.44e-01	5.07e-01	-4.98
LYZ	P61626	0.19	20.0	-1.53	1.32e-01	5.07e-01	-4.92
MCAM	P43121	-0.17	17.0	1.54	1.30e-01	5.07e-01	-4.91
ORM2	P19652	0.17	23.3	-1.49	1.40e-01	5.07e-01	-4.96
PI16	Q6UXB8	-0.21	18.4	1.46	1.50e-01	5.07e-01	-5.01
PLXDC2	Q6UX71	0.2	17.0	-1.51	1.37e-01	5.07e-01	-4.94
SERPINA6	P08185	0.16	22.1	-1.49	1.42e-01	5.07e-01	-4.97
SERPIND1	P05546	0.17	23.2	-1.49	1.41e-01	5.07e-01	-4.97
VASN	Q6EMK4	0.13	19.0	-1.57	1.22e-01	5.07e-01	-4.86
VTN	P04004	0.1	24.3	-1.46	1.48e-01	5.07e-01	-5.01
C8A	P07357	0.13	21.6	-1.42	1.60e-01	5.18e-01	-5.06

F9	P00740	0.14	19.7	-1.43	1.59e-01	5.18e-01	-5.06
HABP2	Q14520	0.15	18.9	-1.40	1.67e-01	5.18e-01	-5.09
IGKJ1	A0A0A0MT89	-0.29	18.4	1.41	1.65e-01	5.18e-01	-5.08
IGKV1-8	A0A0C4DH67; A0A0C4DH69	-0.3	20.5	1.40	1.67e-01	5.18e-01	-5.09
IGKV3-7	A0A075B6H7	-0.3	25.2	1.39	1.68e-01	5.18e-01	-5.10
IGLV5-37	A0A075B6J1	-0.48	17.6	1.44	1.57e-01	5.18e-01	-5.04
MASP2	O00187	0.21	20.6	-1.41	1.65e-01	5.18e-01	-5.08
SERPING1	P05155	0.13	24.4	-1.40	1.67e-01	5.18e-01	-5.09
APOL1	O14791	0.18	22.2	-1.38	1.72e-01	5.24e-01	-5.11
C4A	P0C0L4	0.28	22.8	-1.37	1.76e-01	5.27e-01	-5.13
DBH	P09172	0.32	18.2	-1.37	1.75e-01	5.27e-01	-5.12
IGLV2-14	P01704	-0.32	18.4	1.36	1.78e-01	5.29e-01	-5.14
F11	P03951	0.15	20.1	-1.35	1.83e-01	5.35e-01	-5.16
IGHV3OR16-9	A0A0B4J2B5	-0.2	25.4	1.34	1.84e-01	5.35e-01	-5.16
IGLV7-43	P04211	-0.23	18.7	1.34	1.86e-01	5.36e-01	-5.17
AZGP1	P25311	0.19	21.0	-1.33	1.90e-01	5.42e-01	-5.18
HPR	P00739	0.2	21.9	-1.30	1.97e-01	5.59e-01	-5.21
C1R	P00736	0.1	21.4	-1.30	1.99e-01	5.59e-01	-5.22
IGF2R	P11717	-0.16	16.2	1.27	2.08e-01	5.79e-01	-5.25
HGFAC	Q04756	-0.18	22.0	1.26	2.14e-01	5.91e-01	-5.27
IGHV3-43	A0A0B4J1X8	-0.16	20.4	1.25	2.17e-01	5.95e-01	-5.28
FN1	P02751	0.19	23.3	-1.23	2.22e-01	6.02e-01	-5.29
PTGDS	P41222	-0.19	18.2	1.22	2.26e-01	6.07e-01	-5.30
FGB	P02675	0.11	25.9	-1.22	2.28e-01	6.07e-01	-5.31
APOC2	P02655	0.16	22.7	-1.19	2.40e-01	6.07e-01	-5.35

© Thieme. All rights reserved.

F13A1	P00488	0.1	20.5	-1.19	2.39e-01	6.07e-01	-5.34
HSPG2	P98160	-0.14	16.3	1.18	2.41e-01	6.07e-01	-5.35
IGHV1-46	P01743	-0.2	19.4	1.19	2.38e-01	6.07e-01	-5.34
IGKV1-17	P01599	-0.16	21.7	1.20	2.35e-01	6.07e-01	-5.33
NRP1	O14786	-0.14	16.9	1.21	2.33e-01	6.07e-01	-5.33
QSOX1	O00391	0.12	18.0	-1.21	2.32e-01	6.07e-01	-5.32
A2M	P01023	-0.15	27.3	1.16	2.49e-01	6.24e-01	-5.37
LGALS3BP	Q08380	0.19	19.4	-1.16	2.52e-01	6.25e-01	-5.38
AMBP	P02760	0.09	23.1	-1.15	2.55e-01	6.25e-01	-5.39
TFRC	P02786	0.13	18.9	-1.15	2.56e-01	6.25e-01	-5.39
FETUB	Q9UGM5	0.19	20.1	-1.14	2.61e-01	6.28e-01	-5.40
SERPINF1	P36955	0.1	21.2	-1.14	2.59e-01	6.28e-01	-5.40
IGKV1-37	A0A075B6S9;P0DSN7	-0.29	17.8	1.13	2.64e-01	6.29e-01	-5.41
ORM1	P02763	0.14	25.2	-1.12	2.66e-01	6.30e-01	-5.42
LPA	P08519	0.51	21.7	-1.10	2.77e-01	6.46e-01	-5.44
SERPINF2	P08697	0.08	21.7	-1.10	2.75e-01	6.46e-01	-5.44
APCS	P02743	0.22	17.3	-1.09	2.81e-01	6.46e-01	-5.45
IGHG2	P01859	-0.16	28.3	1.09	2.81e-01	6.46e-01	-5.45
MBL2	P11226	0.29	19.2	-1.08	2.83e-01	6.47e-01	-5.46
APOD	P05090	-0.1	23.1	1.07	2.87e-01	6.51e-01	-5.47
PROC	P04070	-0.13	19.9	1.07	2.89e-01	6.51e-01	-5.47
ART3	Q13508	-0.16	15.6	1.05	2.98e-01	6.63e-01	-5.49
B2M	P61769	0.16	18.6	-1.05	2.98e-01	6.63e-01	-5.49
C8B	P07358	0.11	20.0	-1.05	3.00e-01	6.63e-01	-5.49
CDH13	P55290	-0.12	17.4	1.01	3.15e-01	6.86e-01	-5.53

IGKV1-13	A0A0B4J2D9;P0DP09	-0.36	19.1	1.02	3.13e-01	6.86e-01	-5.52
APOC1	P02654	-0.13	22.3	0.991	3.26e-01	6.94e-01	-5.55
FCGBP	Q9Y6R7	0.14	18.9	-0.983	3.30e-01	6.94e-01	-5.55
IGKV1-6	A0A0C4DH72	-0.19	20.8	0.993	3.25e-01	6.94e-01	-5.54
PRDX2	P32119	0.24	17.3	-0.987	3.28e-01	6.94e-01	-5.55
SERPINA3	P01011	0.08	24.9	-0.987	3.27e-01	6.94e-01	-5.55
ICOSLG	O75144	-0.12	17.1	0.956	3.43e-01	7.17e-01	-5.58
IGHV3-49	A0A0A0MS15	-0.11	24.2	0.937	3.52e-01	7.32e-01	-5.60
VCAM1	P19320	0.11	18.7	-0.931	3.56e-01	7.35e-01	-5.60
COL6A3	P12111	0.07	17.2	-0.899	3.72e-01	7.64e-01	-5.63
IGHV1-24	A0A0C4DH33	-0.25	18.6	0.885	3.80e-01	7.74e-01	-5.64
ANTXR1	Q9H6X2	0.11	16.1	-0.843	4.02e-01	7.78e-01	-5.67
FCN2	Q15485	-0.17	18.0	0.837	4.06e-01	7.78e-01	-5.68
IGHA1	P01876	-0.17	28.3	0.843	4.03e-01	7.78e-01	-5.67
IGKV1-33	P01593;P01594	-0.16	20.7	0.838	4.05e-01	7.78e-01	-5.68
IGLV1-47	P01700	-0.12	23.4	0.845	4.01e-01	7.78e-01	-5.67
IGLV9-49	A0A0B4J1Y8	0.23	19.5	-0.848	4.00e-01	7.78e-01	-5.67
ITIH3	Q06033	0.11	21.2	-0.875	3.85e-01	7.78e-01	-5.65
ITIH4	Q14624	0.06	25.4	-0.841	4.04e-01	7.78e-01	-5.68
TGFBI	Q15582	0.09	18.8	-0.859	3.94e-01	7.78e-01	-5.66
TNXB	P22105	-0.12	18.0	0.869	3.88e-01	7.78e-01	-5.65
IGHV3-64	A0A075B6Q5	0.21	18.4	-0.833	4.08e-01	7.78e-01	-5.68
GPX3	P22352	-0.09	20.2	0.821	4.15e-01	7.80e-01	-5.69
IGHG3	P01860	0.1	28.9	-0.826	4.12e-01	7.80e-01	-5.69

IGHV3OR16-12	A0A075B7B8	-0.15	23.0	0.818	4.17e-01	7.80e-01	-5.69
CLU	P10909	0.06	24.5	-0.810	4.21e-01	7.83e-01	-5.70
FBLN5	Q9UBX5	0.11	16.0	-0.804	4.25e-01	7.83e-01	-5.70
PIGR	P01833	0.13	17.2	-0.802	4.26e-01	7.83e-01	-5.71
IGHV1OR15-1	A0A075B7D0	-0.28	22.6	0.793	4.31e-01	7.88e-01	-5.71
C1QC	P02747	0.06	24.0	-0.779	4.39e-01	7.95e-01	-5.72
SAA4	P35542	0.1	22.9	-0.778	4.40e-01	7.95e-01	-5.72
RBP4	P02753	-0.12	22.7	0.773	4.43e-01	7.96e-01	-5.73
ANGPTL6	Q8NI99	-0.14	15.8	0.764	4.48e-01	8.01e-01	-5.73
IGHD	P01880	-0.24	20.4	0.757	4.52e-01	8.04e-01	-5.74
IGKV1-5	P01602	-0.14	24.0	0.726	4.71e-01	8.16e-01	-5.76
IGKV1D-16	P01601	-0.18	17.9	0.720	4.74e-01	8.16e-01	-5.77
IGKV2-24	A0A0C4DH68	-0.16	18.8	0.727	4.70e-01	8.16e-01	-5.76
IGLV3-21	P80748	-0.18	19.6	0.741	4.62e-01	8.16e-01	-5.75
PROCR	Q9UNN8	-0.13	18.1	0.734	4.66e-01	8.16e-01	-5.76
SOD3	P08294	-0.16	17.7	0.721	4.74e-01	8.16e-01	-5.76
AGT	P01019	0.11	23.2	-0.685	4.96e-01	8.17e-01	-5.79
C1QB	P02746	0.08	22.2	-0.694	4.90e-01	8.17e-01	-5.78
CPN1	P15169	0.07	20.5	-0.678	5.00e-01	8.17e-01	-5.79
GLIPR2	Q9H4G4	-0.07	17.3	0.694	4.91e-01	8.17e-01	-5.78
GP1BA	P07359	0.09	18.8	-0.687	4.95e-01	8.17e-01	-5.79
HSPA5	P11021	0.08	17.5	-0.689	4.94e-01	8.17e-01	-5.79
IGHV1-18	A0A0C4DH31	-0.11	19.7	0.692	4.92e-01	8.17e-01	-5.78
IGKV6-21	A0A0C4DH24	-0.13	20.4	0.711	4.80e-01	8.17e-01	-5.77
IGKV6D-21	A0A0A0MT36	0.23	19.5	-0.681	4.98e-01	8.17e-01	-5.79
RNASE4	P34096	-0.11	16.0	0.701	4.86e-01	8.17e-01	-5.78

IGHG1	P01857	-0.07	28.7	0.667	5.07e-01	8.24e-01	-5.80
HBD	P02042	0.18	18.0	-0.661	5.11e-01	8.26e-01	-5.80
APMAP	Q9HDC9	-0.13	18.8	0.627	5.33e-01	8.33e-01	-5.82
CLEC3B	P05452	0.08	19.6	-0.632	5.30e-01	8.33e-01	-5.82
IGKV3D-7	A0A0C4DH55	0.21	17.7	-0.635	5.28e-01	8.33e-01	-5.82
IGLL1	P15814	0.16	17.4	-0.626	5.34e-01	8.33e-01	-5.83
PROZ	P22891	-0.12	20.0	0.630	5.31e-01	8.33e-01	-5.82
SELENOP	P49908	0.07	20.1	-0.632	5.30e-01	8.33e-01	-5.82
TTR	P02766	0.06	25.6	-0.640	5.25e-01	8.33e-01	-5.82
FBLN1	P23142	0.07	20.2	-0.621	5.37e-01	8.34e-01	-5.83
IGHV1-45	A0A0A0MS14	-0.22	18.1	0.613	5.42e-01	8.38e-01	-5.83
CA1	P00915	0.17	18.3	-0.603	5.49e-01	8.44e-01	-5.84
IGKV3-11	A0A0A0MRZ8; P04433	-0.07	23.6	0.599	5.52e-01	8.44e-01	-5.84
APOE	P02649	0.06	23.9	-0.591	5.57e-01	8.46e-01	-5.85
LTF	P02788	-0.1	17.6	0.589	5.58e-01	8.46e-01	-5.85
IGLV1-36	A0A0B4J1U3	-0.15	19.0	0.584	5.61e-01	8.47e-01	-5.85
ALCAM	Q13740	0.1	15.1	-0.565	5.74e-01	8.51e-01	-5.86
IGHV1-3	A0A0C4DH29	-0.13	18.6	0.567	5.73e-01	8.51e-01	-5.86
IGHV4-34	P06331	-0.07	23.2	0.564	5.75e-01	8.51e-01	-5.86
IGLV3-10	A0A075B6K4	-0.16	20.4	0.559	5.78e-01	8.51e-01	-5.86
PKM	P14618	-0.12	16.4	0.556	5.80e-01	8.51e-01	-5.86
SERPINA4	P29622	0.05	21.6	-0.557	5.80e-01	8.51e-01	-5.86
PON3	Q15166	-0.06	18.9	0.552	5.83e-01	8.52e-01	-5.87
IGKV1-39	P01597;P04432	-0.11	21.2	0.539	5.92e-01	8.61e-01	-5.87

© Thieme. All rights reserved.

APOA1	P02647	-0.05	28.2	0.503	6.17e-01	8.76e-01	-5.89
APOA2	P02652	0.06	26.6	-0.490	6.26e-01	8.76e-01	-5.90
APOC3	P02656	-0.07	24.9	0.470	6.40e-01	8.76e-01	-5.91
CD5L	O43866	-0.14	22.1	0.478	6.35e-01	8.76e-01	-5.90
CDH5	P33151	-0.05	19.5	0.502	6.18e-01	8.76e-01	-5.89
DSG1	Q02413	-0.13	17.4	0.472	6.39e-01	8.76e-01	-5.91
F2	P00734	0.04	22.6	-0.508	6.14e-01	8.76e-01	-5.89
HRG	P04196	-0.05	24.0	0.473	6.38e-01	8.76e-01	-5.91
IGKV3D-20	A0A0C4DH25	-0.08	22.3	0.507	6.14e-01	8.76e-01	-5.89
IGLL5	B9A064	-0.1	25.3	0.515	6.09e-01	8.76e-01	-5.89
LDHA	P00338	0.09	16.9	-0.488	6.28e-01	8.76e-01	-5.90
LUM	P51884	0.04	22.9	-0.476	6.36e-01	8.76e-01	-5.90
PRSS3	P35030	-0.11	18.5	0.496	6.22e-01	8.76e-01	-5.89
SELL	P14151	0.06	19.1	-0.473	6.38e-01	8.76e-01	-5.91
IGFBP3	P17936	0.06	20.1	-0.461	6.47e-01	8.80e-01	-5.91
TF	P02787	-0.04	27.8	0.458	6.49e-01	8.80e-01	-5.91
ITIH2	P19823	0.04	24.1	-0.453	6.52e-01	8.80e-01	-5.91
GSN	P06396	-0.04	22.7	0.445	6.58e-01	8.81e-01	-5.92
IGHV3-74	A0A0B4J1X5	-0.07	21.7	0.446	6.58e-01	8.81e-01	-5.92
AHSG	P02765	-0.04	25.3	0.429	6.69e-01	8.85e-01	-5.92
HSPA6	P17066;P48741	0.09	16.8	-0.434	6.66e-01	8.85e-01	-5.92
PCYOX1	Q9UHG3	-0.06	19.1	0.432	6.67e-01	8.85e-01	-5.92
HP	P00738	0.07	27.9	-0.422	6.75e-01	8.89e-01	-5.93
SPP2	Q13103	0.08	18.5	-0.414	6.80e-01	8.92e-01	-5.93
ECM1	Q16610	0.04	21.0	-0.405	6.87e-01	8.98e-01	-5.93

IGLV3-9	A0A075B6K5	-0.07	20.3	0.396	6.94e-01	9.03e-01	-5.94
APOB	P04114	0.04	24.4	-0.378	7.07e-01	9.05e-01	-5.94
CD14	P08571	0.05	17.5	-0.382	7.04e-01	9.05e-01	-5.94
FCN3	O75636	0.05	21.0	-0.374	7.10e-01	9.05e-01	-5.95
HBA2	P69905	0.1	23.5	-0.380	7.05e-01	9.05e-01	-5.94
HPX	P02790	-0.03	24.5	0.374	7.10e-01	9.05e-01	-5.95
IGLV5-45	A0A087WSX0	0.09	17.6	-0.365	7.16e-01	9.09e-01	-5.95
C8G	P07360	0.04	20.9	-0.356	7.23e-01	9.11e-01	-5.95
CTSD	P07339	-0.09	16.7	0.352	7.26e-01	9.11e-01	-5.95
IGHA2	P01877	0.12	22.1	-0.355	7.24e-01	9.11e-01	-5.95
A0A0J9YY99	A0A0J9YY99	-0.05	22.0	0.336	7.38e-01	9.22e-01	-5.96
IGHV1-69	P01742	-0.09	17.1	0.329	7.43e-01	9.26e-01	-5.96
F5	P12259	0.03	19.7	-0.303	7.63e-01	9.43e-01	-5.97
PGLYRP2	Q96PD5	-0.03	22.0	0.302	7.64e-01	9.43e-01	-5.97
JCHAIN	P01591	0.05	24.2	-0.295	7.69e-01	9.46e-01	-5.97
Biognosys	Biognosys	-0.04	25.6	0.290	7.73e-01	9.48e-01	-5.97
A0A0G2JRQ6	A0A0G2JRQ6	0.06	22.5	-0.239	8.12e-01	9.51e-01	-5.98
CD44	P16070	0.02	19.5	-0.230	8.19e-01	9.51e-01	-5.99
CFHR5	Q9BXR6	0.03	19.1	-0.241	8.11e-01	9.51e-01	-5.98
COL6A1	P12109	-0.04	16.9	0.234	8.16e-01	9.51e-01	-5.99
CPQ	Q9Y646	-0.04	15.4	0.228	8.20e-01	9.51e-01	-5.99
DCD	P81605	0.06	17.9	-0.240	8.12e-01	9.51e-01	-5.98
F7	P08709	-0.04	18.1	0.261	7.95e-01	9.51e-01	-5.98
GAPDH	P04406	0.06	17.6	-0.229	8.20e-01	9.51e-01	-5.99
GPLD1	P80108	-0.03	21.0	0.246	8.06e-01	9.51e-01	-5.98
IGHV3OR15-7	A0A075B7D8	0.06	15.7	-0.261	7.95e-01	9.51e-01	-5.98

IGKV2-30	P06310	0.06	18.1	-0.278	7.82e-01	9.51e-01	-5.98
IGLV2-18	A0A075B6J9	0.06	18.2	-0.236	8.14e-01	9.51e-01	-5.99
IGLV3-27	P01718	0.04	20.3	-0.244	8.08e-01	9.51e-01	-5.98
KNG1	P01042	0.02	25.1	-0.254	8.00e-01	9.51e-01	-5.98
PRR4	Q16378	0.07	16.3	-0.274	7.85e-01	9.51e-01	-5.98
CFD	P00746	-0.03	19.0	0.204	8.39e-01	9.59e-01	-5.99
IGHV4-4	A0A075B6R2	-0.03	18.0	0.208	8.36e-01	9.59e-01	-5.99
ITIH1	P19827	-0.02	23.8	0.204	8.39e-01	9.59e-01	-5.99
PTPRJ	Q12913	-0.03	17.3	0.211	8.34e-01	9.59e-01	-5.99
A1BG	P04217	0.01	24.7	-0.192	8.48e-01	9.65e-01	-5.99
APOA4	P06727	-0.02	24.7	0.190	8.50e-01	9.65e-01	-6.00
HSP90B1	P14625	0.03	16.5	-0.174	8.63e-01	9.75e-01	-6.00
IGLV2-8	P01709	-0.04	19.8	0.164	8.71e-01	9.81e-01	-6.00
MMP2	P08253	0.02	15.8	-0.151	8.81e-01	9.89e-01	-6.00
ADAMTS13	Q76LX8	0.01	16.3	-0.146	8.84e-01	9.89e-01	-6.00
CST3	P01034	-0.02	20.2	0.143	8.87e-01	9.89e-01	-6.00
EFEMP1	Q12805	0.01	18.5	-0.131	8.97e-01	9.89e-01	-6.00
IGKV3-20	P01619	-0.03	24.7	0.131	8.96e-01	9.89e-01	-6.00
LCAT	P04180	-0.01	20.1	0.133	8.94e-01	9.89e-01	-6.00
APOM	O95445	-0.01	22.7	0.121	9.04e-01	9.90e-01	-6.01
F12	P00748	0.02	23.2	-0.118	9.07e-01	9.90e-01	-6.01
FCGR3A	P08637	0.02	18.0	-0.107	9.15e-01	9.90e-01	-6.01
FCGR3B	O75015	0.02	17.4	-0.107	9.16e-01	9.90e-01	-6.01
IGHM	P01871	0.02	25.8	-0.113	9.10e-01	9.90e-01	-6.01
THBS4	P35443	-0.02	16.3	0.121	9.04e-01	9.90e-01	-6.01
APOH	P02749	-0.01	23.1	0.0998	9.21e-01	9.92e-01	-6.01

COLEC11	Q9BWP8	-0.01	16.6	0.0869	9.31e-01	1.00e+00	-6.01
PON1	P27169	-0.01	21.9	0.0832	9.34e-01	1.00e+00	-6.01
ALB	P02768	0	32.0	0	1.00e+00	1.00e+00	-6.01
ATRN	O75882	0	19.3	0.0167	9.87e-01	1.00e+00	-6.01
C7	P10643	0	21.0	-0.00883	9.93e-01	1.00e+00	-6.01
CFHR1	Q03591	-0.01	19.0	0.0416	9.67e-01	1.00e+00	-6.01
CHL1	O00533	0	18.7	0.0322	9.74e-01	1.00e+00	-6.01
COMP	P49747	-0.01	19.1	0.0497	9.61e-01	1.00e+00	-6.01
CP	P00450	0	24.8	-0.0171	9.86e-01	1.00e+00	-6.01
CRTAC1	Q9NQ79	-0.01	25.5	0.0657	9.48e-01	1.00e+00	-6.01
HBB	P68871	0.01	22.8	-0.0352	9.72e-01	1.00e+00	-6.01
IGHV2-26	A0A0B4J1V2	0	20.9	0.0134	9.89e-01	1.00e+00	-6.01
IGHV3-72	A0A0B4J1Y9	0	16.8	0.00157	9.99e-01	1.00e+00	-6.01
IGKV1-16	P04430	0	19.2	-0.00498	9.96e-01	1.00e+00	-6.01
IGKV2D-30	A0A075B6S6	-0.01	23.8	0.0657	9.48e-01	1.00e+00	-6.01
IGLV3-19	P01714	0.01	17.4	-0.0333	9.74e-01	1.00e+00	-6.01
IL1RAP	Q9NPH3	0	18.0	-0.0139	9.89e-01	1.00e+00	-6.01
LCP1	P13796	-0.01	17.6	0.0523	9.58e-01	1.00e+00	-6.01
LYVE1	Q9Y5Y7	0.01	19.1	-0.0421	9.67e-01	1.00e+00	-6.01
PLG	P00747	0	23.5	-0.0163	9.87e-01	1.00e+00	-6.01
PVR	P15151	0.01	17.8	-0.0531	9.58e-01	1.00e+00	-6.01
RARRES2	Q99969	0	17.2	-0.0142	9.89e-01	1.00e+00	-6.01
SERPINC1	P01008	0	24.7	0.0281	9.78e-01	1.00e+00	-6.01

© Thieme. All rights reserved.

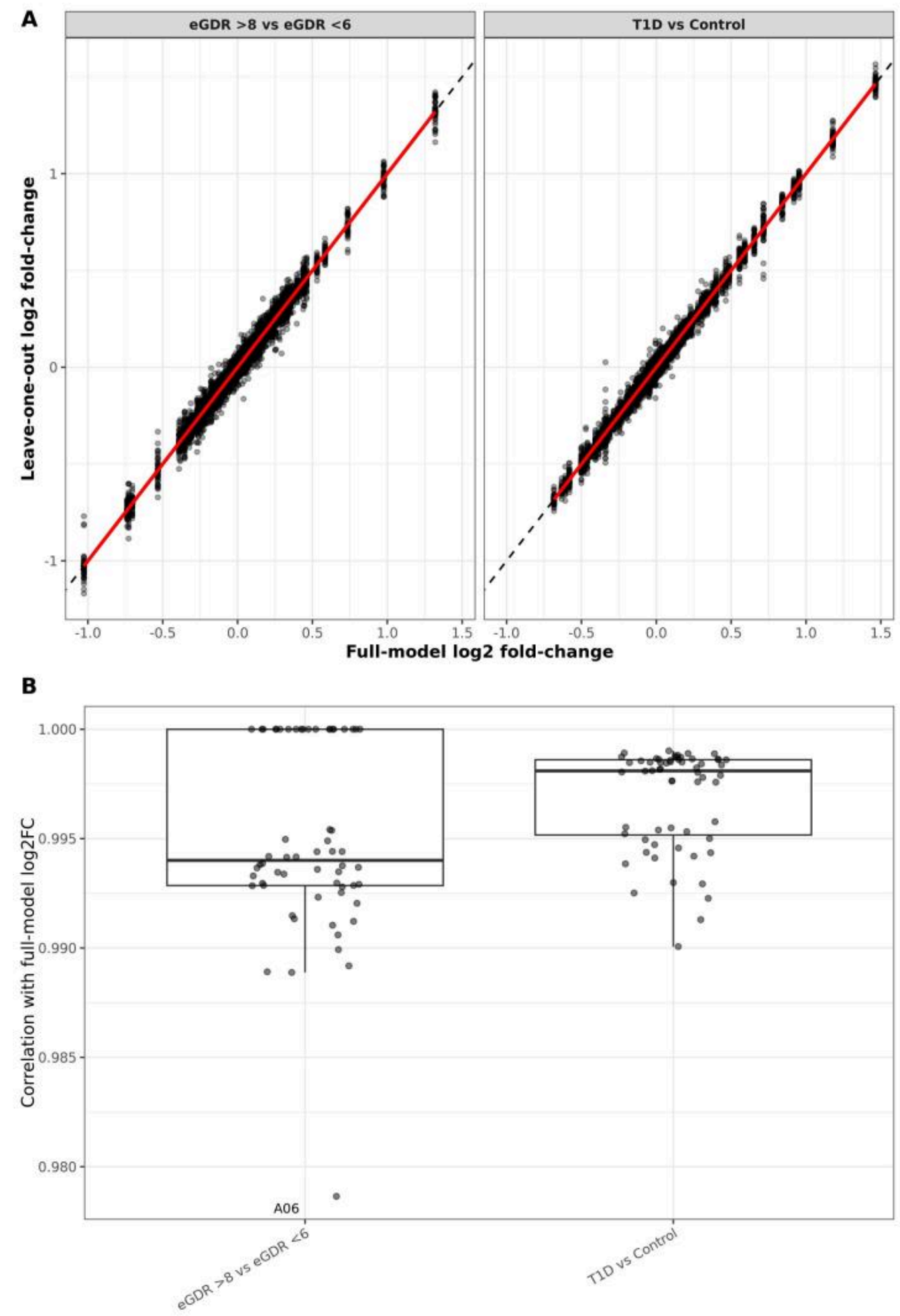

Figure S1 Leave-one-out sensitivity analysis of limma-derived protein abundance estimates. Full-model log_2 fold-change estimates (**A**) were compared with leave-one-out log_2 fold-change estimates for each protein across the contrasts. The red solid regression line represents the fitted linear relationship. The dashed diagonal line represents perfect agreement between the full model and leave-one-out estimates. High concordance with the diagonal indicates that differential abundance estimates were robust to the iterative removal of individual samples. Sample-level influence (**B**) in leave-one-out sensitivity analysis. For each contrast, the correlation between full-model log_2 fold-change estimates and leave-one-out log_2 fold-change estimates was calculated after removing one sample at a time. Each point represents one leave-one-out model, and boxplots summarise the distribution of correlations across samples. Lower correlations indicate samples with greater influence on the global protein abundance estimates. Labelled points indicate potential outlier samples based on the interquartile range criterion.

Table S5. Plasma protein correlations with haemostatic parameters. Pearson correlation coefficients were calculated to evaluate associations between plasma protein levels and haemostatic parameters, with FDR correction applied to corresponding p-values.

var1	var2	cor	pvalue	FDR	qvalue
FGG	Max_absorbance	0.601856	3.99E-05	0.056099	0.042322
PPBP	eGDRWC	-0.59552	5.04E-05	0.056099	0.042322
F12	Lag_time_sec	-0.5763	9.96E-05	0.073877	0.055734
C4BPA	HbA1c	0.566896	0.000137	0.076134	0.057437
C8B	Clot_lysis_area	0.525336	0.0005	0.197396	0.14892
CFB	eGDRWC	-0.52321	0.000532	0.197396	0.14892
C1RL	Max_absorbance	0.517537	0.000626	0.199157	0.150248
APOC4	HbA1c	0.510127	0.000772	0.214753	0.162014
C8A	HbA1c	0.499558	0.001031	0.255094	0.192448
HP	Max_absorbance	0.489407	0.001351	0.3007	0.226854
IGLV2-11	HbA1c	-0.48342	0.001578	0.319307	0.240891
HP	Clot_lysis_area	0.466419	0.002416	0.336175	0.253617
IGHV2-70D	Lag_time_sec	-0.47362	0.002023	0.336175	0.253617
LBP	Max_absorbance	0.470454	0.002188	0.336175	0.253617
PROS1	eGDRWC	-0.46813	0.002317	0.336175	0.253617
ANGPTL6	Lag_time_sec	0.411363	0.008362	0.362698	0.273627
APOL1	Lag_time_sec	-0.40342	0.009844	0.362698	0.273627
C3	eGDRWC	-0.40619	0.009304	0.362698	0.273627
C4BPA	Max_absorbance	0.417613	0.007336	0.362698	0.273627
C4BPB	Lysis50_time_sec	0.40035	0.010473	0.362698	0.273627
C5	HbA1c	0.431845	0.005394	0.362698	0.273627
C5	Lysis50_time_sec	0.451343	0.003464	0.362698	0.273627
C9	Max_absorbance	0.446942	0.003837	0.362698	0.273627
CFH	eGDRWC	-0.42803	0.005865	0.362698	0.273627
CFH	Lag_time_sec	-0.41101	0.008423	0.362698	0.273627
CFH	Max_absorbance	0.433921	0.005152	0.362698	0.273627
CFHR5	Lag_time_sec	-0.43736	0.004771	0.362698	0.273627

CTSD	Lag_time_sec	0.410825	0.008456	0.362698	0.273627
FGA	Max_absorbance	0.408848	0.008809	0.362698	0.273627
FGB	Max_absorbance	0.412439	0.008177	0.362698	0.273627
IGHG4	HbA1c	-0.40386	0.009756	0.362698	0.273627
IGHV2-70D	eGDRWC	-0.442	0.004297	0.362698	0.273627
IGHV6-1	eGDRWC	0.403558	0.009816	0.362698	0.273627
IGKV1-12	Lysis50_time_sec	-0.44676	0.003853	0.362698	0.273627
IGKV1-17	HbA1c	-0.43956	0.004541	0.362698	0.273627
IGKV1-5	HbA1c	-0.4091	0.008764	0.362698	0.273627
IGKV2D-24	eGDRWC	0.41338	0.008018	0.362698	0.273627
IGKV4-1	HbA1c	-0.40126	0.010282	0.362698	0.273627
IGLV1-40	eGDRWC	0.409063	0.00877	0.362698	0.273627
IGLV7-46	Lag_time_sec	0.426986	0.006	0.362698	0.273627
IGLV8-61	eGDRWC	0.439303	0.004567	0.362698	0.273627
ITIH2	Max_absorbance	-0.41705	0.007424	0.362698	0.273627
LRG1	HbA1c	0.399793	0.010591	0.362698	0.273627
LRG1	Max_absorbance	0.420297	0.006929	0.362698	0.273627
MAN1A1	Lysis50_time_sec	0.423252	0.006504	0.362698	0.273627
MASP1	eGDRWC	-0.40257	0.010014	0.362698	0.273627
MST1	eGDRWC	0.440161	0.00448	0.362698	0.273627
NRP1	Lysis50_time_sec	-0.4082	0.008928	0.362698	0.273627
PIP	Clot_lysis_area	-0.40457	0.009616	0.362698	0.273627
PRG4	Lag_time_sec	-0.4172	0.0074	0.362698	0.273627
PRG4	Max_absorbance	0.456303	0.003083	0.362698	0.273627
PROS1	HbA1c	0.426261	0.006095	0.362698	0.273627
PRR4	Max_absorbance	0.415574	0.007658	0.362698	0.273627
SERPIND1	Max_absorbance	0.404153	0.009698	0.362698	0.273627
SHBG	Lysis50_time_sec	-0.41331	0.00803	0.362698	0.273627
SOD3	Lag_time_sec	0.436048	0.004914	0.362698	0.273627
TF	Max_absorbance	-0.43634	0.004882	0.362698	0.273627
C4BPA	Lysis50_time_sec	0.395508	0.011536	0.364956	0.27533
C5	eGDRWC	-0.3953	0.011585	0.364956	0.27533

 © Thieme. All rights reserved.

FGA	Clot_lysis_area	0.394801	0.011698	0.364956	0.27533
PPBP	Lag_time_sec	-0.3978	0.011021	0.364956	0.27533
RBP4	HbA1c	-0.39434	0.011804	0.364956	0.27533
VWF	eGDRWC	-0.39697	0.011207	0.364956	0.27533
IGHV3-38-3	Lag_time_sec	0.39101	0.012604	0.374095	0.282224
IGLV7-43	eGDRWC	0.391648	0.012448	0.374095	0.282224
C4BPA	Clot_lysis_area	0.386759	0.01369	0.374289	0.282372
FGA	eGDRWC	-0.38576	0.013956	0.374289	0.282372
IGHV3-15	HbA1c	-0.38903	0.0131	0.374289	0.282372
IGHV4-28	HbA1c	-0.38827	0.013294	0.374289	0.282372
IGKV1-27	eGDRWC	0.39017	0.012813	0.374289	0.282372
PRSS3	Max_absorbance	-0.38611	0.013864	0.374289	0.282372
CETP	eGDRWC	0.383807	0.01449	0.375053	0.282947
F5	Clot_lysis_area	0.384228	0.014373	0.375053	0.282947
IGKV1-27	HbA1c	-0.38478	0.014222	0.375053	0.282947
CFH	Lysis50_time_sec	0.380857	0.015328	0.383266	0.289144
IGHG4	Lysis50_time_sec	-0.38148	0.015148	0.383266	0.289144
IGKV1-6	Clot_lysis_area	-0.3811	0.015257	0.383266	0.289144
IGKV1-37	eGDRWC	0.378267	0.016098	0.393774	0.297071
IGKV4-1	eGDRWC	0.376262	0.016716	0.404443	0.30512
C1RL	Lysis50_time_sec	0.372408	0.017959	0.420818	0.317473
SERPINF1	Max_absorbance	-0.37069	0.018538	0.429849	0.324287
AFM	Lysis50_time_sec	0.360798	0.022186	0.446049	0.336509
C4BPA	eGDRWC	-0.36753	0.019645	0.446049	0.336509
CPB2	HbA1c	0.366322	0.020082	0.446049	0.336509
CST3	Max_absorbance	-0.36124	0.022011	0.446049	0.336509
FGG	Clot_lysis_area	0.361557	0.021886	0.446049	0.336509
GPLD1	HbA1c	-0.36746	0.01967	0.446049	0.336509
IGKC	HbA1c	-0.36483	0.020633	0.446049	0.336509
ITIH4	HbA1c	0.362371	0.021569	0.446049	0.336509
LBP	Clot_lysis_area	0.360154	0.022443	0.446049	0.336509
NID1	eGDRWC	-0.3634	0.021173	0.446049	0.336509

PLTP	eGDRWC	0.365824	0.020264	0.446049	0.336509
PLXDC2	eGDRWC	-0.36191	0.021749	0.446049	0.336509
PRDX2	Lag_time_sec	0.362959	0.021342	0.446049	0.336509
BCHE	eGDRWC	-0.35501	0.024585	0.456053	0.344055
C6	eGDRWC	-0.35821	0.023234	0.456053	0.344055
CFB	Max_absorbance	0.355357	0.024435	0.456053	0.344055
GC	HbA1c	0.355163	0.024519	0.456053	0.344055
IGHV2-26	Lag_time_sec	0.356948	0.023759	0.456053	0.344055
IGLV1-51	eGDRWC	0.357606	0.023484	0.456053	0.344055
LBP	eGDRWC	-0.35669	0.023866	0.456053	0.344055
TF	Clot_lysis_area	-0.35595	0.024179	0.456053	0.344055
CRISP3	eGDRWC	-0.35452	0.024796	0.456164	0.34414
CD5L	HbA1c	-0.35397	0.025038	0.456847	0.344655
BTD	eGDRWC	-0.3533	0.025332	0.458455	0.345868
A2M	Max_absorbance	-0.3508	0.026463	0.462986	0.349286
HSPA5	HbA1c	0.350452	0.026623	0.462986	0.349286
RARRES2	Lag_time_sec	0.351487	0.026148	0.462986	0.349286
CFB	Lysis50_time_sec	0.349417	0.027104	0.464108	0.350133
GC	eGDRWC	-0.34952	0.027055	0.464108	0.350133
C8A	eGDRWC	-0.34884	0.027375	0.465171	0.350934
IGKV3D-7	Clot_lysis_area	-0.34809	0.027731	0.467638	0.352795
F9	Lag_time_sec	-0.34659	0.028455	0.473424	0.357161
C1S	eGDRWC	-0.34479	0.029347	0.473434	0.357168
IGKV1-12	Clot_lysis_area	-0.34436	0.029563	0.473434	0.357168
IGKV1-17	eGDRWC	0.344478	0.029503	0.473434	0.357168
JCHAIN	Max_absorbance	-0.34436	0.02956	0.473434	0.357168
PRG4	Lysis50_time_sec	0.34562	0.028934	0.473434	0.357168
APOA1	Max_absorbance	-0.33911	0.032306	0.476936	0.35981
APOB	Max_absorbance	0.327295	0.039254	0.476936	0.35981
C1R	Clot_lysis_area	0.339491	0.032101	0.476936	0.35981
C1RL	Clot_lysis_area	0.328572	0.038449	0.476936	0.35981
C2	HbA1c	0.323799	0.041528	0.476936	0.35981

C2	Max_absorbance	0.324245	0.041231	0.476936	0.35981
C4BPB	HbA1c	0.335944	0.034061	0.476936	0.35981
C4B_2	eGDRWC	-0.32643	0.039808	0.476936	0.35981
CA1	Clot_lysis_area	0.331145	0.036868	0.476936	0.35981
CD14	Lag_time_sec	-0.32757	0.039078	0.476936	0.35981
CFB	HbA1c	0.339665	0.032007	0.476936	0.35981
CFD	HbA1c	-0.33434	0.03498	0.476936	0.35981
CFD	Max_absorbance	0.338279	0.03276	0.476936	0.35981
CFHR4	HbA1c	0.339982	0.031837	0.476936	0.35981
CNDP1	Lag_time_sec	-0.33451	0.034878	0.476936	0.35981
CP	Max_absorbance	0.338289	0.032755	0.476936	0.35981
F11	Lag_time_sec	-0.32588	0.040164	0.476936	0.35981
FCGR3B	Lysis50_time_sec	0.333418	0.035515	0.476936	0.35981
HABP2	Clot_lysis_area	0.32678	0.039582	0.476936	0.35981
HSPA6	Max_absorbance	0.327515	0.039114	0.476936	0.35981
IGHV1-18	Clot_lysis_area	-0.3349	0.034655	0.476936	0.35981
IGHV1-24	Lysis50_time_sec	-0.34154	0.03101	0.476936	0.35981
IGHV1-45	Clot_lysis_area	-0.32642	0.039814	0.476936	0.35981
IGHV1-46	Clot_lysis_area	-0.33896	0.032389	0.476936	0.35981
IGHV3-15	eGDRWC	0.337161	0.033378	0.476936	0.35981
IGHV6-1	HbA1c	-0.32485	0.040833	0.476936	0.35981
IGKC	eGDRWC	0.324813	0.040857	0.476936	0.35981
IGKV3-11	Max_absorbance	-0.33188	0.036426	0.476936	0.35981
IGKV3D-7	Max_absorbance	-0.33105	0.036925	0.476936	0.35981
IGLC3	eGDRWC	0.338788	0.032482	0.476936	0.35981
IGLV2-14	Lysis50_time_sec	-0.3341	0.035117	0.476936	0.35981
IGLV3-21	Lysis50_time_sec	-0.33964	0.032022	0.476936	0.35981
IGLV5-39	HbA1c	-0.34018	0.031732	0.476936	0.35981
IGLV7-46	eGDRWC	0.324356	0.041158	0.476936	0.35981
IGLV8-61	Lysis50_time_sec	-0.32552	0.040395	0.476936	0.35981
ITIH4	Max_absorbance	0.324463	0.041087	0.476936	0.35981
LILRA3	Max_absorbance	-0.32671	0.039624	0.476936	0.35981

LUM	Lag_time_sec	0.323143	0.041966	0.476936	0.35981
PI16	eGDRWC	0.335976	0.034043	0.476936	0.35981
PIP	Max_absorbance	-0.33074	0.037112	0.476936	0.35981
PLTP	HbA1c	-0.3295	0.037872	0.476936	0.35981
PLXDC2	HbA1c	0.343139	0.030183	0.476936	0.35981
PPBP	HbA1c	0.324653	0.040963	0.476936	0.35981
PROCR	Lag_time_sec	0.334534	0.034867	0.476936	0.35981
PROS1	Lysis50_time_sec	0.335618	0.034246	0.476936	0.35981
SELL	Clot_lysis_area	-0.32464	0.040971	0.476936	0.35981
SERPINA10	HbA1c	0.323101	0.041994	0.476936	0.35981
SHBG	eGDRWC	0.323122	0.04198	0.476936	0.35981
VTN	Lag_time_sec	-0.32965	0.037782	0.476936	0.35981
GSN	Lysis50_time_sec	-0.32227	0.042553	0.478403	0.360917
SOD3	HbA1c	-0.32248	0.04241	0.478403	0.360917
ART3	Lysis50_time_sec	-0.31917	0.044702	0.486281	0.36686
CPB2	eGDRWC	-0.31944	0.044509	0.486281	0.36686
GAPDH	Lysis50_time_sec	0.319089	0.04476	0.486281	0.36686
IGHV1-45	Lysis50_time_sec	-0.31875	0.045002	0.486281	0.36686
IGHV2-26	Clot_lysis_area	-0.32042	0.043826	0.486281	0.36686
IGLV7-46	Lysis50_time_sec	-0.32007	0.044072	0.486281	0.36686
KLKB1	HbA1c	0.32019	0.043986	0.486281	0.36686
C3	HbA1c	0.318197	0.045394	0.488149	0.36827
IGFALS	Lag_time_sec	-0.31694	0.046298	0.490761	0.37024
IGKV2-40	Max_absorbance	0.317472	0.045915	0.490761	0.37024
F11	Lysis50_time_sec	0.316547	0.046587	0.491478	0.370781
F2	HbA1c	0.315668	0.047233	0.493308	0.372161
IGLV5-45	eGDRWC	0.315558	0.047314	0.493308	0.372161
C5	Max_absorbance	0.313371	0.048954	0.497104	0.375026
CNDP1	eGDRWC	-0.31322	0.049068	0.497104	0.375026
IGHV1-46	HbA1c	-0.31409	0.048411	0.497104	0.375026
IGLV7-43	HbA1c	-0.31408	0.048415	0.497104	0.375026
SERPINA3	Lysis50_time_sec	0.313355	0.048966	0.497104	0.375026

F10	eGDRWC	-0.3125	0.049617	0.497506	0.375329
HSPG2	Max_absorbance	-0.31261	0.049534	0.497506	0.375329
IGKV3-7	Lysis50_time_sec	-0.31212	0.049913	0.498237	0.37588
IGKV2D-24	HbA1c	-0.31163	0.05029	0.499758	0.377028
APOC4	Lag_time_sec	-0.30962	0.051877	0.502076	0.378776
BTD	HbA1c	0.309816	0.051717	0.502076	0.378776
IGHV1-46	eGDRWC	0.310556	0.051132	0.502076	0.378776
MBL2	Lag_time_sec	-0.31017	0.051433	0.502076	0.378776
TFRC	Lysis50_time_sec	0.309829	0.051707	0.502076	0.378776
AGT	Lag_time_sec	-0.30081	0.059279	0.50495	0.380945
AHSG	Lag_time_sec	-0.30598	0.054836	0.50495	0.380945
APOB	Clot_lysis_area	0.300145	0.059872	0.50495	0.380945
APOF	Lysis50_time_sec	-0.3064	0.054489	0.50495	0.380945
C3	Lysis50_time_sec	0.299808	0.060174	0.50495	0.380945
C4A	Clot_lysis_area	-0.3061	0.054739	0.50495	0.380945
C4BPB	eGDRWC	-0.29846	0.06139	0.50495	0.380945
CFH	HbA1c	0.303059	0.057314	0.50495	0.380945
CFI	HbA1c	0.300192	0.05983	0.50495	0.380945
F13A1	Lag_time_sec	-0.29888	0.061014	0.50495	0.380945
FCGR3A	Lag_time_sec	-0.30728	0.053767	0.50495	0.380945
FGG	eGDRWC	-0.29967	0.060301	0.50495	0.380945
HABP2	Max_absorbance	0.299788	0.060192	0.50495	0.380945
HBA2	Clot_lysis_area	0.304322	0.056233	0.50495	0.380945
HBD	Lysis50_time_sec	0.297828	0.06197	0.50495	0.380945
IGFALS	eGDRWC	-0.30548	0.055257	0.50495	0.380945
IGHV1-18	Lag_time_sec	0.29797	0.06184	0.50495	0.380945
IGHV1-2	Lag_time_sec	0.304684	0.055926	0.50495	0.380945
IGHV3-35	Lag_time_sec	0.297875	0.061928	0.50495	0.380945
IGHV3-64D	eGDRWC	0.302395	0.057889	0.50495	0.380945
IGHV3-72	Clot_lysis_area	0.306309	0.054564	0.50495	0.380945
IGHV3-9	Lag_time_sec	0.297363	0.062399	0.50495	0.380945
IGHV4-28	eGDRWC	0.305855	0.054942	0.50495	0.380945

© Thieme. All rights reserved.

IGHV6-1	Lysis50_time_sec	-0.30324	0.057157	0.50495	0.380945
IGKV1-37	HbA1c	-0.29968	0.060286	0.50495	0.380945
IGKV1D-16	Clot_lysis_area	-0.29781	0.061989	0.50495	0.380945
IGKV3D-15	eGDRWC	0.300956	0.059152	0.50495	0.380945
IGLC3	HbA1c	-0.30733	0.05372	0.50495	0.380945
IGLC7	eGDRWC	0.304315	0.056239	0.50495	0.380945
IGLV1-51	HbA1c	-0.29893	0.060965	0.50495	0.380945
IGLV8-61	HbA1c	-0.30243	0.057856	0.50495	0.380945
MAN1A1	HbA1c	0.303964	0.056537	0.50495	0.380945
NRP1	HbA1c	-0.29807	0.061745	0.50495	0.380945
ORM1	Lag_time_sec	-0.29991	0.060084	0.50495	0.380945
PI16	HbA1c	-0.29773	0.062061	0.50495	0.380945
PPBP	Lysis50_time_sec	0.300664	0.05941	0.50495	0.380945
PROZ	eGDRWC	0.299068	0.060841	0.50495	0.380945
SAA2	HbA1c	0.299823	0.06016	0.50495	0.380945
SERPINA5	eGDRWC	-0.29734	0.062417	0.50495	0.380945
SERPIND1	Lag_time_sec	-0.29714	0.062608	0.50495	0.380945
VASN	HbA1c	0.305746	0.055033	0.50495	0.380945
VASN	eGDRWC	-0.30058	0.059485	0.50495	0.380945
CDH13	Max_absorbance	-0.29611	0.063563	0.508963	0.383972
TFRC	HbA1c	0.294934	0.064673	0.515989	0.389273
IGHV2-5	eGDRWC	-0.29386	0.065696	0.522282	0.39402
ALCAM	HbA1c	0.293342	0.066199	0.522549	0.394221
PRG4	Clot_lysis_area	0.293569	0.065979	0.522549	0.394221
HGFAC	eGDRWC	0.292089	0.06742	0.526587	0.397268
IGLV5-39	Lag_time_sec	0.292161	0.067349	0.526587	0.397268
PTGDS	Clot_lysis_area	-0.29129	0.068209	0.530889	0.400513
PON3	Max_absorbance	-0.29082	0.068677	0.532667	0.401855
IGHV3-38-3	Lysis50_time_sec	-0.28994	0.069559	0.534625	0.403332
LGALS3BP	eGDRWC	-0.28961	0.06989	0.534625	0.403332
ORM2	Lysis50_time_sec	0.288553	0.070961	0.536289	0.404587
C6	Clot_lysis_area	0.287888	0.071643	0.536959	0.405093

IGKV1-13	HbA1c	-0.28803	0.071502	0.536959	0.405093
IGHV1-46	Max_absorbance	-0.28725	0.072305	0.537137	0.405227
IGLV3-9	Clot_lysis_area	-0.28693	0.072632	0.537137	0.405227
PGLYRP2	Max_absorbance	-0.28715	0.072411	0.537137	0.405227
SERPINA6	HbA1c	0.287525	0.072017	0.537137	0.405227
PTGDS	Lysis50_time_sec	-0.286	0.073605	0.542321	0.409138
MCAM	eGDRWC	0.285186	0.074468	0.543491	0.41002
ALDOA	eGDRWC	0.28321	0.076588	0.544047	0.41044
APCS	Max_absorbance	0.284612	0.075079	0.544047	0.41044
APOL1	HbA1c	0.280208	0.0799	0.544047	0.41044
B4GALT1	eGDRWC	-0.27893	0.081343	0.544047	0.41044
C4BPB	Clot_lysis_area	0.278752	0.081546	0.544047	0.41044
CETP	HbA1c	-0.28187	0.078056	0.544047	0.41044
CFHR5	Max_absorbance	0.282237	0.077649	0.544047	0.41044
CPB2	Lysis50_time_sec	0.280201	0.079907	0.544047	0.41044
CPN2	Lysis50_time_sec	0.279454	0.080749	0.544047	0.41044
CRISP3	HbA1c	0.277621	0.082842	0.544047	0.41044
FBLN5	Lysis50_time_sec	0.28336	0.076426	0.544047	0.41044
FCGBP	Lag_time_sec	-0.28291	0.076917	0.544047	0.41044
FN1	Lag_time_sec	-0.27787	0.082559	0.544047	0.41044
IGHV3-38-3	HbA1c	-0.27985	0.080306	0.544047	0.41044
IGHV3-7	eGDRWC	0.281789	0.078142	0.544047	0.41044
IGHV3OR16-9	Lysis50_time_sec	-0.27981	0.08035	0.544047	0.41044
IGKV1-33	Max_absorbance	-0.27881	0.081476	0.544047	0.41044
IGKV1-33	Lysis50_time_sec	-0.2788	0.081486	0.544047	0.41044
ITIH1	Lag_time_sec	-0.27906	0.081196	0.544047	0.41044
MCAM	Clot_lysis_area	0.282162	0.077731	0.544047	0.41044
PI16	Lysis50_time_sec	-0.2784	0.081944	0.544047	0.41044
PON3	Clot_lysis_area	-0.27761	0.082853	0.544047	0.41044
PRG4	eGDRWC	-0.27816	0.08222	0.544047	0.41044
SAA2	Clot_lysis_area	0.281864	0.078059	0.544047	0.41044
SERPINA10	Lysis50_time_sec	0.277634	0.082827	0.544047	0.41044

SERPINA4	Max_absorbance	-0.28213	0.077765	0.544047	0.41044
SERPINF2	Max_absorbance	0.278456	0.081883	0.544047	0.41044
SERPING1	Lysis50_time_sec	0.279646	0.080532	0.544047	0.41044
VTN	Clot_lysis_area	0.279628	0.080552	0.544047	0.41044
IGLC7	HbA1c	-0.27733	0.083175	0.544551	0.41082
COL6A3	Lag_time_sec	0.276882	0.083698	0.546371	0.412193
IGHV2-70D	HbA1c	0.276662	0.083954	0.54644	0.412245
C1QC	Max_absorbance	-0.2748	0.086146	0.546575	0.412347
C4BPB	Max_absorbance	0.275976	0.084756	0.546575	0.412347
C9	Lysis50_time_sec	0.276313	0.084361	0.546575	0.412347
CDH13	Lysis50_time_sec	-0.27466	0.086313	0.546575	0.412347
IGHV1-69	Lag_time_sec	-0.27616	0.084546	0.546575	0.412347
IGHV1-69	Clot_lysis_area	-0.27483	0.086106	0.546575	0.412347
ORM1	Max_absorbance	0.274561	0.086431	0.546575	0.412347
CP	Clot_lysis_area	0.274032	0.087062	0.54746	0.413015
C6	Max_absorbance	0.273545	0.087648	0.549592	0.414624
SELENOP	HbA1c	0.273099	0.088187	0.551413	0.415997
APOB	HbA1c	0.272423	0.089008	0.554991	0.418697
APOC4	eGDRWC	-0.27172	0.089864	0.557782	0.420802
HBD	eGDRWC	-0.27165	0.089957	0.557782	0.420802
F13B	Max_absorbance	0.271236	0.090464	0.559319	0.421962
PRDX2	Clot_lysis_area	0.27104	0.090707	0.559319	0.421962
ANTXR1	Lag_time_sec	0.26857	0.093804	0.574582	0.433477
APCS	Lag_time_sec	-0.26842	0.093995	0.574582	0.433477
APOD	Lysis50_time_sec	-0.26729	0.095435	0.574582	0.433477
C2	Lysis50_time_sec	0.266934	0.095899	0.574582	0.433477
CFHR4	eGDRWC	-0.26684	0.096022	0.574582	0.433477
CPN1	Clot_lysis_area	0.266973	0.095849	0.574582	0.433477
HSPA6	Lysis50_time_sec	0.267366	0.095342	0.574582	0.433477
ORM2	Lag_time_sec	-0.26705	0.095746	0.574582	0.433477
PCYOX1	Lag_time_sec	-0.26836	0.094075	0.574582	0.433477
PRSS3	Clot_lysis_area	-0.26805	0.094469	0.574582	0.433477

© Thieme. All rights reserved.

VWF	Lysis50_time_sec	0.267202	0.095553	0.574582	0.433477
SERPINF2	Lysis50_time_sec	0.26652	0.096434	0.5755	0.434169
PRDX2	eGDRWC	-0.26566	0.097558	0.577272	0.435505
SHBG	Max_absorbance	-0.26554	0.097712	0.577272	0.435505
AFM	eGDRWC	-0.26443	0.09917	0.580563	0.437989
IGKV6-21	Lysis50_time_sec	-0.26404	0.099697	0.580563	0.437989
IGLV7-46	HbA1c	-0.26437	0.099252	0.580563	0.437989
RARRES2	Lysis50_time_sec	-0.26414	0.099561	0.580563	0.437989
APOD	Max_absorbance	-0.26331	0.100672	0.581373	0.438599
ITIH4	Clot_lysis_area	0.263542	0.100356	0.581373	0.438599
ANGPTL6	HbA1c	-0.26241	0.101873	0.582957	0.439794
IGKV6-21	Clot_lysis_area	-0.26257	0.101665	0.582957	0.439794
PIGR	Lysis50_time_sec	0.261958	0.102493	0.584998	0.441335
AHSG	Max_absorbance	-0.26161	0.10296	0.585211	0.441495
C9	HbA1c	0.260805	0.104069	0.585211	0.441495
CD14	Max_absorbance	0.260911	0.103923	0.585211	0.441495
IGKV1-8	eGDRWC	0.261126	0.103629	0.585211	0.441495
MASP2	eGDRWC	-0.26078	0.104108	0.585211	0.441495
MMP2	Lag_time_sec	0.261246	0.103463	0.585211	0.441495
FCGR3A	Lysis50_time_sec	0.260144	0.104981	0.586205	0.442245
IGKV1-33	Clot_lysis_area	-0.26029	0.104778	0.586205	0.442245
A2M	Lysis50_time_sec	-0.25914	0.106375	0.588387	0.443891
IGHV3-38	Clot_lysis_area	0.258812	0.106836	0.588387	0.443891
IGKV3-11	Clot_lysis_area	-0.25866	0.107049	0.588387	0.443891
MASP2	HbA1c	0.258471	0.107316	0.588387	0.443891
PROC	Lag_time_sec	0.258482	0.1073	0.588387	0.443891
C8B	HbA1c	0.258007	0.10797	0.59052	0.4455
IGLV5-39	eGDRWC	0.257092	0.10927	0.594707	0.448659
SERPIND1	Lysis50_time_sec	0.257215	0.109095	0.594707	0.448659
CETP	Max_absorbance	-0.25644	0.110205	0.596175	0.449767
IGHA1	Max_absorbance	-0.25655	0.110047	0.596175	0.449767
TNXB	Lysis50_time_sec	-0.25634	0.110343	0.596175	0.449767

BCHE	Clot_lysis_area	0.256137	0.11064	0.59633	0.449883
SERPINA1	Lysis50_time_sec	0.255801	0.111124	0.597494	0.450762
A1BG	Lag_time_sec	0.254673	0.112763	0.597551	0.450804
A2M	Clot_lysis_area	-0.25448	0.113045	0.597551	0.450804
ANGPTL6	eGDRWC	0.253668	0.114239	0.597551	0.450804
C4A	eGDRWC	-0.253	0.115227	0.597551	0.450804
C8G	Clot_lysis_area	0.255316	0.111826	0.597551	0.450804
CASP14	Max_absorbance	-0.25436	0.113227	0.597551	0.450804
CD5L	eGDRWC	0.25313	0.115035	0.597551	0.450804
CNDP1	HbA1c	0.254493	0.113027	0.597551	0.450804
ECM1	eGDRWC	-0.25422	0.113429	0.597551	0.450804
FGA	HbA1c	0.253187	0.114951	0.597551	0.450804
HABP2	Lag_time_sec	-0.25411	0.113584	0.597551	0.450804
IGHV1-3	HbA1c	0.25373	0.114148	0.597551	0.450804
IGKV6-21	eGDRWC	0.254287	0.113329	0.597551	0.450804
PROS1	Max_absorbance	0.252865	0.11543	0.597551	0.450804
SERPINA3	Max_absorbance	0.253108	0.115068	0.597551	0.450804
HBB	Clot_lysis_area	0.252619	0.115795	0.59805	0.451181
ABCB9	eGDRWC	-0.25207	0.116621	0.599535	0.452301
IGHV3-43	Max_absorbance	-0.25219	0.116437	0.599535	0.452301
GSN	HbA1c	-0.25143	0.117575	0.59958	0.452335
IGLV1-40	HbA1c	-0.2514	0.117619	0.59958	0.452335
LBP	HbA1c	0.250988	0.118246	0.59958	0.452335
PROCR	Max_absorbance	-0.251	0.118227	0.59958	0.452335
LDHA	Lysis50_time_sec	0.249903	0.119899	0.606579	0.457615
CD44	Lag_time_sec	0.249626	0.120324	0.607349	0.458196
CLEC3B	Lysis50_time_sec	0.249405	0.120663	0.607609	0.458392
C4B_2	HbA1c	0.247189	0.124106	0.607731	0.458485
COLEC11	Max_absorbance	-0.24735	0.123854	0.607731	0.458485
F10	HbA1c	0.247828	0.123106	0.607731	0.458485
HP	Lysis50_time_sec	0.247492	0.123632	0.607731	0.458485
HRG	Lysis50_time_sec	-0.24712	0.124222	0.607731	0.458485

IGHV1-3	Lysis50_time_sec	-0.24837	0.122266	0.607731	0.458485
IGKV1-16	Lysis50_time_sec	-0.2485	0.122067	0.607731	0.458485
IGLV9-49	eGDRWC	-0.24817	0.122576	0.607731	0.458485
KLKB1	eGDRWC	-0.24774	0.123243	0.607731	0.458485
LPA	Clot_lysis_area	-0.24712	0.12422	0.607731	0.458485
LRG1	Clot_lysis_area	0.247169	0.124138	0.607731	0.458485
CFH	Clot_lysis_area	0.246423	0.125314	0.610391	0.460491
CFD	Clot_lysis_area	0.245619	0.126592	0.611265	0.46115
HRG	Lag_time_sec	-0.24589	0.12616	0.611265	0.46115
IGKV2-40	eGDRWC	-0.24576	0.126365	0.611265	0.46115
LILRA3	eGDRWC	-0.24604	0.125927	0.611265	0.46115
HPX	eGDRWC	0.244614	0.128202	0.616366	0.464999
IGKV6-21	Max_absorbance	-0.24417	0.128925	0.618505	0.466613
SOD3	eGDRWC	0.243584	0.129867	0.620352	0.468006
IGHV3-15	Lag_time_sec	0.243299	0.13033	0.621231	0.46867
PTPRJ	Lag_time_sec	-0.24249	0.131657	0.626216	0.47243
SERPING1	eGDRWC	-0.24205	0.132381	0.626979	0.473006
LTF	eGDRWC	0.241681	0.132987	0.628298	0.474001
PGLYRP2	Lysis50_time_sec	-0.24136	0.133525	0.628298	0.474001
PIP	eGDRWC	-0.2412	0.133789	0.628298	0.474001
DBH	Clot_lysis_area	0.240508	0.134938	0.62971	0.475066
SAA2	Max_absorbance	0.240668	0.134671	0.62971	0.475066
APOL1	eGDRWC	-0.24005	0.135696	0.630604	0.475741
IGFALS	HbA1c	0.239022	0.137438	0.636046	0.479846
RBP4	eGDRWC	0.239123	0.137268	0.636046	0.479846
THBS4	Clot_lysis_area	0.23877	0.137865	0.636698	0.480338
IGKV1D-16	eGDRWC	0.238569	0.138207	0.636954	0.480531
IGHV3-64	Clot_lysis_area	-0.23817	0.138888	0.638768	0.4819
IGHV1-24	Clot_lysis_area	-0.23799	0.139203	0.638901	0.482
F11	Clot_lysis_area	0.237485	0.140061	0.641516	0.483973
CDH13	Lag_time_sec	-0.23699	0.140908	0.643013	0.485102
CFI	Max_absorbance	0.236009	0.142615	0.643013	0.485102

IGF2R	HbA1c	-0.23678	0.141282	0.643013	0.485102
IGKV1-6	Lysis50_time_sec	-0.23611	0.142447	0.643013	0.485102
IGLV1-51	Lysis50_time_sec	-0.23661	0.141575	0.643013	0.485102
SOD3	Lysis50_time_sec	-0.23633	0.142055	0.643013	0.485102
APOA4	Max_absorbance	-0.23395	0.146229	0.645132	0.4867
APOB	Lag_time_sec	-0.2312	0.151176	0.645132	0.4867
C2	Lag_time_sec	-0.23367	0.14673	0.645132	0.4867
C4B_2	Lysis50_time_sec	0.235039	0.144312	0.645132	0.4867
CFB	Lag_time_sec	-0.23249	0.14884	0.645132	0.4867
CLU	Clot_lysis_area	0.231261	0.151062	0.645132	0.4867
COMP	Max_absorbance	0.235267	0.143911	0.645132	0.4867
EFEMP1	Clot_lysis_area	0.231231	0.151116	0.645132	0.4867
F11	HbA1c	0.232129	0.149491	0.645132	0.4867
F9	HbA1c	0.233979	0.146184	0.645132	0.4867
F9	eGDRWC	-0.23128	0.151022	0.645132	0.4867
FGG	Lag_time_sec	-0.23329	0.147406	0.645132	0.4867
FGG	Lysis50_time_sec	0.233488	0.147055	0.645132	0.4867
IGHM	HbA1c	-0.2334	0.147206	0.645132	0.4867
IGHV5-51	Clot_lysis_area	-0.2323	0.149183	0.645132	0.4867
IGKV1-6	Max_absorbance	-0.23111	0.151329	0.645132	0.4867
IGKV2-24	eGDRWC	0.230828	0.15185	0.645132	0.4867
IGKV3-11	Lag_time_sec	0.233059	0.147822	0.645132	0.4867
IGLC7	Lag_time_sec	0.231694	0.150277	0.645132	0.4867
IGLV1-47	Clot_lysis_area	-0.23304	0.147849	0.645132	0.4867
ITIH3	Max_absorbance	0.231311	0.150971	0.645132	0.4867
MASP2	Clot_lysis_area	-0.2329	0.148101	0.645132	0.4867
PI16	Lag_time_sec	0.232019	0.14969	0.645132	0.4867
SERPINA1	HbA1c	0.231702	0.150262	0.645132	0.4867
TGFBI	Lysis50_time_sec	0.234976	0.144422	0.645132	0.4867
TNXB	Clot_lysis_area	-0.23325	0.147477	0.645132	0.4867
ALCAM	Lag_time_sec	0.230476	0.152493	0.64534	0.486858
C9	Clot_lysis_area	0.230632	0.152209	0.64534	0.486858

HSPA5	eGDRWC	-0.23028	0.152861	0.645671	0.487108
APCS	HbA1c	0.229529	0.154233	0.64754	0.488517
DBH	Lag_time_sec	-0.22862	0.155922	0.64754	0.488517
GP1BA	Lag_time_sec	-0.22867	0.155832	0.64754	0.488517
IGFALS	Clot_lysis_area	0.229485	0.154313	0.64754	0.488517
IGHV3-7	HbA1c	-0.2289	0.155393	0.64754	0.488517
IGLV2-11	eGDRWC	0.229307	0.154642	0.64754	0.488517
APOE	Lag_time_sec	-0.2273	0.158384	0.649343	0.489878
GP1BA	Lysis50_time_sec	-0.22698	0.158981	0.649343	0.489878
GPX3	HbA1c	-0.22707	0.158816	0.649343	0.489878
IGHG3	Lysis50_time_sec	0.22794	0.157183	0.649343	0.489878
IGKV1-5	eGDRWC	0.227094	0.158772	0.649343	0.489878
IGKV6-21	HbA1c	-0.22765	0.15772	0.649343	0.489878
TNXB	Max_absorbance	-0.22713	0.1587	0.649343	0.489878
IGHV3-74	HbA1c	-0.2263	0.16027	0.651024	0.491146
PROZ	HbA1c	-0.22637	0.160141	0.651024	0.491146
SERPINA1	eGDRWC	-0.22655	0.159798	0.651024	0.491146
HABP2	eGDRWC	-0.2261	0.160645	0.651359	0.491399
AGT	HbA1c	0.22343	0.165785	0.652747	0.492445
BTD	Lag_time_sec	-0.2212	0.170165	0.652747	0.492445
C1R	eGDRWC	-0.22268	0.167239	0.652747	0.492445
C1RL	eGDRWC	-0.22114	0.170281	0.652747	0.492445
C1RL	Lag_time_sec	-0.22296	0.166699	0.652747	0.492445
C7	Clot_lysis_area	0.222496	0.167607	0.652747	0.492445
CA1	eGDRWC	-0.22238	0.167835	0.652747	0.492445
CDH5	Lag_time_sec	-0.22364	0.165378	0.652747	0.492445
CPN2	eGDRWC	-0.22156	0.16944	0.652747	0.492445
CPQ	Clot_lysis_area	0.220209	0.17213	0.652747	0.492445
DBH	Lysis50_time_sec	0.223102	0.166423	0.652747	0.492445
F13A1	Clot_lysis_area	0.221232	0.170096	0.652747	0.492445
F13B	Lag_time_sec	-0.22118	0.1702	0.652747	0.492445
HSPG2	Lysis50_time_sec	-0.22217	0.168256	0.652747	0.492445

IGHA1	Clot_lysis_area	-0.22305	0.166517	0.652747	0.492445
IGHV3-13	HbA1c	-0.22046	0.171631	0.652747	0.492445
IGHV3-13	Lag_time_sec	0.225179	0.162409	0.652747	0.492445
IGKV2-24	Lag_time_sec	-0.22275	0.167108	0.652747	0.492445
IGKV2D-30	Lysis50_time_sec	0.221719	0.169134	0.652747	0.492445
IGKV3-11	Lysis50_time_sec	-0.22024	0.17206	0.652747	0.492445
IGKV3D-7	HbA1c	0.221586	0.169397	0.652747	0.492445
IGLV1-36	Lysis50_time_sec	-0.22024	0.172072	0.652747	0.492445
IGLV3-21	Clot_lysis_area	-0.22058	0.171388	0.652747	0.492445
IGLV3-9	Max_absorbance	-0.22306	0.166495	0.652747	0.492445
LYVE1	Clot_lysis_area	-0.22276	0.167095	0.652747	0.492445
PCYOX1	Clot_lysis_area	0.22316	0.166309	0.652747	0.492445
PON1	Lag_time_sec	-0.22317	0.166284	0.652747	0.492445
PRG4	HbA1c	0.224308	0.164083	0.652747	0.492445
SERPINA10	eGDRWC	-0.22345	0.165746	0.652747	0.492445
SERPINF1	eGDRWC	-0.22247	0.167656	0.652747	0.492445
SERPINF2	Clot_lysis_area	0.223915	0.164843	0.652747	0.492445
TFRC	Lag_time_sec	-0.22052	0.171508	0.652747	0.492445
IGKV1-39	Lysis50_time_sec	-0.21995	0.172652	0.653611	0.493097
IGHV1OR15-1	Clot_lysis_area	-0.21952	0.173509	0.655741	0.494704
APOC1	eGDRWC	0.217958	0.176668	0.658151	0.496522
HP	Lag_time_sec	-0.21809	0.176409	0.658151	0.496522
IGLV3-10	Lag_time_sec	0.217889	0.176808	0.658151	0.496522
PTPRJ	Max_absorbance	-0.21871	0.175146	0.658151	0.496522
ADAMTS13	HbA1c	-0.21621	0.180261	0.660404	0.498222
ANTXR1	Max_absorbance	-0.21474	0.183301	0.660404	0.498222
APMAP	Lag_time_sec	0.214581	0.183636	0.660404	0.498222
APOL1	Lysis50_time_sec	0.213617	0.185659	0.660404	0.498222
ART3	Max_absorbance	-0.21489	0.182986	0.660404	0.498222
CA1	Lag_time_sec	0.216848	0.178937	0.660404	0.498222
CETP	Lag_time_sec	0.216296	0.180073	0.660404	0.498222
CFHR1	HbA1c	-0.21395	0.184962	0.660404	0.498222

CFI	Lysis50_time_sec	0.213895	0.185074	0.660404	0.498222
CST3	Clot_lysis_area	-0.21447	0.183872	0.660404	0.498222
F7	Lag_time_sec	-0.21666	0.179314	0.660404	0.498222
FCGR3A	Max_absorbance	0.215801	0.181097	0.660404	0.498222
HBB	Lysis50_time_sec	0.213754	0.18537	0.660404	0.498222
ICOSLG	Max_absorbance	-0.2162	0.180265	0.660404	0.498222
IGHA2	Lag_time_sec	0.215684	0.181338	0.660404	0.498222
IGHV1-2	Clot_lysis_area	-0.21531	0.182121	0.660404	0.498222
IGHV1-45	Max_absorbance	-0.21709	0.178432	0.660404	0.498222
IGHV2-5	Clot_lysis_area	0.215523	0.181674	0.660404	0.498222
IGHV3-13	eGDRWC	0.213588	0.18572	0.660404	0.498222
IGHV3-38-3	eGDRWC	0.214433	0.183945	0.660404	0.498222
IGHV3-7	Max_absorbance	-0.21362	0.185663	0.660404	0.498222
IGKV2-30	Max_absorbance	0.216196	0.18028	0.660404	0.498222
IGKV3D-15	HbA1c	-0.21656	0.179526	0.660404	0.498222
ITIH4	Lysis50_time_sec	0.215119	0.182513	0.660404	0.498222
PROCR	HbA1c	-0.21722	0.178178	0.660404	0.498222
SERPINA10	Lag_time_sec	-0.21459	0.183626	0.660404	0.498222
VWF	HbA1c	0.21367	0.185548	0.660404	0.498222
APCS	Lysis50_time_sec	0.212324	0.188398	0.660433	0.498244
ART3	Clot_lysis_area	-0.21245	0.188135	0.660433	0.498244
CA1	Lysis50_time_sec	0.213344	0.186235	0.660433	0.498244
FCGBP	Max_absorbance	-0.21291	0.187153	0.660433	0.498244
HPR	eGDRWC	-0.21326	0.18642	0.660433	0.498244
IGHV3-9	eGDRWC	0.212621	0.187768	0.660433	0.498244
IGKV2D-24	Max_absorbance	-0.21275	0.18749	0.660433	0.498244
B2M	Lag_time_sec	-0.21149	0.190189	0.664016	0.500947
HSPA5	Lysis50_time_sec	0.21144	0.190289	0.664016	0.500947
APOA2	eGDRWC	-0.21076	0.191755	0.665907	0.502374
CDH5	Max_absorbance	-0.21084	0.191569	0.665907	0.502374
CLU	eGDRWC	-0.21102	0.191189	0.665907	0.502374
CPN2	HbA1c	0.210307	0.192731	0.666403	0.502748

IGLV5-45	HbA1c	-0.21032	0.192708	0.666403	0.502748
SERPIND1	Clot_lysis_area	0.210277	0.192796	0.666403	0.502748
IGHV3-74	eGDRWC	0.210131	0.193111	0.666458	0.502789
A0A0J9YY99	Lag_time_sec	0.209414	0.194669	0.667695	0.503722
ORM1	HbA1c	0.209455	0.19458	0.667695	0.503722
LGALS3BP	HbA1c	0.208806	0.195999	0.670189	0.505604
SERPINA1	Lag_time_sec	-0.20892	0.19574	0.670189	0.505604
A0A0J9YY99	Max_absorbance	-0.20828	0.197154	0.673104	0.507803
APOF	Max_absorbance	-0.20762	0.198607	0.675362	0.509507
CP	Lag_time_sec	-0.20757	0.198725	0.675362	0.509507
PTGDS	HbA1c	-0.20775	0.198324	0.675362	0.509507
AFM	Lag_time_sec	-0.20715	0.199642	0.675385	0.509524
CPQ	Lysis50_time_sec	0.207267	0.19939	0.675385	0.509524
IGHV6-1	Lag_time_sec	0.207419	0.199053	0.675385	0.509524
NID1	HbA1c	0.206985	0.200016	0.675623	0.509704
IGHV3-49	HbA1c	-0.20681	0.200412	0.675936	0.509939
CFI	eGDRWC	-0.2057	0.202884	0.681065	0.513809
COMP	Clot_lysis_area	0.205796	0.202669	0.681065	0.513809
LRP1	Max_absorbance	-0.20558	0.203157	0.681065	0.513809
ATRN	HbA1c	0.204021	0.206677	0.681601	0.514213
C4B_2	Max_absorbance	0.204465	0.205668	0.681601	0.514213
C8G	HbA1c	0.20483	0.204844	0.681601	0.514213
COLEC11	HbA1c	0.2034	0.208091	0.681601	0.514213
CP	HbA1c	0.203976	0.206779	0.681601	0.514213
CPN1	Lysis50_time_sec	0.203338	0.208233	0.681601	0.514213
CRTAC1	Lag_time_sec	0.203244	0.208448	0.681601	0.514213
IGHG2	Lysis50_time_sec	-0.20345	0.207978	0.681601	0.514213
IGHV3-15	Lysis50_time_sec	-0.20423	0.206203	0.681601	0.514213
IGLL5	Clot_lysis_area	-0.20441	0.205795	0.681601	0.514213
ITIH2	Lysis50_time_sec	-0.20404	0.206641	0.681601	0.514213
JCHAIN	HbA1c	-0.20489	0.204712	0.681601	0.514213
PI16	Clot_lysis_area	-0.20408	0.206534	0.681601	0.514213

© Thieme. All rights reserved.

SERPINA6	Lag_time_sec	-0.20321	0.208522	0.681601	0.514213
A2M	eGDRWC	0.202086	0.211108	0.682261	0.514711
ALCAM	eGDRWC	-0.20297	0.20908	0.682261	0.514711
FBLN5	HbA1c	0.202032	0.211232	0.682261	0.514711
FGA	Lysis50_time_sec	0.202496	0.210164	0.682261	0.514711
IGKV1-13	eGDRWC	0.202718	0.209652	0.682261	0.514711
IGKV1-33	Lag_time_sec	0.201824	0.211712	0.682261	0.514711
IGKV4-1	Max_absorbance	0.201791	0.211789	0.682261	0.514711
IGLV3-27	Lag_time_sec	0.202449	0.210271	0.682261	0.514711
LCAT	Max_absorbance	-0.2023	0.210606	0.682261	0.514711
CD44	Clot_lysis_area	-0.20114	0.213305	0.686152	0.517647
FGB	Lysis50_time_sec	0.200837	0.214002	0.686234	0.517709
IGKV1-13	Clot_lysis_area	0.200728	0.214255	0.686234	0.517709
THBS4	Max_absorbance	0.20091	0.213832	0.686234	0.517709
C3	Max_absorbance	0.200182	0.215533	0.687606	0.518744
FCN2	Max_absorbance	0.20018	0.215537	0.687606	0.518744
FGB	Clot_lysis_area	0.200148	0.215611	0.687606	0.518744
PCYOX1	Max_absorbance	0.199881	0.216238	0.68862	0.519509
ANTXR1	Lysis50_time_sec	-0.19817	0.220267	0.689386	0.520087
APMAP	eGDRWC	0.199445	0.217261	0.689386	0.520087
APOB	eGDRWC	-0.19807	0.220505	0.689386	0.520087
APOC2	HbA1c	0.198303	0.21996	0.689386	0.520087
C2	eGDRWC	-0.19858	0.219297	0.689386	0.520087
CPN2	Clot_lysis_area	0.1991	0.218074	0.689386	0.520087
IGHV3-64	Lag_time_sec	-0.1982	0.220214	0.689386	0.520087
IGKJ1	Clot_lysis_area	-0.19808	0.220481	0.689386	0.520087
IGLV2-11	Lysis50_time_sec	-0.19845	0.219603	0.689386	0.520087
KLKB1	Lag_time_sec	-0.1992	0.217829	0.689386	0.520087
SPP2	Max_absorbance	-0.19843	0.219659	0.689386	0.520087
IGHV5-51	Lysis50_time_sec	-0.19747	0.221935	0.692644	0.522544
RNASE4	Max_absorbance	0.197376	0.222169	0.692644	0.522544
MBL2	eGDRWC	-0.19713	0.222752	0.692779	0.522647

PTPRJ	HbA1c	0.197097	0.222835	0.692779	0.522647
IGLV7-46	Max_absorbance	-0.19677	0.223622	0.693289	0.523031
A0A0G2JRQ6	Clot_lysis_area	-0.19586	0.225807	0.696185	0.525216
VCAM1	Lysis50_time_sec	0.195943	0.22561	0.696185	0.525216
IGLV2-11	Lag_time_sec	0.195339	0.227072	0.69719	0.525974
MBL2	Lysis50_time_sec	0.195529	0.226611	0.69719	0.525974
ITIH1	Max_absorbance	-0.1951	0.227658	0.697651	0.526322
PVR	Lysis50_time_sec	0.195019	0.227849	0.697651	0.526322
GAPDH	Lag_time_sec	0.194558	0.228972	0.700126	0.528189
C8B	eGDRWC	-0.19424	0.22975	0.700695	0.528618
IGKV2-24	Max_absorbance	-0.19422	0.229788	0.700695	0.528618
F11	Max_absorbance	0.19355	0.231441	0.704771	0.531693
CD44	eGDRWC	-0.1932	0.2323	0.706019	0.532635
LYVE1	Max_absorbance	-0.19313	0.232485	0.706019	0.532635
PTGDS	eGDRWC	0.192784	0.233327	0.707611	0.533836
IGHV3OR16-12	Max_absorbance	-0.19238	0.234329	0.708346	0.534391
IGLV1-47	Lag_time_sec	0.192383	0.234322	0.708346	0.534391
CD14	Clot_lysis_area	0.19145	0.23664	0.709086	0.534949
IGKV1-8	HbA1c	-0.19154	0.236428	0.709086	0.534949
IGKV2D-24	Lag_time_sec	0.191655	0.236129	0.709086	0.534949
MST1	HbA1c	-0.19162	0.236218	0.709086	0.534949
PGLYRP2	Clot_lysis_area	-0.19144	0.236666	0.709086	0.534949
PROC	Lysis50_time_sec	-0.19143	0.236681	0.709086	0.534949
GC	Lag_time_sec	-0.1908	0.238266	0.711048	0.536429
IGF2R	Clot_lysis_area	0.190789	0.238294	0.711048	0.536429
MAN1A1	Clot_lysis_area	0.190479	0.239073	0.712417	0.537462
IGHV1-18	Max_absorbance	-0.19016	0.23987	0.713227	0.538073
AGT	Clot_lysis_area	0.189541	0.241437	0.714608	0.539114
IGHV1-2	eGDRWC	0.189335	0.241958	0.714608	0.539114
IGHV2-5	Lag_time_sec	0.189294	0.242061	0.714608	0.539114
IGKV2-40	Clot_lysis_area	0.18917	0.242376	0.714608	0.539114
IGKV3D-20	Lag_time_sec	0.189746	0.240917	0.714608	0.539114

IGHV1OR15-1	eGDRWC	0.188884	0.243101	0.714851	0.539298
SERPINA7	Lysis50_time_sec	0.188978	0.242864	0.714851	0.539298
AHSG	Clot_lysis_area	-0.18805	0.24523	0.717539	0.541326
IGLV3-9	Lysis50_time_sec	-0.18825	0.244708	0.717539	0.541326
LRP1	Lag_time_sec	0.188082	0.245145	0.717539	0.541326
PROCR	Lysis50_time_sec	-0.18802	0.245304	0.717539	0.541326
APOA2	Clot_lysis_area	-0.18739	0.246919	0.719144	0.542537
C1R	Max_absorbance	0.187301	0.247145	0.719144	0.542537
ATRN	Lysis50_time_sec	0.186585	0.24899	0.723308	0.545678
SERPINA6	eGDRWC	-0.18649	0.249226	0.723308	0.545678
FETUB	eGDRWC	-0.186	0.250493	0.725848	0.547595
IGKV3-11	HbA1c	-0.1859	0.250754	0.725848	0.547595
ANTXR1	Clot_lysis_area	-0.18553	0.251718	0.72618	0.547845
HBD	HbA1c	0.185339	0.252223	0.72618	0.547845
IGKV1-16	Max_absorbance	-0.18559	0.251565	0.72618	0.547845
LYVE1	Lysis50_time_sec	-0.18511	0.252821	0.72618	0.547845
SERPINA4	eGDRWC	-0.18511	0.252826	0.72618	0.547845
FCGBP	eGDRWC	-0.18491	0.253339	0.726454	0.548052
IGHV3-43	Clot_lysis_area	-0.1847	0.2539	0.726454	0.548052
LGALS3BP	Lag_time_sec	-0.18476	0.25372	0.726454	0.548052
LYVE1	HbA1c	-0.18429	0.254967	0.726706	0.548242
PON1	Max_absorbance	0.184391	0.254698	0.726706	0.548242
SAA4	Lysis50_time_sec	0.184451	0.25454	0.726706	0.548242
GSN	Max_absorbance	-0.18377	0.256339	0.728749	0.549783
MASP1	HbA1c	0.183389	0.257334	0.730644	0.551213
IGHV3OR16-9	eGDRWC	0.183108	0.258078	0.731823	0.552102
APOL1	Clot_lysis_area	0.182458	0.2598	0.734835	0.554374
F5	Max_absorbance	0.182088	0.260783	0.73668	0.555766
ABCB9	Max_absorbance	-0.18005	0.266244	0.737286	0.556223
APOC2	eGDRWC	-0.1801	0.266102	0.737286	0.556223
C1S	Max_absorbance	0.179729	0.267117	0.737286	0.556223
C4B_2	Lag_time_sec	0.181263	0.262986	0.737286	0.556223

© Thieme. All rights reserved.

C8A	Clot_lysis_area	0.179717	0.26715	0.737286	0.556223
C8B	Lysis50_time_sec	0.180907	0.263941	0.737286	0.556223
CFHR1	Max_absorbance	0.180452	0.265165	0.737286	0.556223
HBD	Lag_time_sec	0.180097	0.266121	0.737286	0.556223
IGHV1OR15-1	Max_absorbance	-0.18076	0.264348	0.737286	0.556223
IGHV3OR16-9	HbA1c	-0.17966	0.267291	0.737286	0.556223
IGKV1-39	eGDRWC	0.181442	0.262508	0.737286	0.556223
IGKV1D-16	Lag_time_sec	-0.18167	0.261896	0.737286	0.556223
IGKV3D-15	Lysis50_time_sec	-0.17987	0.266741	0.737286	0.556223
MASP1	Lag_time_sec	-0.18127	0.262962	0.737286	0.556223
MBL2	HbA1c	0.181277	0.262951	0.737286	0.556223
HGFAC	Lag_time_sec	-0.17931	0.268263	0.73905	0.557554
CD44	Lysis50_time_sec	-0.17865	0.270052	0.739404	0.557821
IGFALS	Lysis50_time_sec	0.178674	0.26998	0.739404	0.557821
IGFBP3	eGDRWC	-0.17865	0.270042	0.739404	0.557821
IGLV7-43	Lag_time_sec	0.178995	0.269106	0.739404	0.557821
QSOX1	eGDRWC	-0.17866	0.270019	0.739404	0.557821
HSP90B1	Lag_time_sec	-0.17794	0.271995	0.742897	0.560457
ADAMTS13	eGDRWC	0.177005	0.274554	0.743576	0.560969
B2M	Clot_lysis_area	-0.17654	0.275829	0.743576	0.560969
BCHE	Max_absorbance	0.176691	0.27542	0.743576	0.560969
CFHR4	Max_absorbance	0.177144	0.274171	0.743576	0.560969
FCN2	Lysis50_time_sec	-0.17651	0.275918	0.743576	0.560969
HSPG2	Clot_lysis_area	-0.17755	0.273063	0.743576	0.560969
IGHV2-5	HbA1c	0.177465	0.27329	0.743576	0.560969
IGHV3-38	Lysis50_time_sec	-0.17685	0.274973	0.743576	0.560969
IGKV1-17	Max_absorbance	0.177627	0.272844	0.743576	0.560969
IL1RAP	Max_absorbance	-0.17717	0.274089	0.743576	0.560969
ITIH3	HbA1c	0.176978	0.274627	0.743576	0.560969
IGHV3-43	Lag_time_sec	0.176229	0.276698	0.744777	0.561875
SERPINC1	Clot_lysis_area	-0.17591	0.277573	0.746229	0.56297
A2M	Lag_time_sec	0.174138	0.282526	0.74997	0.565793

© Thieme. All rights reserved.

AFM	HbA1c	0.173002	0.285728	0.74997	0.565793
APOA1	HbA1c	-0.17332	0.284829	0.74997	0.565793
APOA4	Clot_lysis_area	-0.17289	0.286048	0.74997	0.565793
C1QC	Lag_time_sec	0.175023	0.280049	0.74997	0.565793
C4BPA	Lag_time_sec	-0.17384	0.283352	0.74997	0.565793
C6	HbA1c	0.173349	0.284747	0.74997	0.565793
CA1	Max_absorbance	0.172961	0.285845	0.74997	0.565793
CAMP	Lag_time_sec	0.174729	0.280872	0.74997	0.565793
COL6A3	Clot_lysis_area	0.172956	0.285858	0.74997	0.565793
DCD	HbA1c	-0.17423	0.282258	0.74997	0.565793
FGA	Lag_time_sec	-0.17359	0.284081	0.74997	0.565793
HPR	HbA1c	0.175114	0.279795	0.74997	0.565793
IGHV3-38-3	Clot_lysis_area	-0.17387	0.283272	0.74997	0.565793
IGKV1-8	Max_absorbance	0.173747	0.283627	0.74997	0.565793
IGKV2D-30	Clot_lysis_area	0.173214	0.285128	0.74997	0.565793
IGKV3D-20	Max_absorbance	-0.17468	0.281019	0.74997	0.565793
IGLV3-21	HbA1c	-0.17496	0.280227	0.74997	0.565793
PIP	Lag_time_sec	0.172826	0.286226	0.74997	0.565793
SERPING1	HbA1c	0.172654	0.286714	0.74997	0.565793
HABP2	HbA1c	0.172352	0.287569	0.750445	0.566151
IGKV2D-30	HbA1c	-0.17242	0.287367	0.750445	0.566151
GP1BA	eGDRWC	-0.17191	0.288835	0.752866	0.567977
HSPA6	Clot_lysis_area	0.171639	0.289599	0.753973	0.568812
HSP90B1	eGDRWC	-0.17119	0.290893	0.756459	0.570688
APOC1	HbA1c	-0.17106	0.291242	0.75648	0.570704
IGHV1-69	eGDRWC	0.170859	0.291829	0.757122	0.571188
F10	Lysis50_time_sec	0.170568	0.292664	0.758405	0.572156
ADAMTS13	Lag_time_sec	-0.16776	0.300797	0.758889	0.572521
APCS	eGDRWC	-0.16967	0.295253	0.758889	0.572521
APOA2	HbA1c	0.168277	0.299291	0.758889	0.572521
APOH	Lag_time_sec	0.167879	0.300451	0.758889	0.572521
CFHR1	eGDRWC	0.167856	0.30052	0.758889	0.572521

CLU	Max_absorbance	0.169556	0.295579	0.758889	0.572521
F5	Lag_time_sec	-0.16939	0.296051	0.758889	0.572521
GPLD1	Max_absorbance	-0.16797	0.300187	0.758889	0.572521
IGHA1	HbA1c	-0.16867	0.298146	0.758889	0.572521
IGHG2	HbA1c	-0.1685	0.29863	0.758889	0.572521
IGHV3-15	Max_absorbance	0.16938	0.296088	0.758889	0.572521
IGHV3OR15-7	Lag_time_sec	0.169121	0.29684	0.758889	0.572521
IGKV1-16	Clot_lysis_area	-0.16774	0.300862	0.758889	0.572521
IGLC3	Lag_time_sec	0.167798	0.300691	0.758889	0.572521
IGLV5-37	eGDRWC	0.168983	0.297239	0.758889	0.572521
ORM2	HbA1c	0.168094	0.299826	0.758889	0.572521
PI16	Max_absorbance	-0.17005	0.294145	0.758889	0.572521
PRR4	Clot_lysis_area	0.169966	0.294395	0.758889	0.572521
SERPING1	Lag_time_sec	-0.16937	0.296123	0.758889	0.572521
TNXB	HbA1c	-0.16768	0.301033	0.758889	0.572521
VTN	eGDRWC	-0.16836	0.299039	0.758889	0.572521
ANGPTL6	Max_absorbance	0.166455	0.304631	0.759118	0.572694
B4GALT1	HbA1c	0.167313	0.302108	0.759118	0.572694
CFI	Clot_lysis_area	0.166604	0.304192	0.759118	0.572694
COLEC11	Clot_lysis_area	-0.16626	0.305216	0.759118	0.572694
FBLN5	Lag_time_sec	0.166474	0.304575	0.759118	0.572694
IGHA1	Lag_time_sec	0.16721	0.302412	0.759118	0.572694
IGLV2-8	HbA1c	-0.16717	0.30253	0.759118	0.572694
LCAT	HbA1c	-0.16629	0.305129	0.759118	0.572694
MCAM	HbA1c	-0.16653	0.304421	0.759118	0.572694
NRP1	Lag_time_sec	0.167318	0.302093	0.759118	0.572694
IGKV3-20	Clot_lysis_area	-0.16599	0.306003	0.760226	0.57353
A1BG	Max_absorbance	-0.16164	0.319042	0.760411	0.573669
A2M	HbA1c	0.163039	0.314802	0.760411	0.573669
AGT	Max_absorbance	0.165008	0.308914	0.760411	0.573669
AGT	Lysis50_time_sec	0.162179	0.317395	0.760411	0.573669
ANGPTL6	Lysis50_time_sec	0.157138	0.332871	0.760411	0.573669

ANTXR1	HbA1c	0.1605	0.322498	0.760411	0.573669
APOA1	Lysis50_time_sec	-0.16426	0.311155	0.760411	0.573669
APOB	Lysis50_time_sec	0.162602	0.31612	0.760411	0.573669
BTD	Lysis50_time_sec	0.159472	0.325649	0.760411	0.573669
C4A	Lag_time_sec	0.161619	0.319091	0.760411	0.573669
CAMP	eGDRWC	0.15545	0.338156	0.760411	0.573669
CLEC3B	Clot_lysis_area	0.157515	0.331699	0.760411	0.573669
CTSD	HbA1c	-0.15788	0.330563	0.760411	0.573669
CTSD	Max_absorbance	-0.15511	0.339213	0.760411	0.573669
CTSD	Lysis50_time_sec	0.159802	0.324636	0.760411	0.573669
DSG1	Lysis50_time_sec	0.161403	0.319748	0.760411	0.573669
F11	eGDRWC	-0.16082	0.321509	0.760411	0.573669
F13B	Clot_lysis_area	0.156261	0.33561	0.760411	0.573669
F5	Lysis50_time_sec	0.156908	0.333589	0.760411	0.573669
FBLN1	Lag_time_sec	0.158315	0.329217	0.760411	0.573669
FBLN1	Max_absorbance	-0.15971	0.324929	0.760411	0.573669
FBLN5	eGDRWC	-0.16522	0.308287	0.760411	0.573669
FCGR3A	HbA1c	-0.15583	0.33697	0.760411	0.573669
FCN3	Lysis50_time_sec	0.15886	0.327533	0.760411	0.573669
FETUB	HbA1c	0.159918	0.32428	0.760411	0.573669
FGB	Lag_time_sec	-0.16568	0.306932	0.760411	0.573669
HBA2	eGDRWC	-0.16475	0.309673	0.760411	0.573669
HPR	Lag_time_sec	-0.16217	0.317419	0.760411	0.573669
HPX	Lag_time_sec	-0.16026	0.323244	0.760411	0.573669
HRG	Clot_lysis_area	-0.15889	0.327454	0.760411	0.573669
HSP90B1	Max_absorbance	0.155235	0.338833	0.760411	0.573669
HSP90B1	Clot_lysis_area	0.155693	0.337392	0.760411	0.573669
ICOSLG	eGDRWC	0.155261	0.338751	0.760411	0.573669
ICOSLG	Lag_time_sec	-0.15954	0.325452	0.760411	0.573669
IGFBP3	Lysis50_time_sec	-0.16215	0.317489	0.760411	0.573669
IGHA1	Lysis50_time_sec	-0.16457	0.310211	0.760411	0.573669
IGHG3	HbA1c	0.164818	0.309477	0.760411	0.573669

IGHM	eGDRWC	0.16036	0.322927	0.760411	0.573669
IGHV1-18	Lysis50_time_sec	-0.16532	0.30799	0.760411	0.573669
IGHV1-2	Lysis50_time_sec	-0.16198	0.317998	0.760411	0.573669
IGHV2-26	Max_absorbance	0.164688	0.309866	0.760411	0.573669
IGHV3-13	Max_absorbance	0.161826	0.318464	0.760411	0.573669
IGHV3-38	Max_absorbance	0.156089	0.336148	0.760411	0.573669
IGHV3-43	eGDRWC	0.162892	0.315244	0.760411	0.573669
IGHV3-64	Lysis50_time_sec	0.155834	0.33695	0.760411	0.573669
IGHV3-64D	HbA1c	-0.15816	0.329692	0.760411	0.573669
IGHV3-64D	Lag_time_sec	0.160074	0.323803	0.760411	0.573669
IGHV3OR15-7	eGDRWC	-0.15677	0.334022	0.760411	0.573669
IGHV5-51	eGDRWC	0.158595	0.328351	0.760411	0.573669
IGHV5-51	Max_absorbance	0.16462	0.310069	0.760411	0.573669
IGKV1-12	eGDRWC	0.162598	0.316131	0.760411	0.573669
IGKV2-30	Clot_lysis_area	0.158488	0.328681	0.760411	0.573669
IGLV2-8	Lag_time_sec	0.157855	0.33064	0.760411	0.573669
IGLV3-19	HbA1c	0.15989	0.324364	0.760411	0.573669
IGLV5-45	Lysis50_time_sec	0.162061	0.317754	0.760411	0.573669
IGLV9-49	Max_absorbance	0.159135	0.326684	0.760411	0.573669
ITIH2	eGDRWC	-0.15624	0.335663	0.760411	0.573669
ITIH3	Clot_lysis_area	0.156264	0.335601	0.760411	0.573669
JCHAIN	Lag_time_sec	0.15819	0.329603	0.760411	0.573669
JCHAIN	Lysis50_time_sec	-0.15896	0.32721	0.760411	0.573669
LBP	Lag_time_sec	-0.15797	0.330293	0.760411	0.573669
LBP	Lysis50_time_sec	0.156892	0.333637	0.760411	0.573669
LCAT	Clot_lysis_area	0.156239	0.335678	0.760411	0.573669
LCP1	Max_absorbance	0.161359	0.31988	0.760411	0.573669
LRP1	eGDRWC	-0.16273	0.315721	0.760411	0.573669
MAN1A1	Max_absorbance	0.161579	0.319213	0.760411	0.573669
NID1	Lag_time_sec	-0.15518	0.339016	0.760411	0.573669
ORM1	eGDRWC	-0.16285	0.315378	0.760411	0.573669
ORM1	Clot_lysis_area	0.158856	0.327544	0.760411	0.573669

PIP	Lysis50_time_sec	0.155221	0.338877	0.760411	0.573669
PLTP	Lysis50_time_sec	-0.15899	0.327144	0.760411	0.573669
PLXDC2	Max_absorbance	0.15864	0.328213	0.760411	0.573669
PLXDC2	Lysis50_time_sec	-0.1631	0.314624	0.760411	0.573669
PLXDC2	Clot_lysis_area	0.164502	0.310421	0.760411	0.573669
PROC	Clot_lysis_area	-0.16046	0.322622	0.760411	0.573669
PROS1	Clot_lysis_area	0.15783	0.330718	0.760411	0.573669
PROZ	Max_absorbance	-0.16225	0.317185	0.760411	0.573669
PVR	eGDRWC	0.15845	0.328799	0.760411	0.573669
SELENOP	eGDRWC	-0.16104	0.320863	0.760411	0.573669
SELL	Max_absorbance	-0.1641	0.311619	0.760411	0.573669
SERPINA5	Lag_time_sec	-0.1603	0.323114	0.760411	0.573669
SERPINA7	Max_absorbance	-0.15903	0.327018	0.760411	0.573669
SERPINC1	Lysis50_time_sec	-0.16246	0.31656	0.760411	0.573669
SERPIND1	HbA1c	0.160374	0.322885	0.760411	0.573669
SERPINF1	Clot_lysis_area	-0.16363	0.313024	0.760411	0.573669
SHBG	Lag_time_sec	0.157622	0.331366	0.760411	0.573669
VTN	Lysis50_time_sec	0.156439	0.335052	0.760411	0.573669
RNASE4	Lysis50_time_sec	-0.15485	0.340036	0.761488	0.574482
CA1	HbA1c	0.154666	0.340628	0.762049	0.574905
APOH	Lysis50_time_sec	-0.15376	0.343483	0.762118	0.574958
C4A	HbA1c	0.153485	0.344373	0.762118	0.574958
C8B	Max_absorbance	0.154167	0.342206	0.762118	0.574958
GLIPR2	Lysis50_time_sec	-0.15398	0.342785	0.762118	0.574958
ICOSLG	Clot_lysis_area	-0.15361	0.343986	0.762118	0.574958
IGHG4	Lag_time_sec	0.153721	0.343623	0.762118	0.574958
IGHV1-46	Lysis50_time_sec	-0.15358	0.344057	0.762118	0.574958
IGKC	Lysis50_time_sec	-0.15328	0.345026	0.762118	0.574958
IGLV9-49	Lysis50_time_sec	-0.15325	0.34511	0.762118	0.574958
RARRES2	Clot_lysis_area	0.153633	0.343903	0.762118	0.574958
SERPINC1	Max_absorbance	-0.15392	0.342991	0.762118	0.574958
B2M	eGDRWC	-0.15264	0.347059	0.764064	0.576425

ECM1	Clot_lysis_area	0.152567	0.3473	0.764064	0.576425
F7	Max_absorbance	0.152265	0.348266	0.764064	0.576425
GLIPR2	eGDRWC	0.152845	0.346413	0.764064	0.576425
HBA2	Lag_time_sec	0.152386	0.34788	0.764064	0.576425
HSPG2	HbA1c	-0.15223	0.348394	0.764064	0.576425
PCYOX1	eGDRWC	0.152459	0.347646	0.764064	0.576425
CFHR5	Lysis50_time_sec	0.151946	0.349291	0.764522	0.576771
GP1BA	Clot_lysis_area	-0.15188	0.349517	0.764522	0.576771
IGHV3-43	HbA1c	-0.15173	0.349977	0.764522	0.576771
IGLL1	Max_absorbance	0.151804	0.349746	0.764522	0.576771
COL6A3	HbA1c	-0.15115	0.35184	0.765357	0.577401
EFEMP1	Max_absorbance	0.151443	0.350907	0.765357	0.577401
IGKV2-40	HbA1c	0.151143	0.351871	0.765357	0.577401
IGLV3-27	Max_absorbance	0.151372	0.351135	0.765357	0.577401
QSOX1	HbA1c	-0.15108	0.352078	0.765357	0.577401
SERPINA5	Max_absorbance	-0.15096	0.352456	0.765431	0.577457
IGHV3-64	HbA1c	0.150575	0.353705	0.765957	0.577853
LYZ	Lysis50_time_sec	0.150528	0.353857	0.765957	0.577853
PROZ	Clot_lysis_area	-0.15047	0.354056	0.765957	0.577853
SERPINA7	HbA1c	0.150461	0.354074	0.765957	0.577853
C1S	HbA1c	0.150294	0.354617	0.766385	0.578177
IGHV1-45	Lag_time_sec	-0.1499	0.35588	0.76837	0.579674
A0A0J9YY99	HbA1c	0.149023	0.358747	0.770481	0.581266
CPQ	eGDRWC	-0.14907	0.358607	0.770481	0.581266
HSP90B1	HbA1c	-0.14889	0.359176	0.770481	0.581266
C3	Clot_lysis_area	0.148357	0.360923	0.773258	0.583361
CD44	Max_absorbance	-0.14812	0.361699	0.774176	0.584054
IGHV1-45	HbA1c	0.147863	0.362542	0.774489	0.58429
IGKV1D-16	Lysis50_time_sec	-0.14788	0.362501	0.774489	0.58429
APOC4	Lysis50_time_sec	0.147629	0.363311	0.775389	0.584969
LPA	Lag_time_sec	-0.14739	0.364092	0.776311	0.585665
PLTP	Clot_lysis_area	0.147114	0.365005	0.776957	0.586152

© Thieme. All rights reserved.

CPB2	Lag_time_sec	-0.14678	0.366101	0.777669	0.586689
PLG	Lag_time_sec	-0.14677	0.366126	0.777669	0.586689
F13A1	eGDRWC	-0.14593	0.368905	0.77985	0.588335
HGFAC	Lysis50_time_sec	-0.1462	0.368029	0.77985	0.588335
HPX	HbA1c	-0.14605	0.36853	0.77985	0.588335
IGFBP3	Clot_lysis_area	0.146301	0.367689	0.77985	0.588335
IGHV3-13	Clot_lysis_area	0.145954	0.36884	0.77985	0.588335
ABCB9	Lysis50_time_sec	-0.14564	0.369897	0.780464	0.588798
PGLYRP2	Lag_time_sec	0.145671	0.36978	0.780464	0.588798
IGHV1-69	HbA1c	-0.14552	0.370291	0.780557	0.588868
APOE	Lysis50_time_sec	0.145175	0.371428	0.780735	0.589002
IGHG2	eGDRWC	0.145191	0.371376	0.780735	0.589002
IGKV1-5	Max_absorbance	0.145348	0.370852	0.780735	0.589002
AHSG	HbA1c	-0.14466	0.373149	0.781401	0.589504
F2	Max_absorbance	-0.14471	0.372987	0.781401	0.589504
IGLV2-11	Clot_lysis_area	-0.14483	0.372572	0.781401	0.589504
KNG1	Clot_lysis_area	0.144903	0.372335	0.781401	0.589504
HSPA5	Max_absorbance	-0.14438	0.374066	0.782262	0.590154
IGLV2-11	Max_absorbance	-0.14433	0.374263	0.782262	0.590154
FBLN1	Clot_lysis_area	0.143978	0.375427	0.78396	0.591435
SERPINA10	Max_absorbance	0.143865	0.375807	0.784017	0.591479
APOC4	Max_absorbance	0.14344	0.377234	0.785522	0.592614
SERPINA5	HbA1c	0.143454	0.377185	0.785522	0.592614
APOC3	Lag_time_sec	-0.14323	0.377952	0.786281	0.593186
IGHG4	eGDRWC	0.14305	0.378546	0.786338	0.593229
PLG	Lysis50_time_sec	0.143008	0.378686	0.786338	0.593229
IGLV3-21	Max_absorbance	-0.1426	0.380056	0.788448	0.594821
APOF	HbA1c	0.142196	0.381429	0.789341	0.595495
IGHV3-7	Lysis50_time_sec	-0.14225	0.38126	0.789341	0.595495
IGLV1-51	Lag_time_sec	0.14216	0.38155	0.789341	0.595495
CD5L	Clot_lysis_area	0.141863	0.382555	0.789545	0.595649
DSG1	Lag_time_sec	-0.14186	0.382556	0.789545	0.595649

SERPINA5	Lysis50_time_sec	0.141816	0.382713	0.789545	0.595649
F12	Max_absorbance	-0.14128	0.384549	0.789982	0.595978
IGHV1-24	Lag_time_sec	0.141474	0.383876	0.789982	0.595978
IGHV1OR15-1	Lag_time_sec	0.140606	0.386829	0.789982	0.595978
IGKV2-24	HbA1c	-0.14079	0.38621	0.789982	0.595978
IGLV1-36	Max_absorbance	0.140921	0.385754	0.789982	0.595978
LRG1	eGDRWC	-0.14147	0.383904	0.789982	0.595978
TTR	eGDRWC	-0.1411	0.385139	0.789982	0.595978
HBB	Lag_time_sec	0.139906	0.389221	0.791962	0.597472
IGHV1-24	Max_absorbance	0.139909	0.389211	0.791962	0.597472
IL1RAP	HbA1c	0.140177	0.388293	0.791962	0.597472
SELL	Lysis50_time_sec	0.140068	0.388668	0.791962	0.597472
CASP14	Lysis50_time_sec	-0.13933	0.391202	0.795266	0.599965
C4A	Lysis50_time_sec	0.138745	0.393209	0.796117	0.600607
IGHV3-64D	Lysis50_time_sec	-0.13899	0.392355	0.796117	0.600607
IGHV3-74	Lag_time_sec	0.138944	0.392521	0.796117	0.600607
IGLV2-18	Max_absorbance	0.138687	0.393408	0.796117	0.600607
LUM	eGDRWC	-0.13848	0.394124	0.796117	0.600607
IGLV2-18	Lysis50_time_sec	0.138354	0.394557	0.796267	0.60072
IGKV6D-21	eGDRWC	-0.13824	0.394935	0.796309	0.600752
IGHV1-69	Max_absorbance	-0.13796	0.395914	0.79756	0.601696
CLU	Lysis50_time_sec	0.137584	0.397219	0.79943	0.603106
IGHV4-34	eGDRWC	0.137485	0.39756	0.79943	0.603106
AMBP	eGDRWC	-0.1371	0.398897	0.801393	0.604587
ANTXR1	eGDRWC	-0.13698	0.399296	0.801473	0.604648
GLIPR2	Max_absorbance	-0.13647	0.40107	0.803584	0.60624
IGHV3-49	eGDRWC	0.13654	0.400844	0.803584	0.60624
C1QB	Clot_lysis_area	0.135992	0.402754	0.803762	0.606374
C1S	Lag_time_sec	-0.1354	0.40481	0.803762	0.606374
C8G	eGDRWC	-0.13504	0.406084	0.803762	0.606374
CPQ	Lag_time_sec	-0.13581	0.403394	0.803762	0.606374
DBH	eGDRWC	-0.13394	0.40995	0.803762	0.606374

GPX3	Max_absorbance	-0.13458	0.407709	0.803762	0.606374
GSN	Lag_time_sec	0.135165	0.405647	0.803762	0.606374
HBA2	Lysis50_time_sec	0.134826	0.406838	0.803762	0.606374
IGHV1-3	eGDRWC	-0.13507	0.405984	0.803762	0.606374
IGHV3OR16-12	eGDRWC	0.133875	0.410186	0.803762	0.606374
IGKV1-27	Lag_time_sec	-0.13536	0.404973	0.803762	0.606374
IGLL1	Clot_lysis_area	0.135763	0.403553	0.803762	0.606374
IGLV2-8	eGDRWC	0.134727	0.407184	0.803762	0.606374
IGLV3-9	eGDRWC	0.136007	0.4027	0.803762	0.606374
KNG1	Lag_time_sec	-0.13501	0.406196	0.803762	0.606374
MCAM	Max_absorbance	0.134611	0.407592	0.803762	0.606374
NID1	Clot_lysis_area	0.134067	0.409508	0.803762	0.606374
SAA2	eGDRWC	-0.13419	0.409091	0.803762	0.606374
SERPIND1	eGDRWC	-0.13422	0.408963	0.803762	0.606374
SPP2	Lag_time_sec	0.134972	0.406323	0.803762	0.606374
TGFBI	Clot_lysis_area	0.13505	0.406049	0.803762	0.606374
VCAM1	HbA1c	-0.13513	0.405764	0.803762	0.606374
VTN	HbA1c	0.134893	0.406602	0.803762	0.606374
APOC3	eGDRWC	0.133455	0.411669	0.805064	0.607357
C7	Lysis50_time_sec	0.133175	0.412659	0.805064	0.607357
IGHM	Max_absorbance	-0.1334	0.41186	0.805064	0.607357
MAN1A1	eGDRWC	-0.13332	0.412149	0.805064	0.607357
PTGDS	Max_absorbance	-0.13323	0.412447	0.805064	0.607357
VASN	Lag_time_sec	0.132956	0.413436	0.805875	0.607969
APMAP	HbA1c	0.132697	0.414354	0.806957	0.608785
COMP	eGDRWC	-0.13256	0.414848	0.807214	0.608978
ABCB9	HbA1c	0.132018	0.416767	0.807903	0.609498
CDH13	Clot_lysis_area	-0.13224	0.415983	0.807903	0.609498
CHL1	Max_absorbance	-0.13128	0.419417	0.807903	0.609498
F12	eGDRWC	-0.13098	0.42047	0.807903	0.609498
FCGR3B	Max_absorbance	0.131779	0.417618	0.807903	0.609498
FETUB	Lysis50_time_sec	0.13128	0.419401	0.807903	0.609498

© Thieme. All rights reserved.

IGKV1-13	Lysis50_time_sec	-0.13097	0.420497	0.807903	0.609498
IGKV2-30	eGDRWC	-0.1315	0.418621	0.807903	0.609498
IGLV2-8	Lysis50_time_sec	0.130931	0.420647	0.807903	0.609498
IGLV5-39	Lysis50_time_sec	-0.13193	0.41707	0.807903	0.609498
PRSS3	Lag_time_sec	0.13104	0.420258	0.807903	0.609498
SELENOP	Clot_lysis_area	-0.1322	0.416105	0.807903	0.609498
SPP2	Lysis50_time_sec	0.131622	0.418178	0.807903	0.609498
C8A	Lysis50_time_sec	0.130379	0.422626	0.809146	0.610436
HPR	Clot_lysis_area	0.130345	0.422748	0.809146	0.610436
IGHV3-72	Max_absorbance	0.130406	0.42253	0.809146	0.610436
HSPG2	eGDRWC	0.129972	0.424089	0.81032	0.611322
IGHV3-9	Lysis50_time_sec	0.129857	0.424503	0.810415	0.611393
IGHV3-7	Lag_time_sec	0.129547	0.42562	0.811156	0.611953
PON3	Lag_time_sec	0.129605	0.425409	0.811156	0.611953
CTSD	Clot_lysis_area	-0.12793	0.431461	0.811531	0.612236
EFEMP1	HbA1c	-0.12794	0.431437	0.811531	0.612236
F13B	HbA1c	-0.12887	0.428051	0.811531	0.612236
GLIPR2	Lag_time_sec	-0.1279	0.431582	0.811531	0.612236
HP	HbA1c	0.128288	0.43017	0.811531	0.612236
IGHV3-13	Lysis50_time_sec	-0.12831	0.430105	0.811531	0.612236
IGKJ1	Lysis50_time_sec	-0.12821	0.430434	0.811531	0.612236
IGKV1-37	Lag_time_sec	-0.12846	0.429546	0.811531	0.612236
IGLV8-61	Max_absorbance	-0.12788	0.43165	0.811531	0.612236
LDHA	Lag_time_sec	0.128708	0.428649	0.811531	0.612236
LPA	eGDRWC	-0.12904	0.427432	0.811531	0.612236
LTF	Clot_lysis_area	0.128742	0.428522	0.811531	0.612236
TGFBI	HbA1c	-0.12789	0.431601	0.811531	0.612236
PROCR	Clot_lysis_area	-0.12773	0.432186	0.811854	0.612479
IGKV1-27	Max_absorbance	0.127455	0.433193	0.812374	0.612871
CLEC3B	eGDRWC	-0.12727	0.433858	0.812937	0.613296
ALCAM	Max_absorbance	-0.12217	0.45266	0.815607	0.615311
APOA1	eGDRWC	0.121928	0.45356	0.815607	0.615311

APOC2	Lag_time_sec	0.122902	0.449935	0.815607	0.615311
ART3	HbA1c	0.12582	0.439166	0.815607	0.615311
C1QB	eGDRWC	-0.12311	0.449161	0.815607	0.615311
C1QB	Max_absorbance	0.126728	0.435844	0.815607	0.615311
C1S	Lysis50_time_sec	0.125891	0.438906	0.815607	0.615311
C8A	Max_absorbance	-0.12549	0.440386	0.815607	0.615311
CASP14	eGDRWC	0.122066	0.453047	0.815607	0.615311
CFHR4	Clot_lysis_area	0.122235	0.452416	0.815607	0.615311
CFI	Lag_time_sec	-0.12272	0.450609	0.815607	0.615311
CPB2	Clot_lysis_area	0.122569	0.451172	0.815607	0.615311
CPN1	Lag_time_sec	0.124024	0.445776	0.815607	0.615311
CPN2	Max_absorbance	0.123598	0.447352	0.815607	0.615311
CTSD	eGDRWC	0.123836	0.446472	0.815607	0.615311
F13A1	Lysis50_time_sec	0.122482	0.451497	0.815607	0.615311
FCN2	Lag_time_sec	0.124469	0.444134	0.815607	0.615311
GAPDH	HbA1c	-0.12325	0.448653	0.815607	0.615311
GP1BA	HbA1c	0.123624	0.447257	0.815607	0.615311
GPX3	Lysis50_time_sec	-0.1258	0.439247	0.815607	0.615311
GPX3	Clot_lysis_area	-0.12215	0.452736	0.815607	0.615311
HPR	Lysis50_time_sec	0.125146	0.441641	0.815607	0.615311
HPX	Lysis50_time_sec	0.123402	0.44808	0.815607	0.615311
IGHA2	Lysis50_time_sec	0.122634	0.450931	0.815607	0.615311
IGHD	eGDRWC	0.126276	0.437494	0.815607	0.615311
IGHM	Clot_lysis_area	0.12296	0.449719	0.815607	0.615311
IGKV1-6	eGDRWC	0.12239	0.451838	0.815607	0.615311
IGKV2-40	Lysis50_time_sec	-0.12581	0.439209	0.815607	0.615311
IGKV2D-30	eGDRWC	0.123359	0.448241	0.815607	0.615311
IGKV3-7	Lag_time_sec	0.126172	0.437874	0.815607	0.615311
IGLC7	Clot_lysis_area	-0.12341	0.448049	0.815607	0.615311
IGLV1-36	eGDRWC	0.122856	0.450107	0.815607	0.615311
IGLV1-47	Max_absorbance	-0.12295	0.449759	0.815607	0.615311
IGLV3-10	Clot_lysis_area	-0.12618	0.437861	0.815607	0.615311

IGLV3-21	Lag_time_sec	-0.12266	0.45084	0.815607	0.615311
LPA	Lysis50_time_sec	-0.12281	0.450272	0.815607	0.615311
MASP1	Lysis50_time_sec	0.12531	0.441037	0.815607	0.615311
NRP1	Max_absorbance	-0.12192	0.453604	0.815607	0.615311
ORM1	Lysis50_time_sec	0.124532	0.443899	0.815607	0.615311
PVR	HbA1c	-0.12629	0.437435	0.815607	0.615311
RARRES2	HbA1c	-0.12462	0.443564	0.815607	0.615311
SAA4	Lag_time_sec	-0.12532	0.441007	0.815607	0.615311
SERPINF2	Lag_time_sec	0.125735	0.439478	0.815607	0.615311
BTD	Clot_lysis_area	0.121447	0.455357	0.815744	0.615414
IGHD	Clot_lysis_area	0.121406	0.455512	0.815744	0.615414
IGKV2-30	Lag_time_sec	0.121449	0.455349	0.815744	0.615414
PON3	HbA1c	0.121541	0.455005	0.815744	0.615414
ALDOA	Clot_lysis_area	-0.1207	0.458147	0.817175	0.616494
CD14	Lysis50_time_sec	0.120875	0.457502	0.817175	0.616494
CPB2	Max_absorbance	0.120707	0.45813	0.817175	0.616494
IGKV1-8	Clot_lysis_area	0.120825	0.457687	0.817175	0.616494
SELENOP	Lysis50_time_sec	0.120248	0.459853	0.819001	0.617871
IGKJ1	HbA1c	-0.11868	0.465768	0.828114	0.624746
CAMP	Clot_lysis_area	0.118158	0.467745	0.829289	0.625633
F13A1	Max_absorbance	0.118221	0.467507	0.829289	0.625633
LRP1	HbA1c	0.118113	0.467919	0.829289	0.625633
FCN2	Clot_lysis_area	0.117811	0.469063	0.829996	0.626165
IGLV1-36	Lag_time_sec	0.117828	0.469	0.829996	0.626165
ATRN	Lag_time_sec	-0.11746	0.470407	0.830882	0.626834
FETUB	Lag_time_sec	-0.11745	0.470445	0.830882	0.626834
SERPINA10	Clot_lysis_area	0.117385	0.470684	0.830882	0.626834
CPN1	HbA1c	0.117089	0.471811	0.832212	0.627837
IGLV2-14	Clot_lysis_area	0.116753	0.473095	0.833815	0.629047
IGKV1-5	Lysis50_time_sec	-0.11658	0.473771	0.834347	0.629448
IGHV3OR16-9	Max_absorbance	-0.11606	0.475723	0.835127	0.630037
IGHV4-4	Max_absorbance	-0.11597	0.47609	0.835127	0.630037

IGKV1-13	Max_absorbance	-0.11608	0.475663	0.835127	0.630037
CASP14	Lag_time_sec	0.115829	0.476628	0.835299	0.630167
CD14	eGDRWC	0.115748	0.476939	0.835299	0.630167
IGHV3-72	Lysis50_time_sec	0.115374	0.478372	0.835869	0.630596
IGLV3-10	eGDRWC	0.115271	0.478766	0.835869	0.630596
LUM	Lysis50_time_sec	0.115542	0.477727	0.835869	0.630596
CASP14	Clot_lysis_area	-0.11503	0.479701	0.836845	0.631333
CLEC3B	Lag_time_sec	-0.11487	0.480324	0.837276	0.631658
IGHV1-3	Max_absorbance	0.114754	0.480756	0.837372	0.631731
PROCR	eGDRWC	0.114501	0.481728	0.838105	0.632283
PROC	HbA1c	0.114285	0.482561	0.838549	0.632619
A0A0G2JRQ6	HbA1c	0.113633	0.485078	0.842265	0.635422
APOC3	Max_absorbance	-0.11352	0.485508	0.842355	0.63549
FN1	eGDRWC	-0.1128	0.488298	0.84312	0.636067
IGHA1	eGDRWC	0.112926	0.487816	0.84312	0.636067
IGKV6-21	Lag_time_sec	-0.11317	0.486858	0.84312	0.636067
IGLV2-14	eGDRWC	0.112724	0.488601	0.84312	0.636067
LTF	HbA1c	-0.11305	0.487345	0.84312	0.636067
IGLV3-10	Max_absorbance	-0.11243	0.489725	0.843923	0.636673
LYZ	Max_absorbance	-0.11241	0.489824	0.843923	0.636673
QSOX1	Max_absorbance	0.112087	0.491077	0.845427	0.637808
ITIH3	Lysis50_time_sec	0.111823	0.492106	0.846544	0.63865
IGHV1-2	Max_absorbance	-0.11139	0.493808	0.848551	0.640164
IGLV1-40	Lysis50_time_sec	-0.11133	0.494035	0.848551	0.640164
ANGPTL6	Clot_lysis_area	-0.11105	0.495106	0.849735	0.641058
IGKV3-20	Lysis50_time_sec	-0.11077	0.496226	0.850707	0.64179
IGHV3OR15-7	Max_absorbance	0.110128	0.49873	0.85398	0.64426
TTR	HbA1c	-0.10998	0.499301	0.8543	0.644501
C1R	Lag_time_sec	0.109661	0.500564	0.855804	0.645636
APOC1	Lysis50_time_sec	0.109359	0.501755	0.855867	0.645683
FCN3	HbA1c	-0.10921	0.502333	0.856196	0.645932
CD5L	Lag_time_sec	-0.10898	0.50323	0.856661	0.646283

© Thieme. All rights reserved.

IGHG1	Max_absorbance	0.108947	0.503375	0.856661	0.646283
APOA2	Lag_time_sec	0.108758	0.504121	0.857048	0.646574
SERPINA3	eGDRWC	-0.10869	0.504372	0.857048	0.646574
GPX3	eGDRWC	0.108116	0.506656	0.858962	0.648018
PIP	HbA1c	0.108189	0.506369	0.858962	0.648018
SERPINA3	Lag_time_sec	-0.10814	0.506567	0.858962	0.648018
ALCAM	Clot_lysis_area	-0.10597	0.515185	0.859213	0.648208
ALDOA	HbA1c	-0.10326	0.526058	0.859213	0.648208
APMAP	Lysis50_time_sec	0.103398	0.525484	0.859213	0.648208
APOC2	Lysis50_time_sec	0.107974	0.507219	0.859213	0.648208
C1QB	Lysis50_time_sec	0.104153	0.522448	0.859213	0.648208
C2	Clot_lysis_area	0.107194	0.510307	0.859213	0.648208
C6	Lysis50_time_sec	0.103107	0.526656	0.859213	0.648208
CAMP	Lysis50_time_sec	-0.10667	0.512395	0.859213	0.648208
DCD	eGDRWC	0.107359	0.509652	0.859213	0.648208
F12	HbA1c	0.10385	0.523666	0.859213	0.648208
F7	Clot_lysis_area	0.103361	0.525633	0.859213	0.648208
FCGR3B	HbA1c	0.104463	0.521206	0.859213	0.648208
FCGR3B	Clot_lysis_area	0.103017	0.527017	0.859213	0.648208
HBA2	Max_absorbance	0.105625	0.516554	0.859213	0.648208
IGFBP3	Max_absorbance	-0.10489	0.519494	0.859213	0.648208
IGHV1-18	eGDRWC	0.104285	0.521918	0.859213	0.648208
IGHV3-72	Lag_time_sec	0.103616	0.524607	0.859213	0.648208
IGHV3-74	Clot_lysis_area	0.105043	0.518879	0.859213	0.648208
IGHV3OR15-7	Lysis50_time_sec	-0.1057	0.516255	0.859213	0.648208
IGHV4-28	Lysis50_time_sec	-0.10358	0.524758	0.859213	0.648208
IGHV5-51	HbA1c	-0.10354	0.524921	0.859213	0.648208
IGKV1-33	eGDRWC	0.106821	0.511788	0.859213	0.648208
IGKV1-39	Max_absorbance	0.1055	0.517052	0.859213	0.648208
IGKV1-8	Lysis50_time_sec	0.104336	0.521715	0.859213	0.648208
IGKV3-11	eGDRWC	0.102964	0.527234	0.859213	0.648208
IGKV3-20	HbA1c	0.106631	0.512547	0.859213	0.648208

IGKV3D-7	Lag_time_sec	-0.10352	0.524982	0.859213	0.648208
IGLV1-40	Clot_lysis_area	-0.10506	0.518793	0.859213	0.648208
IGLV3-21	eGDRWC	0.105077	0.518744	0.859213	0.648208
IGLV8-61	Lag_time_sec	0.106211	0.514217	0.859213	0.648208
ITIH3	eGDRWC	0.103076	0.526783	0.859213	0.648208
ITIH4	eGDRWC	-0.10728	0.509975	0.859213	0.648208
KLKB1	Lysis50_time_sec	0.104925	0.519353	0.859213	0.648208
LCAT	eGDRWC	0.104391	0.521491	0.859213	0.648208
LRG1	Lag_time_sec	-0.10635	0.513652	0.859213	0.648208
MASP2	Max_absorbance	-0.10414	0.522503	0.859213	0.648208
ORM2	eGDRWC	-0.10381	0.523833	0.859213	0.648208
PGLYRP2	eGDRWC	-0.10335	0.525691	0.859213	0.648208
PIGR	eGDRWC	-0.10486	0.519612	0.859213	0.648208
PIGR	Lag_time_sec	0.107197	0.510296	0.859213	0.648208
PKM	Clot_lysis_area	-0.10668	0.512338	0.859213	0.648208
PRDX2	HbA1c	0.104208	0.522226	0.859213	0.648208
PRR4	Lysis50_time_sec	0.10462	0.520573	0.859213	0.648208
SERPINA4	Lysis50_time_sec	0.103064	0.526828	0.859213	0.648208
SERPINA6	Lysis50_time_sec	0.105892	0.51549	0.859213	0.648208
TF	eGDRWC	0.106049	0.514863	0.859213	0.648208
TF	Lag_time_sec	-0.10658	0.512731	0.859213	0.648208
TGFBI	eGDRWC	-0.1072	0.510266	0.859213	0.648208
VASN	Max_absorbance	-0.10478	0.519925	0.859213	0.648208
A0A0J9YY99	Clot_lysis_area	-0.10238	0.529588	0.860484	0.649167
IGLV1-47	HbA1c	0.102401	0.529505	0.860484	0.649167
TTR	Lysis50_time_sec	-0.10244	0.529332	0.860484	0.649167
CRISP3	Lysis50_time_sec	-0.10214	0.530566	0.860816	0.649417
IGHV1-18	HbA1c	-0.10215	0.530508	0.860816	0.649417
B4GALT1	Lag_time_sec	-0.10136	0.533732	0.860862	0.649452
F5	eGDRWC	-0.10127	0.534094	0.860862	0.649452
HBB	eGDRWC	-0.1013	0.533949	0.860862	0.649452
IGHV3OR15-7	Clot_lysis_area	0.101414	0.533502	0.860862	0.649452

IGHV4-34	Max_absorbance	-0.10168	0.53242	0.860862	0.649452
IGKJ1	eGDRWC	0.10167	0.532466	0.860862	0.649452
JCHAIN	Clot_lysis_area	-0.10119	0.534393	0.860862	0.649452
PRDX2	Lysis50_time_sec	0.101955	0.531311	0.860862	0.649452
VCAM1	Max_absorbance	-0.10118	0.534462	0.860862	0.649452
MMP2	Max_absorbance	0.100877	0.535683	0.861582	0.649995
C4A	Max_absorbance	-0.10069	0.536455	0.862202	0.650462
HPR	Max_absorbance	0.100308	0.537996	0.863432	0.651391
CST3	Lysis50_time_sec	-0.09989	0.539709	0.864754	0.652388
IGHV2-5	Lysis50_time_sec	0.099679	0.540563	0.864754	0.652388
IGLV1-51	Max_absorbance	-0.09963	0.540763	0.864754	0.652388
SERPINA3	HbA1c	0.09947	0.541417	0.865179	0.652708
PON1	eGDRWC	0.099276	0.542211	0.865827	0.653197
FN1	Lysis50_time_sec	0.098682	0.544642	0.869085	0.655655
BCHE	Lag_time_sec	-0.09758	0.549166	0.871439	0.657432
IGHV1OR15-1	Lysis50_time_sec	-0.09774	0.548525	0.871439	0.657432
IGKV1-5	Clot_lysis_area	-0.09747	0.549625	0.871439	0.657432
PON1	HbA1c	-0.0979	0.547866	0.871439	0.657432
SELENOP	Lag_time_sec	-0.09781	0.548214	0.871439	0.657432
SERPINA7	eGDRWC	-0.09758	0.549173	0.871439	0.657432
IGKV1-37	Lysis50_time_sec	0.097097	0.551153	0.873215	0.658771
APCS	Clot_lysis_area	0.095384	0.558234	0.873492	0.65898
APOM	eGDRWC	-0.09598	0.555758	0.873492	0.65898
C5	Clot_lysis_area	0.096077	0.555364	0.873492	0.65898
C7	eGDRWC	0.095135	0.559263	0.873492	0.65898
CAMP	Max_absorbance	0.095993	0.555708	0.873492	0.65898
IGHV2-5	Max_absorbance	0.095062	0.559568	0.873492	0.65898
IGHV3-35	Lysis50_time_sec	-0.09691	0.551927	0.873492	0.65898
IGHV3-49	Lysis50_time_sec	-0.09583	0.556366	0.873492	0.65898
IGLV5-45	Lag_time_sec	-0.09538	0.558229	0.873492	0.65898
IL1RAP	Lag_time_sec	0.095902	0.556087	0.873492	0.65898
LILRA3	HbA1c	0.095294	0.558604	0.873492	0.65898

NID1	Max_absorbance	0.096481	0.553694	0.873492	0.65898
PPBP	Max_absorbance	0.096284	0.554509	0.873492	0.65898
RARRES2	eGDRWC	0.096739	0.552627	0.873492	0.65898
SERPINA5	Clot_lysis_area	-0.09562	0.55725	0.873492	0.65898
LPA	HbA1c	0.094945	0.560054	0.873638	0.65909
ALDOA	Lag_time_sec	0.094729	0.56095	0.874422	0.659682
IGKV1-16	Lag_time_sec	-0.09458	0.561553	0.874749	0.659928
APOA1	Clot_lysis_area	-0.09433	0.562618	0.875408	0.660425
C8A	Lag_time_sec	-0.09413	0.563448	0.875408	0.660425
FN1	Clot_lysis_area	-0.09423	0.563036	0.875408	0.660425
IGHV4-4	Lag_time_sec	0.094104	0.563549	0.875408	0.660425
CFHR1	Lysis50_time_sec	0.093842	0.564641	0.875882	0.660783
IGHV3-74	Lysis50_time_sec	-0.09392	0.564336	0.875882	0.660783
DSG1	Clot_lysis_area	-0.09329	0.566948	0.877681	0.66214
FCGR3B	eGDRWC	0.093091	0.567772	0.877681	0.66214
IGHV4-4	Clot_lysis_area	-0.09318	0.567392	0.877681	0.66214
A1BG	Lysis50_time_sec	-0.09281	0.568941	0.878575	0.662815
ABCB9	Clot_lysis_area	0.092764	0.56914	0.878575	0.662815
C8G	Max_absorbance	0.092569	0.569956	0.878882	0.663046
CRTAC1	Max_absorbance	-0.09243	0.570528	0.878882	0.663046
IGFBP3	Lag_time_sec	-0.09225	0.571312	0.878882	0.663046
IGHD	Lag_time_sec	-0.09225	0.571313	0.878882	0.663046
IGLV5-37	Clot_lysis_area	0.092402	0.570654	0.878882	0.663046
SELENOP	Max_absorbance	-0.09141	0.574817	0.882444	0.665733
SELL	eGDRWC	0.091484	0.574508	0.882444	0.665733
APOD	Lag_time_sec	-0.09034	0.579312	0.884931	0.667609
F5	HbA1c	-0.09022	0.579847	0.884931	0.667609
FCN2	HbA1c	0.090308	0.579453	0.884931	0.667609
FCN3	Max_absorbance	0.090358	0.579244	0.884931	0.667609
HPX	Clot_lysis_area	-0.09017	0.58004	0.884931	0.667609
IGHD	Max_absorbance	0.090141	0.580161	0.884931	0.667609
IGHV3-35	eGDRWC	0.090081	0.580413	0.884931	0.667609

PVR	Max_absorbance	-0.09069	0.577826	0.884931	0.667609
SERPING1	Clot_lysis_area	-0.0901	0.580312	0.884931	0.667609
HGFAC	HbA1c	-0.08988	0.581248	0.884992	0.667656
PLG	HbA1c	0.089898	0.581186	0.884992	0.667656
IGHV3-9	Max_absorbance	0.089773	0.581714	0.885096	0.667735
F13A1	HbA1c	-0.0896	0.58246	0.885625	0.668133
IGKV2D-30	Max_absorbance	0.089205	0.584116	0.885723	0.668208
IGLV5-39	Max_absorbance	0.089417	0.583215	0.885723	0.668208
C1S	Clot_lysis_area	0.088848	0.585628	0.88694	0.669125
COL6A3	Lysis50_time_sec	0.088357	0.587707	0.88694	0.669125
IGKV1D-16	Max_absorbance	0.088491	0.58714	0.88694	0.669125
IGLL1	HbA1c	-0.08857	0.586788	0.88694	0.669125
LRG1	Lysis50_time_sec	0.088821	0.585742	0.88694	0.669125
VTN	Max_absorbance	-0.08842	0.587435	0.88694	0.669125
AZGP1	Lag_time_sec	-0.08795	0.589438	0.888348	0.670187
TFRC	Max_absorbance	-0.08795	0.589431	0.888348	0.670187
APOL1	Max_absorbance	0.087852	0.58985	0.888368	0.670202
APOM	Lysis50_time_sec	-0.08687	0.594013	0.889954	0.671399
C4B_2	Clot_lysis_area	-0.08649	0.595667	0.889954	0.671399
F13B	eGDRWC	-0.08675	0.594552	0.889954	0.671399
FCGBP	HbA1c	0.086818	0.594254	0.889954	0.671399
IGHV3-38	eGDRWC	0.087031	0.593346	0.889954	0.671399
IGHV3-72	eGDRWC	-0.08701	0.593457	0.889954	0.671399
IGLV3-9	HbA1c	-0.08671	0.594717	0.889954	0.671399
KNG1	Max_absorbance	0.086445	0.595846	0.889954	0.671399
LILRA3	Lysis50_time_sec	-0.08745	0.591576	0.889954	0.671399
PLTP	Lag_time_sec	-0.08651	0.59555	0.889954	0.671399
SAA2	Lysis50_time_sec	0.086931	0.593775	0.889954	0.671399
ECM1	Lag_time_sec	0.085926	0.598062	0.890976	0.67217
IGF2R	Lysis50_time_sec	-0.08576	0.598787	0.890976	0.67217
MMP2	HbA1c	-0.08585	0.598406	0.890976	0.67217
QSOX1	Clot_lysis_area	0.086014	0.597685	0.890976	0.67217

RBP4	Lysis50_time_sec	-0.08592	0.598098	0.890976	0.67217
IGKV1-5	Lag_time_sec	0.085641	0.599282	0.891117	0.672277
IGKV2D-30	Lag_time_sec	0.085241	0.600997	0.891285	0.672403
IGKV3-20	Lag_time_sec	-0.08546	0.600072	0.891285	0.672403
NID1	Lysis50_time_sec	0.085257	0.600926	0.891285	0.672403
C1RL	HbA1c	0.084981	0.602108	0.891422	0.672506
ITIH2	Lag_time_sec	-0.08494	0.60229	0.891422	0.672506
HSPA5	Clot_lysis_area	-0.08484	0.602722	0.891468	0.672542
C9	eGDRWC	-0.08463	0.603607	0.892184	0.673082
VWF	Max_absorbance	-0.08418	0.60557	0.894492	0.674823
APOC1	Lag_time_sec	-0.08378	0.60726	0.895799	0.675809
IGLV3-27	HbA1c	-0.08361	0.608014	0.896317	0.6762
COMP	Lysis50_time_sec	0.083411	0.608859	0.896968	0.676691
IGHV4-28	Clot_lysis_area	-0.08303	0.610483	0.897205	0.676869
IGKV6D-21	Lysis50_time_sec	-0.08312	0.610125	0.897205	0.676869
LYZ	eGDRWC	-0.08321	0.609725	0.897205	0.676869
PPBP	Clot_lysis_area	0.083	0.610631	0.897205	0.676869
IGHV1-24	HbA1c	-0.08247	0.612936	0.899373	0.678505
IGHV2-26	eGDRWC	-0.08243	0.613072	0.899373	0.678505
IL1RAP	Clot_lysis_area	0.082378	0.613319	0.899373	0.678505
IGHG3	eGDRWC	-0.08189	0.615443	0.900707	0.679512
IGHV3-38	Lag_time_sec	0.081901	0.615379	0.900707	0.679512
IGLV2-18	eGDRWC	0.081897	0.615397	0.900707	0.679512
IGKV3-7	eGDRWC	0.08166	0.616425	0.901552	0.680149
TGFBI	Max_absorbance	0.081367	0.617696	0.902818	0.681104
BCHE	HbA1c	0.081126	0.618741	0.903381	0.681529
ALDOA	Lysis50_time_sec	0.080434	0.621744	0.903721	0.681785
APOF	Lag_time_sec	0.080277	0.622427	0.903721	0.681785
APOM	HbA1c	0.08073	0.620457	0.903721	0.681785
FCGBP	Clot_lysis_area	0.080234	0.622615	0.903721	0.681785
KNG1	HbA1c	-0.08031	0.622267	0.903721	0.681785
LTF	Max_absorbance	-0.08012	0.623105	0.903721	0.681785

MCAM	Lysis50_time_sec	-0.08091	0.619678	0.903721	0.681785
IGHV3-35	Max_absorbance	-0.08001	0.623608	0.903744	0.681803
MASP2	Lag_time_sec	-0.0799	0.624069	0.903824	0.681863
C1QB	Lag_time_sec	0.079522	0.625715	0.905389	0.683044
CFHR5	HbA1c	0.078627	0.629624	0.905389	0.683044
FGB	eGDRWC	-0.0788	0.628851	0.905389	0.683044
GPLD1	Clot_lysis_area	0.078856	0.628622	0.905389	0.683044
HRG	eGDRWC	-0.07885	0.628627	0.905389	0.683044
IGLC3	Clot_lysis_area	-0.07865	0.629522	0.905389	0.683044
LDHA	eGDRWC	-0.07933	0.626566	0.905389	0.683044
RARRES2	Max_absorbance	-0.0791	0.62755	0.905389	0.683044
SERPINC1	HbA1c	0.079276	0.626787	0.905389	0.683044
SERPINF1	Lag_time_sec	0.078749	0.629089	0.905389	0.683044
APOE	eGDRWC	-0.07823	0.631356	0.906247	0.683691
DSG1	eGDRWC	0.078181	0.631574	0.906247	0.683691
ITIH1	Lysis50_time_sec	-0.07826	0.631206	0.906247	0.683691
DBH	HbA1c	0.077248	0.635657	0.906453	0.683847
EFEMP1	eGDRWC	0.077746	0.633475	0.906453	0.683847
IGHA2	eGDRWC	-0.07775	0.633447	0.906453	0.683847
IGKV1-17	Lag_time_sec	0.077252	0.635643	0.906453	0.683847
MCAM	Lag_time_sec	0.077491	0.634595	0.906453	0.683847
PON1	Clot_lysis_area	0.077676	0.633784	0.906453	0.683847
TF	Lysis50_time_sec	-0.07731	0.635395	0.906453	0.683847
IGHV4-4	Lysis50_time_sec	-0.07662	0.638414	0.90757	0.684689
IGKV3-7	Clot_lysis_area	0.076811	0.637578	0.90757	0.684689
IGLV2-14	Max_absorbance	0.076606	0.638479	0.90757	0.684689
LCP1	Clot_lysis_area	0.076772	0.63775	0.90757	0.684689
NRP1	eGDRWC	0.076925	0.637079	0.90757	0.684689
AMBP	Lysis50_time_sec	-0.07605	0.640925	0.908124	0.685107
HBD	Clot_lysis_area	0.075891	0.641624	0.908124	0.685107
LCAT	Lysis50_time_sec	0.075868	0.641724	0.908124	0.685107
LDHA	HbA1c	-0.07606	0.640898	0.908124	0.685107

PRSS3	eGDRWC	0.075941	0.641404	0.908124	0.685107
RNASE4	HbA1c	0.076056	0.640896	0.908124	0.685107
IGKV1D-16	HbA1c	-0.07563	0.642774	0.908711	0.68555
IGKV1-13	Lag_time_sec	0.075201	0.644667	0.910552	0.686939
GP1BA	Max_absorbance	-0.07471	0.646837	0.913037	0.688813
IGKV2-30	HbA1c	-0.07458	0.64743	0.913295	0.689008
PROS1	Lag_time_sec	-0.07359	0.651808	0.918307	0.69279
VCAM1	Lag_time_sec	-0.07337	0.65275	0.918471	0.692913
CPN2	Lag_time_sec	0.072028	0.658726	0.918586	0.693
F13B	Lysis50_time_sec	0.071943	0.659102	0.918586	0.693
FGG	HbA1c	0.071776	0.659847	0.918586	0.693
GAPDH	Max_absorbance	0.072837	0.655128	0.918586	0.693
GC	Lysis50_time_sec	0.072315	0.657448	0.918586	0.693
IGF2R	Max_absorbance	-0.07243	0.656959	0.918586	0.693
IGKV2D-24	Lysis50_time_sec	-0.07314	0.65379	0.918586	0.693
IGLL5	HbA1c	0.072008	0.658815	0.918586	0.693
IGLV9-49	Lag_time_sec	0.072041	0.658667	0.918586	0.693
LDHA	Max_absorbance	-0.07298	0.654484	0.918586	0.693
MASP1	Max_absorbance	0.072184	0.658032	0.918586	0.693
MST1	Lag_time_sec	0.072915	0.654784	0.918586	0.693
PIGR	HbA1c	-0.0729	0.654854	0.918586	0.693
AHSG	eGDRWC	0.070022	0.667672	0.91884	0.693192
AMBP	Max_absorbance	0.0704	0.665985	0.91884	0.693192
APOH	HbA1c	-0.07106	0.663058	0.91884	0.693192
CD14	HbA1c	0.070529	0.665409	0.91884	0.693192
CPQ	HbA1c	0.070429	0.665853	0.91884	0.693192
FCN2	eGDRWC	-0.071	0.663317	0.91884	0.693192
GSN	Clot_lysis_area	-0.07063	0.664957	0.91884	0.693192
HABP2	Lysis50_time_sec	0.070818	0.664118	0.91884	0.693192
HRG	HbA1c	0.070213	0.666818	0.91884	0.693192
IGF2R	eGDRWC	0.070976	0.663412	0.91884	0.693192
IGHV3-49	Lag_time_sec	0.070071	0.667454	0.91884	0.693192

© Thieme. All rights reserved.

IGHV5-51	Lag_time_sec	0.069978	0.667872	0.91884	0.693192
IGKV2D-24	Clot_lysis_area	-0.07015	0.667106	0.91884	0.693192
IGLL5	Max_absorbance	-0.07078	0.664287	0.91884	0.693192
IGLV7-43	Lysis50_time_sec	-0.07088	0.663833	0.91884	0.693192
KNG1	Lysis50_time_sec	0.070401	0.665979	0.91884	0.693192
LYZ	Lag_time_sec	-0.07051	0.665487	0.91884	0.693192
A0A0G2JRQ6	eGDRWC	-0.06963	0.669445	0.918914	0.693247
APOA2	Max_absorbance	-0.0696	0.669577	0.918914	0.693247
CLEC3B	Max_absorbance	0.069779	0.668762	0.918914	0.693247
IGHV3OR16-12	Clot_lysis_area	-0.06971	0.669072	0.918914	0.693247
IGLV3-27	Clot_lysis_area	-0.06907	0.671937	0.921584	0.695262
C1QC	Clot_lysis_area	-0.06869	0.673625	0.921903	0.695503
CDH5	Clot_lysis_area	-0.06869	0.673653	0.921903	0.695503
IGLV5-37	Lysis50_time_sec	-0.06865	0.673826	0.921903	0.695503
ORM2	Max_absorbance	0.068896	0.672717	0.921903	0.695503
COL6A1	Lag_time_sec	-0.06827	0.675543	0.923007	0.696335
RBP4	Clot_lysis_area	-0.06831	0.675366	0.923007	0.696335
SERPINF1	Lysis50_time_sec	0.068192	0.675876	0.923007	0.696335
F12	Clot_lysis_area	0.067949	0.676971	0.923369	0.696608
IGKV1-39	HbA1c	-0.06795	0.676945	0.923369	0.696608
GLIPR2	HbA1c	-0.0678	0.677645	0.923722	0.696875
IGKV6D-21	Max_absorbance	-0.06766	0.67829	0.924036	0.697111
CFD	Lysis50_time_sec	-0.0672	0.680334	0.92446	0.697431
F2	Lysis50_time_sec	0.067115	0.680724	0.92446	0.697431
IGHV3-35	HbA1c	-0.06703	0.681093	0.92446	0.697431
IGKC	Max_absorbance	-0.06712	0.680715	0.92446	0.697431
LCP1	eGDRWC	-0.06736	0.679614	0.92446	0.697431
SELL	HbA1c	0.067316	0.679816	0.92446	0.697431
ABCB9	Lag_time_sec	0.065307	0.688884	0.924877	0.697746
ADAMTS13	Max_absorbance	0.066136	0.685139	0.924877	0.697746
APOC1	Clot_lysis_area	0.065485	0.68808	0.924877	0.697746
CNDP1	Lysis50_time_sec	0.065216	0.689295	0.924877	0.697746

© Thieme. All rights reserved.

F10	Clot_lysis_area	0.065353	0.688678	0.924877	0.697746
GAPDH	eGDRWC	0.065954	0.685962	0.924877	0.697746
IGHV1-24	eGDRWC	0.066389	0.683995	0.924877	0.697746
IGHV1OR15-1	HbA1c	-0.06528	0.688999	0.924877	0.697746
IGHV4-34	Lag_time_sec	0.066377	0.684053	0.924877	0.697746
IGKV1-12	Lag_time_sec	0.065247	0.689158	0.924877	0.697746
IGKV3D-20	Lysis50_time_sec	-0.06587	0.686361	0.924877	0.697746
IGKV6D-21	Clot_lysis_area	-0.06558	0.687636	0.924877	0.697746
IGLV1-36	HbA1c	-0.06578	0.686734	0.924877	0.697746
IGLV3-19	Clot_lysis_area	-0.06655	0.68326	0.924877	0.697746
ITIH2	HbA1c	0.066682	0.682674	0.924877	0.697746
NRP1	Clot_lysis_area	-0.06588	0.686307	0.924877	0.697746
SPP2	Clot_lysis_area	0.066035	0.685592	0.924877	0.697746
IGLV5-45	Max_absorbance	0.064807	0.691148	0.926178	0.698728
ORM2	Clot_lysis_area	-0.06473	0.691513	0.926178	0.698728
CD44	HbA1c	0.064372	0.693118	0.927212	0.699508
THBS4	HbA1c	-0.06446	0.692721	0.927212	0.699508
IGLV3-19	Lysis50_time_sec	-0.06426	0.693647	0.927362	0.699621
IGKV1-6	Lag_time_sec	0.064043	0.694611	0.927538	0.699753
LCP1	HbA1c	0.063901	0.695256	0.927841	0.699982
ADAMTS13	Clot_lysis_area	-0.06257	0.701326	0.928055	0.700143
APOA2	Lysis50_time_sec	0.063017	0.699274	0.928055	0.700143
APOE	Clot_lysis_area	-0.06199	0.703961	0.928055	0.700143
AZGP1	Clot_lysis_area	-0.06206	0.703617	0.928055	0.700143
C7	HbA1c	-0.06237	0.702233	0.928055	0.700143
COLEC11	eGDRWC	-0.06252	0.701527	0.928055	0.700143
F10	Max_absorbance	-0.06347	0.697214	0.928055	0.700143
F2	eGDRWC	-0.06295	0.699593	0.928055	0.700143
FN1	Max_absorbance	0.06185	0.704588	0.928055	0.700143
GPX3	Lag_time_sec	0.063763	0.695885	0.928055	0.700143
IGHV3-9	Clot_lysis_area	0.062459	0.701812	0.928055	0.700143
IGHV4-28	Lag_time_sec	0.062398	0.70209	0.928055	0.700143

IGKJ1	Max_absorbance	0.062134	0.703296	0.928055	0.700143
IGKV1-33	HbA1c	0.062286	0.702603	0.928055	0.700143
IGKV3D-20	HbA1c	-0.06278	0.700352	0.928055	0.700143
IGLV3-27	Lysis50_time_sec	0.061909	0.704321	0.928055	0.700143
IGLV7-43	Max_absorbance	-0.06209	0.703482	0.928055	0.700143
PKM	Lysis50_time_sec	0.063268	0.698133	0.928055	0.700143
SERPINA6	Max_absorbance	-0.06227	0.702672	0.928055	0.700143
COL6A3	eGDRWC	-0.06155	0.705958	0.928907	0.700786
IGHV3-64D	Clot_lysis_area	-0.06117	0.70771	0.929967	0.701586
MMP2	Clot_lysis_area	-0.06126	0.707293	0.929967	0.701586
FBLN1	Lysis50_time_sec	0.060799	0.709386	0.931618	0.702832
ART3	Lag_time_sec	0.059382	0.715874	0.933524	0.704269
B2M	HbA1c	-0.05929	0.716289	0.933524	0.704269
C1QB	HbA1c	0.059721	0.71432	0.933524	0.704269
C3	Lag_time_sec	-0.05934	0.716055	0.933524	0.704269
C9	Lag_time_sec	-0.05976	0.714133	0.933524	0.704269
CFHR4	Lag_time_sec	-0.05941	0.715756	0.933524	0.704269
IGHV3-7	Clot_lysis_area	-0.05958	0.714972	0.933524	0.704269
MASP1	Clot_lysis_area	0.059708	0.71438	0.933524	0.704269
SERPINF1	HbA1c	0.059556	0.715077	0.933524	0.704269
SOD3	Clot_lysis_area	0.059942	0.713307	0.933524	0.704269
VWF	Clot_lysis_area	-0.06004	0.712873	0.933524	0.704269
APOC3	Clot_lysis_area	-0.05868	0.719109	0.934059	0.704673
APOD	HbA1c	0.058993	0.717659	0.934059	0.704673
F2	Lag_time_sec	-0.0589	0.71809	0.934059	0.704673
FCGR3A	eGDRWC	0.058511	0.719873	0.934059	0.704673
IGFBP3	HbA1c	0.058471	0.720057	0.934059	0.704673
IGKV4-1	Lysis50_time_sec	0.058658	0.719197	0.934059	0.704673
SERPINA1	Clot_lysis_area	0.05856	0.719646	0.934059	0.704673
TF	HbA1c	0.058875	0.7182	0.934059	0.704673
IGLV5-37	Lag_time_sec	0.058242	0.721107	0.934457	0.704973
LRP1	Lysis50_time_sec	0.058222	0.721203	0.934457	0.704973

IGKV2-24	Clot_lysis_area	0.057852	0.7229	0.935179	0.705518
SAA4	HbA1c	0.057844	0.722937	0.935179	0.705518
TFRC	eGDRWC	-0.05773	0.72344	0.935179	0.705518
VASN	Clot_lysis_area	-0.0578	0.723118	0.935179	0.705518
CRISP3	Lag_time_sec	0.057435	0.724823	0.935827	0.706007
IGLL1	Lag_time_sec	-0.05732	0.725348	0.935827	0.706007
C8G	Lysis50_time_sec	0.057121	0.726267	0.936115	0.706224
IGF2R	Lag_time_sec	-0.05666	0.728395	0.936689	0.706657
IGHV4-34	Clot_lysis_area	-0.05681	0.727697	0.936689	0.706657
IGHV2-70D	Max_absorbance	-0.05649	0.729186	0.93692	0.706831
LCP1	Lag_time_sec	-0.05644	0.729417	0.93692	0.706831
CRTAC1	HbA1c	-0.05585	0.732138	0.937171	0.707021
GC	Max_absorbance	0.05594	0.731714	0.937171	0.707021
IGHG1	eGDRWC	0.056229	0.73038	0.937171	0.707021
TTR	Max_absorbance	-0.05608	0.731055	0.937171	0.707021
ALDOA	Max_absorbance	-0.05565	0.733039	0.937785	0.707484
CDH13	HbA1c	-0.05524	0.73497	0.938316	0.707885
FCN3	eGDRWC	-0.05511	0.735552	0.938316	0.707885
IGKJ1	Lag_time_sec	-0.05511	0.735562	0.938316	0.707885
IGKV3-7	Max_absorbance	-0.05527	0.734832	0.938316	0.707885
IGLV9-49	HbA1c	0.055123	0.735491	0.938316	0.707885
APOH	Clot_lysis_area	-0.05496	0.736239	0.938642	0.70813
IGHV2-70D	Lysis50_time_sec	-0.05437	0.738995	0.940133	0.709255
QSOX1	Lag_time_sec	-0.05434	0.739098	0.940133	0.709255
TNXB	eGDRWC	0.054397	0.73885	0.940133	0.709255
FETUB	Max_absorbance	0.054075	0.740346	0.941182	0.710046
IGLV5-37	HbA1c	-0.05358	0.742622	0.943537	0.711823
A1BG	Clot_lysis_area	0.052679	0.746822	0.944024	0.712191
F10	Lag_time_sec	0.052846	0.746047	0.944024	0.712191
IGHM	Lag_time_sec	-0.05335	0.743706	0.944024	0.712191
IGLV7-43	Clot_lysis_area	0.052872	0.745925	0.944024	0.712191
PGLYRP2	HbA1c	-0.0531	0.74487	0.944024	0.712191

PROC	eGDRWC	-0.05269	0.74677	0.944024	0.712191
PVR	Lag_time_sec	-0.0533	0.743942	0.944024	0.712191
RBP4	Lag_time_sec	-0.05285	0.746013	0.944024	0.712191
F9	Clot_lysis_area	0.052491	0.747698	0.944059	0.712217
MASP2	Lysis50_time_sec	0.052543	0.747455	0.944059	0.712217
IGHV6-1	Max_absorbance	0.05239	0.748165	0.944113	0.712258
IGHA2	Max_absorbance	-0.052	0.749967	0.94585	0.713569
AZGP1	Lysis50_time_sec	-0.05175	0.751148	0.946803	0.714288
A1BG	eGDRWC	0.051477	0.752418	0.947328	0.714683
APMAP	Clot_lysis_area	-0.05012	0.758724	0.947328	0.714683
APOE	HbA1c	-0.05038	0.757535	0.947328	0.714683
CD5L	Lysis50_time_sec	-0.05131	0.753186	0.947328	0.714683
CFHR4	Lysis50_time_sec	0.050957	0.754842	0.947328	0.714683
CFHR5	Clot_lysis_area	0.0505	0.756972	0.947328	0.714683
CP	eGDRWC	0.050109	0.758798	0.947328	0.714683
CPN1	eGDRWC	-0.05021	0.758346	0.947328	0.714683
CRTAC1	Lysis50_time_sec	-0.05078	0.755648	0.947328	0.714683
FCGR3B	Lag_time_sec	-0.05028	0.758	0.947328	0.714683
IGHV6-1	Clot_lysis_area	-0.05011	0.758799	0.947328	0.714683
IGKV1-39	Clot_lysis_area	-0.05136	0.752974	0.947328	0.714683
IGLV2-18	Lag_time_sec	-0.0506	0.756486	0.947328	0.714683
PROC	Max_absorbance	-0.05039	0.757462	0.947328	0.714683
SERPINF2	HbA1c	0.05118	0.753798	0.947328	0.714683
SPP2	HbA1c	-0.05022	0.758277	0.947328	0.714683
IGHV3-43	Lysis50_time_sec	-0.04966	0.760883	0.949266	0.716145
SAA4	Max_absorbance	0.049594	0.761204	0.949266	0.716145
APOA4	Lysis50_time_sec	-0.04862	0.76574	0.951617	0.717919
C7	Max_absorbance	0.048725	0.76527	0.951617	0.717919
CPN1	Max_absorbance	0.048639	0.76567	0.951617	0.717919
IGHG1	Clot_lysis_area	-0.04842	0.766699	0.951617	0.717919
IGHV3-49	Max_absorbance	0.049062	0.763692	0.951617	0.717919
IGKV3D-20	eGDRWC	-0.04837	0.766937	0.951617	0.717919

IGLV3-19	Lag_time_sec	-0.04875	0.765163	0.951617	0.717919
LUM	Max_absorbance	0.048421	0.766691	0.951617	0.717919
APOM	Max_absorbance	0.048082	0.76828	0.952752	0.718776
ECM1	Max_absorbance	-0.04791	0.769088	0.953223	0.719131
CLU	Lag_time_sec	-0.0475	0.771	0.95347	0.719317
ITIH2	Clot_lysis_area	-0.04762	0.770459	0.95347	0.719317
PKM	Max_absorbance	0.047556	0.770748	0.95347	0.719317
PLG	eGDRWC	0.047671	0.770208	0.95347	0.719317
IGLV2-8	Max_absorbance	-0.04688	0.773903	0.955467	0.720824
IGHD	HbA1c	0.046647	0.775012	0.956013	0.721236
APOA4	Lag_time_sec	0.042564	0.794264	0.956562	0.72165
APOC2	Max_absorbance	0.045458	0.780608	0.956562	0.72165
APOC3	HbA1c	-0.04373	0.788746	0.956562	0.72165
B2M	Lysis50_time_sec	-0.0441	0.787018	0.956562	0.72165
C1QC	Lysis50_time_sec	0.04232	0.795416	0.956562	0.72165
CASP14	HbA1c	0.045916	0.778452	0.956562	0.72165
CDH5	Lysis50_time_sec	-0.04485	0.783466	0.956562	0.72165
COL6A1	Max_absorbance	0.044637	0.784473	0.956562	0.72165
COL6A1	Lysis50_time_sec	0.045228	0.781688	0.956562	0.72165
COL6A3	Max_absorbance	0.042792	0.793182	0.956562	0.72165
FBLN5	Max_absorbance	0.042638	0.793914	0.956562	0.72165
GPLD1	eGDRWC	-0.04321	0.791218	0.956562	0.72165
HP	eGDRWC	-0.04568	0.779542	0.956562	0.72165
HPX	Max_absorbance	0.042832	0.792994	0.956562	0.72165
HSPA6	eGDRWC	-0.04611	0.777531	0.956562	0.72165
ICOSLG	Lysis50_time_sec	-0.04382	0.78835	0.956562	0.72165
IGHG2	Lag_time_sec	-0.0435	0.789829	0.956562	0.72165
IGHV1-69	Lysis50_time_sec	-0.04493	0.783077	0.956562	0.72165
IGHV2-26	HbA1c	-0.04437	0.78575	0.956562	0.72165
IGHV3OR16-12	Lag_time_sec	-0.04234	0.795317	0.956562	0.72165
IGHV3OR16-9	Lag_time_sec	0.043925	0.787833	0.956562	0.72165
IGHV4-34	HbA1c	-0.04346	0.790006	0.956562	0.72165

IGKV1-12	HbA1c	-0.0433	0.790784	0.956562	0.72165
IGKV2-30	Lysis50_time_sec	-0.04609	0.777631	0.956562	0.72165
IGKV3-20	eGDRWC	0.043358	0.790507	0.956562	0.72165
IGLC7	Max_absorbance	-0.04563	0.779779	0.956562	0.72165
IGLV1-36	Clot_lysis_area	-0.04277	0.793277	0.956562	0.72165
KNG1	eGDRWC	-0.04337	0.790465	0.956562	0.72165
LGALS3BP	Max_absorbance	-0.04246	0.794759	0.956562	0.72165
LRP1	Clot_lysis_area	-0.04293	0.792535	0.956562	0.72165
PIGR	Clot_lysis_area	0.044773	0.783831	0.956562	0.72165
PLXDC2	Lag_time_sec	0.043232	0.791106	0.956562	0.72165
PRR4	HbA1c	-0.04411	0.786971	0.956562	0.72165
RNASE4	eGDRWC	0.04474	0.783987	0.956562	0.72165
RNASE4	Lag_time_sec	-0.04617	0.777251	0.956562	0.72165
SELL	Lag_time_sec	-0.04347	0.789965	0.956562	0.72165
TNXB	Lag_time_sec	0.042564	0.794261	0.956562	0.72165
IGHV4-28	Max_absorbance	-0.04197	0.797085	0.957535	0.722384
A0A0G2JRQ6	Max_absorbance	-0.04128	0.800337	0.958914	0.723424
AFM	Max_absorbance	0.041508	0.799262	0.958914	0.723424
CFD	eGDRWC	0.041384	0.799846	0.958914	0.723424
IGKV3D-7	Lysis50_time_sec	0.041488	0.799353	0.958914	0.723424
SHBG	Clot_lysis_area	0.04127	0.800387	0.958914	0.723424
APMAP	Max_absorbance	-0.04031	0.804925	0.96013	0.724342
APOA1	Lag_time_sec	0.039676	0.807948	0.96013	0.724342
CETP	Clot_lysis_area	0.039982	0.806495	0.96013	0.724342
COL6A1	Clot_lysis_area	-0.03959	0.808343	0.96013	0.724342
COMP	HbA1c	-0.0395	0.808804	0.96013	0.724342
CST3	Lag_time_sec	0.03999	0.806461	0.96013	0.724342
ECM1	Lysis50_time_sec	0.040462	0.80422	0.96013	0.724342
HBB	HbA1c	0.040038	0.806232	0.96013	0.724342
IGHV1-3	Lag_time_sec	-0.03986	0.80706	0.96013	0.724342
IGHV3OR15-7	HbA1c	0.03942	0.809166	0.96013	0.724342
IGLL5	Lag_time_sec	0.03962	0.808214	0.96013	0.724342

IGLV1-40	Max_absorbance	0.039996	0.806428	0.96013	0.724342
ITIH1	HbA1c	0.040064	0.806106	0.96013	0.724342
JCHAIN	eGDRWC	0.039903	0.806871	0.96013	0.724342
LYVE1	Lag_time_sec	-0.04072	0.80298	0.96013	0.724342
ALCAM	Lysis50_time_sec	-0.03926	0.809923	0.960516	0.724633
IGHV3-64D	Max_absorbance	-0.03906	0.810854	0.960597	0.724694
PRDX2	Max_absorbance	0.039089	0.810739	0.960597	0.724694
SERPINA3	Clot_lysis_area	0.03887	0.811782	0.961184	0.725137
HSPA6	HbA1c	0.038689	0.812639	0.961619	0.725465
IGHV3-72	HbA1c	-0.03861	0.813013	0.961619	0.725465
A0A0J9YY99	Lysis50_time_sec	-0.0365	0.823057	0.961802	0.725603
ADAMTS13	Lysis50_time_sec	0.037485	0.818374	0.961802	0.725603
AGT	eGDRWC	-0.03616	0.82467	0.961802	0.725603
C1R	HbA1c	0.036794	0.821667	0.961802	0.725603
C4BPB	Lag_time_sec	-0.03692	0.821071	0.961802	0.725603
COLEC11	Lag_time_sec	0.036161	0.824686	0.961802	0.725603
ECM1	HbA1c	-0.03708	0.820317	0.961802	0.725603
F12	Lysis50_time_sec	0.037359	0.818975	0.961802	0.725603
F9	Max_absorbance	0.036696	0.822133	0.961802	0.725603
FCGBP	Lysis50_time_sec	0.036469	0.823215	0.961802	0.725603
FCN3	Lag_time_sec	0.037413	0.818716	0.961802	0.725603
GPLD1	Lag_time_sec	0.036424	0.823431	0.961802	0.725603
HGFAC	Max_absorbance	-0.03733	0.819109	0.961802	0.725603
IGKC	Lag_time_sec	0.038064	0.815615	0.961802	0.725603
IGKV1-27	Lysis50_time_sec	-0.03628	0.824103	0.961802	0.725603
IGKV6D-21	HbA1c	0.038146	0.815224	0.961802	0.725603
IGLC3	Lysis50_time_sec	0.036935	0.820995	0.961802	0.725603
IGLV3-10	HbA1c	0.036661	0.822301	0.961802	0.725603
LCAT	Lag_time_sec	-0.03616	0.824694	0.961802	0.725603
LDHA	Clot_lysis_area	0.037187	0.819792	0.961802	0.725603
MMP2	Lysis50_time_sec	-0.03831	0.81445	0.961802	0.725603
PLG	Clot_lysis_area	0.037968	0.816073	0.961802	0.725603

PTGDS	Lag_time_sec	0.038172	0.815099	0.961802	0.725603
SERPINC1	eGDRWC	-0.03604	0.825266	0.961802	0.725603
SPP2	eGDRWC	0.036077	0.825088	0.961802	0.725603
THBS4	Lysis50_time_sec	-0.03681	0.821584	0.961802	0.725603
APOC1	Max_absorbance	0.034681	0.831756	0.962812	0.726365
CHL1	HbA1c	0.035369	0.828468	0.962812	0.726365
CHL1	Lag_time_sec	0.034886	0.830777	0.962812	0.726365
CRISP3	Clot_lysis_area	-0.03476	0.831378	0.962812	0.726365
IGHG3	Lag_time_sec	0.034823	0.831076	0.962812	0.726365
IGKV4-1	Lag_time_sec	-0.03496	0.830424	0.962812	0.726365
IGLV3-9	Lag_time_sec	-0.03538	0.828398	0.962812	0.726365
MST1	Lysis50_time_sec	-0.03521	0.829228	0.962812	0.726365
RBP4	Max_absorbance	-0.03473	0.831524	0.962812	0.726365
HBA2	HbA1c	0.03442	0.833002	0.963754	0.727076
CHL1	Lysis50_time_sec	-0.03351	0.837335	0.964248	0.727448
FCGR3A	Clot_lysis_area	-0.03368	0.836548	0.964248	0.727448
FCN3	Clot_lysis_area	-0.03398	0.8351	0.964248	0.727448
IGFALS	Max_absorbance	0.033623	0.836817	0.964248	0.727448
IGHV3OR16-9	Clot_lysis_area	-0.03343	0.83776	0.964248	0.727448
IGKV1-8	Lag_time_sec	-0.03374	0.836271	0.964248	0.727448
MMP2	eGDRWC	0.033955	0.835229	0.964248	0.727448
PON3	eGDRWC	0.033538	0.837225	0.964248	0.727448
PVR	Clot_lysis_area	0.034111	0.834482	0.964248	0.727448
IGLV5-37	Max_absorbance	0.033085	0.839393	0.965627	0.728489
A0A0G2JRQ6	Lysis50_time_sec	-0.02948	0.856725	0.965904	0.728697
AMBP	HbA1c	0.032433	0.842521	0.965904	0.728697
APOF	eGDRWC	0.03071	0.850787	0.965904	0.728697
APOM	Lag_time_sec	-0.02932	0.857451	0.965904	0.728697
ATRN	eGDRWC	-0.02883	0.859816	0.965904	0.728697
AZGP1	HbA1c	-0.0305	0.851806	0.965904	0.728697
CD5L	Max_absorbance	0.031058	0.849119	0.965904	0.728697
CETP	Lysis50_time_sec	-0.02924	0.857861	0.965904	0.728697

 © Thieme. All rights reserved.

COLEC11	Lysis50_time_sec	0.030834	0.850192	0.965904	0.728697
COMP	Lag_time_sec	0.0319	0.845076	0.965904	0.728697
CRISP3	Max_absorbance	-0.03189	0.845106	0.965904	0.728697
DBH	Max_absorbance	0.029844	0.85495	0.965904	0.728697
EFEMP1	Lysis50_time_sec	0.030016	0.854125	0.965904	0.728697
F9	Lysis50_time_sec	0.032673	0.841371	0.965904	0.728697
FGB	HbA1c	0.031062	0.849099	0.965904	0.728697
GLIPR2	Clot_lysis_area	-0.02912	0.858424	0.965904	0.728697
HGFAC	Clot_lysis_area	0.028521	0.861317	0.965904	0.728697
HRG	Max_absorbance	-0.03252	0.842121	0.965904	0.728697
HSP90B1	Lysis50_time_sec	0.029306	0.857538	0.965904	0.728697
ICOSLG	HbA1c	-0.03056	0.851527	0.965904	0.728697
IGHD	Lysis50_time_sec	0.030133	0.853562	0.965904	0.728697
IGHV1-2	HbA1c	-0.02866	0.860669	0.965904	0.728697
IGHV4-34	Lysis50_time_sec	-0.03213	0.843967	0.965904	0.728697
IGKV1-12	Max_absorbance	-0.02877	0.860116	0.965904	0.728697
IGKV1-37	Max_absorbance	-0.02993	0.854525	0.965904	0.728697
IGKV3D-7	eGDRWC	0.030777	0.850468	0.965904	0.728697
IGKV4-1	Clot_lysis_area	0.032256	0.843369	0.965904	0.728697
IGLL5	eGDRWC	0.029	0.859013	0.965904	0.728697
IGLV1-47	Lysis50_time_sec	0.031636	0.846343	0.965904	0.728697
IGLV3-10	Lysis50_time_sec	-0.03035	0.852498	0.965904	0.728697
IGLV5-45	Clot_lysis_area	0.031625	0.846395	0.965904	0.728697
IGLV8-61	Clot_lysis_area	-0.02974	0.855471	0.965904	0.728697
IL1RAP	Lysis50_time_sec	-0.0303	0.852778	0.965904	0.728697
LPA	Max_absorbance	-0.02913	0.858371	0.965904	0.728697
LTF	Lag_time_sec	0.028686	0.860524	0.965904	0.728697
MBL2	Clot_lysis_area	0.02855	0.861178	0.965904	0.728697
MST1	Max_absorbance	-0.02887	0.859647	0.965904	0.728697
PCYOX1	Lysis50_time_sec	0.031597	0.846529	0.965904	0.728697
PKM	HbA1c	-0.02902	0.858932	0.965904	0.728697
PKM	Lag_time_sec	0.029362	0.857267	0.965904	0.728697

PON1	Lysis50_time_sec	-0.02868	0.860547	0.965904	0.728697
PTPRJ	Clot_lysis_area	-0.03276	0.840947	0.965904	0.728697
SERPINC1	Lag_time_sec	-0.0294	0.857105	0.965904	0.728697
SHBG	HbA1c	-0.02923	0.857923	0.965904	0.728697
THBS4	eGDRWC	-0.02852	0.861329	0.965904	0.728697
AMBP	Clot_lysis_area	0.02715	0.867924	0.966066	0.728819
APOC2	Clot_lysis_area	-0.02762	0.865643	0.966066	0.728819
F7	HbA1c	-0.02717	0.867816	0.966066	0.728819
F7	Lysis50_time_sec	0.027444	0.866503	0.966066	0.728819
GC	Clot_lysis_area	0.02795	0.864067	0.966066	0.728819
GPLD1	Lysis50_time_sec	-0.02715	0.867946	0.966066	0.728819
IGHG4	Max_absorbance	0.026949	0.868893	0.966066	0.728819
IGKV3D-15	Clot_lysis_area	-0.0274	0.866706	0.966066	0.728819
IGLC3	Max_absorbance	-0.02837	0.862063	0.966066	0.728819
IGLV1-40	Lag_time_sec	-0.02778	0.864884	0.966066	0.728819
IGLV3-27	eGDRWC	0.027671	0.86541	0.966066	0.728819
KLKB1	Max_absorbance	-0.02711	0.868119	0.966066	0.728819
LYZ	Clot_lysis_area	-0.02822	0.862753	0.966066	0.728819
PKM	eGDRWC	0.026778	0.869719	0.966066	0.728819
PROZ	Lag_time_sec	-0.02814	0.863152	0.966066	0.728819
TTR	Lag_time_sec	-0.0268	0.869616	0.966066	0.728819
APOA4	eGDRWC	-0.02667	0.870254	0.966178	0.728904
HBB	Max_absorbance	0.026334	0.871858	0.966369	0.729048
ITIH4	Lag_time_sec	-0.02653	0.870927	0.966369	0.729048
PLTP	Max_absorbance	0.026275	0.872143	0.966369	0.729048
AFM	Clot_lysis_area	0.022638	0.889723	0.966714	0.729309
APOC3	Lysis50_time_sec	0.022775	0.889059	0.966714	0.729309
ATRN	Max_absorbance	0.025901	0.873948	0.966714	0.729309
ATRN	Clot_lysis_area	-0.02151	0.895175	0.966714	0.729309
B2M	Max_absorbance	0.023924	0.883504	0.966714	0.729309
BTD	Max_absorbance	0.025781	0.874527	0.966714	0.729309
C1QC	eGDRWC	-0.02515	0.877567	0.966714	0.729309

C1R	Lysis50_time_sec	0.022927	0.888327	0.966714	0.729309
C5	Lag_time_sec	0.02161	0.894701	0.966714	0.729309
C6	Lag_time_sec	-0.02515	0.877598	0.966714	0.729309
CDH5	eGDRWC	0.025256	0.877062	0.966714	0.729309
CFB	Clot_lysis_area	0.021737	0.894088	0.966714	0.729309
CFD	Lag_time_sec	-0.02266	0.889626	0.966714	0.729309
CNDP1	Max_absorbance	-0.02171	0.89424	0.966714	0.729309
COL6A1	eGDRWC	0.025239	0.877147	0.966714	0.729309
CPQ	Max_absorbance	0.02363	0.884922	0.966714	0.729309
CRTAC1	Clot_lysis_area	-0.02534	0.876669	0.966714	0.729309
CST3	eGDRWC	-0.02501	0.878229	0.966714	0.729309
DCD	Max_absorbance	0.025794	0.874464	0.966714	0.729309
FETUB	Clot_lysis_area	0.02188	0.893396	0.966714	0.729309
FN1	HbA1c	-0.02164	0.894558	0.966714	0.729309
HBD	Max_absorbance	-0.02242	0.890789	0.966714	0.729309
IGHG1	HbA1c	-0.02199	0.892877	0.966714	0.729309
IGHG2	Clot_lysis_area	0.02145	0.895478	0.966714	0.729309
IGHG4	Clot_lysis_area	-0.02468	0.879846	0.966714	0.729309
IGHV3-15	Clot_lysis_area	-0.02184	0.893568	0.966714	0.729309
IGHV3-38-3	Max_absorbance	-0.02195	0.893041	0.966714	0.729309
IGHV3-49	Clot_lysis_area	0.025532	0.875732	0.966714	0.729309
IGHV3-64	eGDRWC	-0.02348	0.885673	0.966714	0.729309
IGHV3OR16-12	HbA1c	-0.02451	0.880659	0.966714	0.729309
IGHV4-4	HbA1c	0.023784	0.884179	0.966714	0.729309
IGKV1-17	Clot_lysis_area	-0.02118	0.89677	0.966714	0.729309
IGKV2-24	Lysis50_time_sec	0.021742	0.894062	0.966714	0.729309
IGKV2-40	Lag_time_sec	0.021322	0.8961	0.966714	0.729309
IGKV6D-21	Lag_time_sec	0.02384	0.883909	0.966714	0.729309
IGLL5	Lysis50_time_sec	-0.02564	0.875193	0.966714	0.729309
IGLV2-14	Lag_time_sec	-0.02371	0.884552	0.966714	0.729309
IGLV7-46	Clot_lysis_area	-0.024	0.883132	0.966714	0.729309
ITIH1	eGDRWC	-0.02436	0.881408	0.966714	0.729309

ITIH3	Lag_time_sec	0.021179	0.896794	0.966714	0.729309
LILRA3	Lag_time_sec	0.021653	0.894495	0.966714	0.729309
LUM	HbA1c	0.023884	0.883694	0.966714	0.729309
PIGR	Max_absorbance	-0.02398	0.88322	0.966714	0.729309
RNASE4	Clot_lysis_area	0.024338	0.881501	0.966714	0.729309
SERPINA4	HbA1c	0.025647	0.875177	0.966714	0.729309
VASN	Lysis50_time_sec	0.021436	0.895546	0.966714	0.729309
FBLN1	HbA1c	0.021036	0.897486	0.966991	0.729518
IGLV5-39	Clot_lysis_area	0.020728	0.898978	0.968131	0.730377
IGLC7	Lysis50_time_sec	0.020515	0.900012	0.968775	0.730863
IGLV2-18	Clot_lysis_area	0.020025	0.902389	0.970865	0.73244
IGLV3-19	eGDRWC	0.019875	0.903115	0.971176	0.732675
CLEC3B	HbA1c	-0.01976	0.903694	0.97127	0.732746
TGFBI	Lag_time_sec	0.019677	0.904075	0.97127	0.732746
MST1	Clot_lysis_area	0.019467	0.905094	0.971427	0.732864
SAA4	eGDRWC	0.019479	0.905035	0.971427	0.732864
DCD	Lysis50_time_sec	-0.0193	0.905918	0.971842	0.733178
CHL1	eGDRWC	0.019148	0.906644	0.971869	0.733198
IGHG3	Clot_lysis_area	-0.01911	0.90685	0.971869	0.733198
TTR	Clot_lysis_area	-0.01902	0.907253	0.971869	0.733198
IGHG1	Lysis50_time_sec	-0.01858	0.909392	0.973029	0.734073
IGHG3	Max_absorbance	-0.01853	0.909646	0.973029	0.734073
SERPINA4	Clot_lysis_area	0.018546	0.909568	0.973029	0.734073
APOM	Clot_lysis_area	0.018222	0.91114	0.973375	0.734334
CNDP1	Clot_lysis_area	0.018173	0.911377	0.973375	0.734334
LILRA3	Clot_lysis_area	0.018103	0.911719	0.973375	0.734334
VCAM1	eGDRWC	-0.01729	0.915667	0.97672	0.736857
A0A0J9YY99	eGDRWC	0.016919	0.917473	0.977643	0.737554
APOE	Max_absorbance	-0.01684	0.917843	0.977643	0.737554
ART3	eGDRWC	-0.01674	0.918352	0.977643	0.737554
CFHR1	Clot_lysis_area	0.016797	0.918063	0.977643	0.737554
ITIH1	Clot_lysis_area	-0.01661	0.918977	0.977841	0.737703

APOD	eGDRWC	-0.01589	0.922492	0.978349	0.738086
COL6A1	HbA1c	0.015879	0.922531	0.978349	0.738086
IGKV1-27	Clot_lysis_area	0.016057	0.921663	0.978349	0.738086
IGLL1	eGDRWC	0.016012	0.921884	0.978349	0.738086
LCP1	Lysis50_time_sec	0.016174	0.921094	0.978349	0.738086
MBL2	Max_absorbance	0.015956	0.922153	0.978349	0.738086
QSOX1	Lysis50_time_sec	0.016268	0.920637	0.978349	0.738086
C8G	Lag_time_sec	0.015688	0.923457	0.978398	0.738124
LYVE1	eGDRWC	-0.0157	0.923409	0.978398	0.738124
APOD	Clot_lysis_area	-0.01541	0.924789	0.978602	0.738277
CDH13	eGDRWC	0.015378	0.924968	0.978602	0.738277
PCYOX1	HbA1c	-0.01551	0.92431	0.978602	0.738277
DCD	Clot_lysis_area	-0.01507	0.926448	0.978772	0.738406
F7	eGDRWC	0.015182	0.925918	0.978772	0.738406
FBLN5	Clot_lysis_area	-0.01511	0.926279	0.978772	0.738406
IGHV3OR16-12	Lysis50_time_sec	0.01464	0.928555	0.979604	0.739033
IGLV2-18	HbA1c	0.014713	0.928202	0.979604	0.739033
IGLV3-19	Max_absorbance	-0.0148	0.927771	0.979604	0.739033
F2	Clot_lysis_area	0.01441	0.929676	0.979857	0.739224
IGLV1-47	eGDRWC	-0.01443	0.929593	0.979857	0.739224
SERPINA6	Clot_lysis_area	-0.01423	0.930532	0.980296	0.739555
HSPA6	Lag_time_sec	-0.01408	0.93126	0.980599	0.739783
C7	Lag_time_sec	-0.01394	0.931988	0.980901	0.740012
A1BG	HbA1c	-0.0138	0.93264	0.981123	0.740179
IGLV1-51	Clot_lysis_area	0.013474	0.934234	0.981872	0.740744
SAA2	Lag_time_sec	0.013217	0.935485	0.982722	0.741386
CFHR5	eGDRWC	-0.01305	0.936286	0.98287	0.741497
IGKV3-20	Max_absorbance	-0.01274	0.937833	0.98287	0.741497
IGKV3D-20	Clot_lysis_area	-0.01282	0.93741	0.98287	0.741497
SOD3	Max_absorbance	-0.01278	0.937597	0.98287	0.741497
C1QC	HbA1c	0.012381	0.939558	0.983153	0.74171
IGHA2	Clot_lysis_area	-0.01223	0.940311	0.983153	0.74171

IGHV3-9	HbA1c	0.01254	0.938782	0.983153	0.74171
IGKV1-6	HbA1c	0.012276	0.94007	0.983153	0.74171
PRR4	eGDRWC	0.012387	0.939528	0.983153	0.74171
CP	Lysis50_time_sec	-0.012	0.941398	0.983746	0.742158
DCD	Lag_time_sec	0.011566	0.94353	0.983746	0.742158
IGLV9-49	Clot_lysis_area	-0.01158	0.94346	0.983746	0.742158
PTPRJ	eGDRWC	-0.01165	0.943104	0.983746	0.742158
A0A0G2JRQ6	Lag_time_sec	0.009676	0.952746	0.983953	0.742314
B4GALT1	Max_absorbance	0.009917	0.951574	0.983953	0.742314
B4GALT1	Lysis50_time_sec	0.009859	0.951855	0.983953	0.742314
CLU	HbA1c	0.011151	0.945554	0.983953	0.742314
CRTAC1	eGDRWC	0.010591	0.948286	0.983953	0.742314
CST3	HbA1c	-0.00963	0.952963	0.983953	0.742314
DSG1	HbA1c	-0.00955	0.953386	0.983953	0.742314
EFEMP1	Lag_time_sec	0.010401	0.949211	0.983953	0.742314
FBLN1	eGDRWC	-0.0105	0.948745	0.983953	0.742314
IGHM	Lysis50_time_sec	-0.01021	0.950132	0.983953	0.742314
IGHV2-26	Lysis50_time_sec	-0.00953	0.953453	0.983953	0.742314
IGHV3-35	Clot_lysis_area	0.010096	0.950699	0.983953	0.742314
IGHV3-38	HbA1c	0.010028	0.951029	0.983953	0.742314
IGKV1-37	Clot_lysis_area	-0.01069	0.947792	0.983953	0.742314
IGKV1-39	Lag_time_sec	-0.01098	0.946367	0.983953	0.742314
PLG	Max_absorbance	-0.00956	0.953334	0.983953	0.742314
PROZ	Lysis50_time_sec	-0.01074	0.947538	0.983953	0.742314
SERPINA1	Max_absorbance	0.010078	0.950784	0.983953	0.742314
VCAM1	Clot_lysis_area	0.010494	0.948758	0.983953	0.742314
IL1RAP	eGDRWC	-0.00932	0.954504	0.984581	0.742788
SERPINF2	eGDRWC	0.009159	0.955272	0.984916	0.743041
BCHE	Lysis50_time_sec	0.008885	0.956605	0.985835	0.743734
AZGP1	eGDRWC	-0.00858	0.958083	0.986574	0.744291
CAMP	HbA1c	0.007727	0.962256	0.987253	0.744804
IGHV4-4	eGDRWC	-0.00771	0.962319	0.987253	0.744804

IGLV2-14	HbA1c	0.008104	0.960419	0.987253	0.744804
PRR4	Lag_time_sec	-0.00792	0.961305	0.987253	0.744804
PRSS3	Lysis50_time_sec	0.007694	0.962417	0.987253	0.744804
SERPINA7	Lag_time_sec	0.007735	0.962221	0.987253	0.744804
C8B	Lag_time_sec	-0.00738	0.96395	0.987965	0.745341
SERPINA4	Lag_time_sec	0.00737	0.963998	0.987965	0.745341
IGHG1	Lag_time_sec	0.007088	0.965378	0.988924	0.746064
CDH5	HbA1c	0.006882	0.966382	0.989002	0.746123
GAPDH	Clot_lysis_area	-0.00689	0.966341	0.989002	0.746123
IGHV3-64	Max_absorbance	0.006526	0.96812	0.989002	0.746123
IGKC	Clot_lysis_area	0.006571	0.967902	0.989002	0.746123
VWF	Lag_time_sec	0.00674	0.967076	0.989002	0.746123
AZGP1	Max_absorbance	0.006111	0.970147	0.989298	0.746346
IGHA2	HbA1c	-0.0061	0.970187	0.989298	0.746346
IGHV1-3	Clot_lysis_area	-0.00624	0.969504	0.989298	0.746346
THBS4	Lag_time_sec	0.006338	0.969039	0.989298	0.746346
CFHR1	Lag_time_sec	-0.00596	0.970891	0.989562	0.746546
IGKV3-7	HbA1c	0.005674	0.972283	0.990527	0.747274
PON3	Lysis50_time_sec	-0.00517	0.974744	0.992581	0.748823
IGHV1-45	eGDRWC	0.004723	0.976927	0.993894	0.749813
LGALS3BP	Lysis50_time_sec	-0.00474	0.976839	0.993894	0.749813
LGALS3BP	Clot_lysis_area	0.004401	0.978496	0.995035	0.750675
DSG1	Max_absorbance	0.004241	0.979282	0.99538	0.750935
AHSG	Lysis50_time_sec	0.003983	0.980542	0.995881	0.751313
B4GALT1	Clot_lysis_area	-0.00391	0.980879	0.995881	0.751313
IGHV2-70D	Clot_lysis_area	0.003865	0.981117	0.995881	0.751313
APOF	Clot_lysis_area	0.003684	0.981999	0.99629	0.751622
IGKV3D-15	Lag_time_sec	-0.0036	0.982416	0.99629	0.751622
IGKV3D-15	Max_absorbance	0.003357	0.983597	0.996395	0.7517
MAN1A1	Lag_time_sec	-0.0033	0.983889	0.996395	0.7517
PTPRJ	Lysis50_time_sec	0.003211	0.984309	0.996395	0.7517
SERPINA7	Clot_lysis_area	0.003412	0.98333	0.996395	0.7517

KLKB1	Clot_lysis_area	0.003112	0.984795	0.996434	0.75173
IGKV1-16	HbA1c	-0.00294	0.98566	0.996855	0.752048
APOA4	HbA1c	0.002736	0.986631	0.997106	0.752237
APOH	Max_absorbance	-0.00261	0.987262	0.997106	0.752237
LUM	Clot_lysis_area	-0.00252	0.9877	0.997106	0.752237
SAA4	Clot_lysis_area	0.002419	0.988182	0.997141	0.752264
LTF	Lysis50_time_sec	-0.00228	0.988883	0.997396	0.752456
AMBP	Lag_time_sec	-0.00185	0.990947	0.997517	0.752547
GSN	eGDRWC	0.002015	0.990155	0.997517	0.752547
HSPA5	Lag_time_sec	-0.00187	0.990851	0.997517	0.752547
IGKV1-16	eGDRWC	0.002023	0.990117	0.997517	0.752547
SERPING1	Max_absorbance	-0.00179	0.991244	0.997517	0.752547
APOH	eGDRWC	0.001589	0.992235	0.997613	0.752619
PRSS3	HbA1c	0.001645	0.991964	0.997613	0.752619
IGHG2	Max_absorbance	0.001308	0.99361	0.998277	0.753121
TFRC	Clot_lysis_area	0.00127	0.993793	0.998277	0.753121
APOC4	Clot_lysis_area	-0.00074	0.996371	0.998481	0.753274
CHL1	Clot_lysis_area	0.001018	0.995025	0.998481	0.753274
IGHV1-46	Lag_time_sec	-0.00078	0.996207	0.998481	0.753274
IGHV3-74	Max_absorbance	0.000615	0.996994	0.998481	0.753274
IGLL1	Lysis50_time_sec	0.000795	0.996116	0.998481	0.753274
IGLV2-8	Clot_lysis_area	0.001047	0.994886	0.998481	0.753274
HSPG2	Lag_time_sec	-0.00037	0.99817	0.998824	0.753533
IGKV1-17	Lysis50_time_sec	-0.00033	0.998375	0.998824	0.753533
LYZ	HbA1c	6.45E-05	0.999685	0.999685	0.754182
